# Investigation of the Anti-Inflammatory Activity of Fusaproliferin Analogues Guided by Transcriptome Analysis

**DOI:** 10.3389/fphar.2022.881182

**Published:** 2022-05-05

**Authors:** Qi-Xuan Kuang, Li-Rong Lei, Qing-Zhou Li, Wan Peng, Yu-Mei Wang, Yi-Fei Dai, Dong Wang, Yu-Cheng Gu, Yun Deng, Da-Le Guo

**Affiliations:** ^1^ State Key Laboratory of Southwestern Chinese Medicine Resources, School of Pharmacy, Chengdu University of Traditional Chinese Medicine, Chengdu, China; ^2^ School of Basic Medical Sciences, Chengdu University of Traditional Chinese Medicine, Chengdu, China; ^3^ Institute of Rare Diseases, West China Hospital of Sichuan University, Chengdu, China; ^4^ Department of Basic Medical Sciences, School of Medicine, Tsinghua University, Beijing, China; ^5^ Syngenta Jealott’s Hill International Research Centre, Berkshire, United Kingdom

**Keywords:** *Fusarium proliferatum*, fusaproliferin analogues, anti-inflammatory activity, transcriptome analysis, surface plasmon resonance assays

## Abstract

**Background:** Excessive inflammation results in severe tissue damage as well as serious acute or chronic disorders, and extensive research has focused on finding new anti-inflammatory hit compounds with safety and efficacy profiles from natural products. As promising therapeutic entities for the treatment of inflammation-related diseases, fusaproliferin and its analogs have attracted great interest. However, the underlying anti-inflammatory mechanism is still poorly understood and deserves to be further investigated.

**Methods:** For the estimation of the anti-inflammatory activity of fusaproliferin (**1**) and its analogs (**2**-**4)** *in vitro* and *in vivo*, lipopolysaccharide (LPS)-induced RAW264.7 macrophages and zebrafish embryos were employed. Then, transcriptome analysis was applied to guide subsequent western blot analysis of critical proteins in related signaling pathways. Surface plasmon resonance assays (SPR) combined with molecular docking analyses were finally applied to evaluate the affinity interactions between **1**-**4** and TLR4 and provide a possible interpretation of the downregulation of related signaling pathways.

**Results: 1**-**4** significantly attenuated the production of inflammatory messengers, including nitric oxide (NO), reactive oxygen species (ROS), interleukin-6 (IL-6), tumor necrosis factor-α (TNF-α), and interleukin-1β (IL-1β), as well as nitric oxide synthase (iNOS) and cyclooxygenase-2 (COX-2), in LPS-induced RAW264.7 macrophages. Transcriptome analyses based on RNA-seq indicated the ability of compound **1** to reverse LPS stimulation and the nuclear factor kappa-B (NF-κB) and mitogen-activated protein kinase (MAPKs) signaling pathways contribute to the anti-inflammatory process. Experimental verification at the protein level revealed that **1** can inhibit the activation of inhibitor of NF-κB kinase (IKK), degradation of inhibitor of NF-κB (IκB), and phosphorylation of NF-κB and reduce nuclear translocation of NF-κB. **1** also decreased the phosphorylation of MAPKs, including p38, extracellular regulated protein kinases (ERK), and c-Jun N-terminal kinase (JNK). SPR assays and molecular docking results indicated that **1**-**4** exhibited affinity for the TLR4 protein with KD values of 23.5–29.3 μM.

**Conclusion:** Fusaproliferin and its analogs can be hit compounds for the treatment of inflammation-associated diseases.

## Introduction

Inflammation is generally accepted as a protective response to various harmful stimuli and contributes to the recovery of tissue damage. For instance, lipopolysaccharides (LPS) derived from gram-negative bacteria can bind to toll-like receptor 4 (TLR4) resulting in strong immune responses. These immune responses are mainly mediated by the upregulation of nuclear factor kappa-B (NF-κB) and mitogen-activated protein kinase (MAPK) signaling pathways and the subsequent secretion of proinflammatory mediators, including nitric oxide (NO), interleukin-6 (IL-6), interleukin-1β (IL-1β), reactive oxygen species (ROS) and tumor necrosis factor-α (TNF-α), to maintain homeostasis (Li et al., 2017; Attiq et al., 2018; Liu et al., 2019). However, excessive inflammation usually results in severe tissue damage, cytokine storms and further organ dysfunction, and serious acute or chronic disorders (Atri et al., 2018; Huang et al., 2019). Small molecules suppress TLR4-related signaling pathways and may thus be an effective strategy for the treatment of excessive inflammation (Romerio and Peri, 2020). To date, there are still increasing requirements to develop new anti-inflammatory drugs, and natural products are promising sources of hit compounds with anti-inflammatory activity (Pham et al., 2019; Dona et al., 2020). Meanwhile, natural products have many advantages such as easy availability, high biocompatibility, and low cost.

Sesterterpenoids, an important class of terpenoids with 25 carbon frameworks derived from five isoprene units (Liu et al., 2016), exhibit remarkable biological properties involving cytotoxic, anti-inflammatory, antimicrobial, and antifeedant properties and display a vital role in drug development (Chadwick et al., 2013; Guo et al., 2021; Li and Gustafson, 2021). Among these sesterterpenoids, fusaproliferin and its analogs are of interest as so-called promising therapeutic entities for the treatment of inflammation-related diseases due to their capacity to suppress nitric oxide generation (Huang et al., 2016; Lai et al., 2017; Peng et al., 2020; Jiang et al., 2021). However, its mechanism of action is still poorly understood and deserves further investigation. Analysis on the basis of transcriptome is an effective way to provide the underlying mechanism of natural products, which may give a more practical guide to the investigation of anti-inflammatory mechanisms of fusaproliferin and its analogs.

**FIGURE 1:**
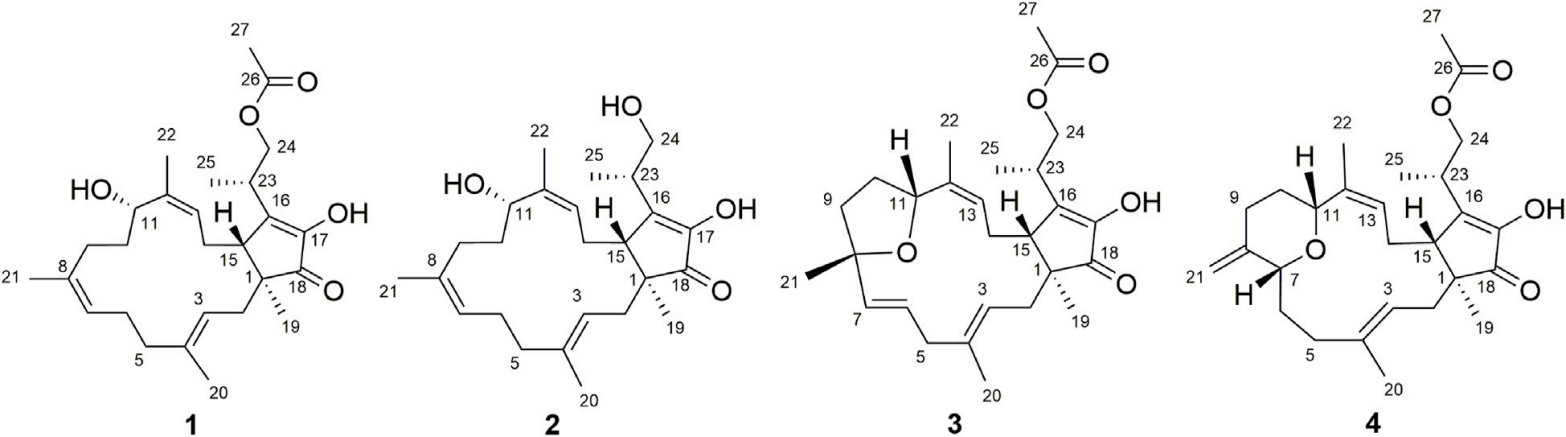
Chemical structures of **1–4**.

As a part of our series of studies investigating natural products with anti-inflammatory activity from endophytes of characteristic Chinese medicines in southwestern China (Feng et al., 2020; Guo et al., 2020; Li et al., 2020), fusaproliferin (**1**) (Nihashi et al., 2002), 11-epiterpestacin (**2**) (Nihashi et al., 2002), fusaprolifins A (**3**) (Liu et al., 2013) and fusaprolifins B (**4**) (Liu et al., 2013) were isolated from *Fusarium proliferatum* (Figure 1), which was obtained as a symbiotic fungus of *Cordyceps sinensis*. In the current manuscript, LPS-induced Raw264.7 macrophages and zebrafish embryos were utilized to assess the anti-inflammatory properties of the abovementioned compounds, and RNA-seq combined with Western blotting was used to reveal the underlying mechanism of this type of compound. Transcriptome analyses indicated that **1** can suppress signaling pathways related to inflammation involved in NF-κB and MAPK signaling pathways mediated by TLR4, which was experimentally confirmed at the protein level. Furthermore, the results of the SPR and molecular docking assays indicate that affinity interactions between **1** and TLR4 might be a potential interpretation for the downregulation of the aforementioned signaling pathways. These results suggest that fusaproliferin has potential as a candidate for TLR4-mediated inflammation-associated diseases.

## Materials and Methods

### General Experimental Procedures

NMR data were obtained using a Bruker-Ascend-400 spectrometer and Bruker-Ascend-700 spectrometer. The semi-preparative purification of all compounds was performed by an NP7000 instrument equipped with a U3000 UV detector using a Kromasil RP-C18 column (10 mm × 250 mm, 5 μm).

### Fungal Material and Fermentation

The fungal strain *F. proliferatum* was obtained from the *Cordyceps sinensis*. Experimental procedures are described in our previous work (Li et al., 2019). According to the molecular biological protocol, the strain (GenBank accession No. OL548909) was identified as *Fusarium proliferatum* and deposited in the Chengdu University of TCM. The fermentation protocol was to incubate in 250 tissue culture flasks (each 330 ml flask containing 40 g brown rice and 2 g peptone) for 30 days.

### Extraction and Isolation of Compounds 1-4

The crude extract was obtained by extracting the ferment with methanol and further extracted by ethyl acetate (EtOAc). The EtOAc extract was divided into six parts by silica gel column chromatography (A-F, eluting stepwise with petroleum ether-acetone 100:0 to 50:50, V/V). The silica gel RP-18 was adopted for the subseparation of fraction E and fraction F, which was divided into subfractions E_1_-E_14_ and subfractions F_1_-F_12_ (300–400 mesh, eluting stepwise with MeOH-H_2_O: 50:50 to 100:0, V/V). Compound **1** (21.8 mg, MeOH-H_2_O, 85:15, t_R_ = 10.4 min, in subfraction E_10_), **2** (7.2 mg, MeOH-H_2_O, 82:18, t_R_ = 12.5 min, in subfraction E_8_), **3** (5.0 mg, MeOH-H_2_O, 62:38, t_R_ = 20.9 min, in subfraction E_5_), and **4** (4.2 mg, MeOH-H_2_O, 70:30, t_R_ = 12.9 min, in subfraction E_7_) were finally obtained using semipreparative HPLC.

### Cell Culture

RAW264.7 macrophages were incubated in DMEM with 10% fetal bovine serum and 1% antibiotics and then maintained in a humidified 5% CO_2_ incubator at 37°C.

### Cell Viability Assay

RAW264.7 macrophages were seeded at a density of 8×10^3^ cells/well (96-well plate), treated with DMSO, LPS, compounds-30 μM, compounds-60 μM, LPS + compounds-30 μM, LPS + compounds-60 μM for 24 h, respectively. and then measured by the 3-(4,5-dimethylthiazol-2-yl)-2,5-diphenyl tetrazolium bromide (MTT) assay. Finally, the absorbance of the 96-well plates was measured by a SpectraMax Plus 384 Universal microplate reader at 570 nm.

### NO Inhibitory Assay in LPS-Stimulated RAW264.7 Macrophages

RAW264.7 macrophages were treated with DMSO, dexamethasone, and **1-4** for 1 h (the negative control group: DMSO, the positive control group: 30 μM dexamethasone, the treatment group: **1-4** at 7.5, 15, 30 μM) and then treated with 1 μg/ml LPS for another 24 h. NO production by RAW264.7 macrophages was detected by the Griess reagent system.

### Assessment of Proinflammatory Cytokines in LPS-Stimulated RAW264.7 Macrophages

RAW264.7 macrophages (4 × 10^5^ cells/well, 12-well plate) were incubated overnight and subsequently pretreated with 1 ml medium containing DMSO, dexamethasone or **1-4** for 1 h and incubated with LPS (1 μg/ml) for another 24 h. The centrifuged culture medium was used to assess the levels of proinflammatory cytokine TNF-α, IL-6, and IL-1β by ELISA according to the manufacturer’s instructions.

### Intracellular ROS Accumulation in LPS-Stimulated RAW264.7 Macrophages

RAW264.7 macrophages (1 × 10^6^ cells/well, 6-well plate) were cultured overnight and subsequently pretreated with 1 ml medium containing DMSO, dexamethasone or **1** for 1 h and incubated with LPS (1 μg/ml) for another 24 h. Then, cells loaded with 2ʹ,7ʹ-dichlorodihydrofluorescein diacetate (DCFH-DA) were measured by flow cytometry (BD FACSVerse, American) and confocal microscopy (Olympus FV1200, Japan).

### Production of NO and ROS in LPS-Stimulated Zebrafish Embryos

As an *in vivo* model, LPS-induced zebrafish embryos were selected to evaluate the anti-inflammatory activities of **1** as presented in a previous study (Wu et al., 2020). Zebrafish embryos were exposed to **1** at concentrations of 7.5, 15, and 30 μM for 1 h and then stimulated with exposure solution containing LPS (10 μg/ml), except the control embryos. The exposure solution was refreshed every 24 h. After incubation for 72 h at 28°C, embryos were transferred to a new 24-well plate and treated with DCFH-DA solution (20 μg/ml) for 1 h and DAF-FMDA (5 μM) for 2 h in the dark at 28°C. The larvae were rinsed with fresh media, anesthetized with tricaine and observed under a laser confocal microscope (Olympus FV1200, Japan). Using ImageJ software to quantify the photographs of the fluorescence intensity with individual zebrafish embryos.

### RNA-Seq Analysis

The NO and proinflammatory cytokine assays revealed that **1-4** had anti-inflammatory activity at a concentration of 30 μM. Seeded the RAW264.7 macrophages (1 × 10^6^ cells/well, 6-well plate) and then pretreated with **1** at 30 μM for 1 h before adding LPS (1 μg/ml) for 24 h as the treatment group (treated with 0.1% DMSO for 24 h as the control group; stimulated with 1 μg/ml LPS for 24 h as the model group). Using TRIzol reagent (Invitrogen) to extract the total RNA of cells and conducting the RNA-seq by an Illumina Novoseq 6000 sequencer. Quality control of raw data was performed with the FastQC tool. HISAT2 software was used to compare the reference genome with the clean reads. Raw sequence data are available on the sequence read archive (SPA) database under accession number: PRJNA792467.

### Western Blotting Assay

RAW264.7 macrophages were plated into 6-well plates overnight, pretreated with 2 ml medium containing DMSO (0.1%), dexamethasone (30 μM) or **1** at concentrations of 7.5, 15, and 30 μM for 1 h and incubated with LPS (1 μg/ml). The details of protein sample extraction and experimental procedures are described in our previous work (Ju et al., 2021).

### Translocation of NF-κB/p65

RAW264.7 macrophages were plated into 12-well plates overnight, treated with DMSO (0.1%), **1** (7.5, 15, 30 μM) or dexamethasone (30 μM) for 1 h and then stimulated with 1 μg/ml LPS for 2 h. NF-κB activation was assessed by detecting the levels of nuclear translocation of NF-κB (NF-κB activation-nuclear translocation assay kit, SN368, Beyotime Biotechnology). Fluorescence detection was performed under a confocal microscope (Olympus FV1200, Japan).

### Molecular Docking to TLR4 Protein

Crystal structures of the TLR4 complex were obtained from the RCSB Protein Data Bank (PDB ID: 3FXI). Docking analysis for **1**-**4** with TLR4 was performed using Schrödinger software. Protein pretreatment, regenerative state of native ligands, H-bond assignment optimization, protein energy minimization and water removal were performed in the Protein Preparation Wizard module. The optimal binding sites were predicted by the SiteMap module.

### Surface Plasmon Resonance Assay

The TLR4 protein (Sino Biological, China) was immobilized on a CM5 sensor chip in sodium acetate (pH = 4.5) according to the manufacturer’s instructions for the amine coupling method. **1**-**4** were injected into a TLR4 protein sensor surface according to the multicycle kinetic protocol (association and dissociation times were 180 and 200 s at a flow rate of 30 μL/min) in PBS containing 5% DMSO. After solvent correction, the affinity constant KD was performed using Biacore T200 evaluation software.

### Statistical Analysis

The data were analysed by GraphPad Prism 7.0 software (San Diego, CA, United States) and then expressed as the mean ± SD (standard deviation) of triplicate measurements of the same experiment. The data were collected from three independent experiments.

## Results

### Compounds 1-4 Inhibited the Release of LPS-Induced NO and Proinflammatory Cytokines

**FIGURE 2:**
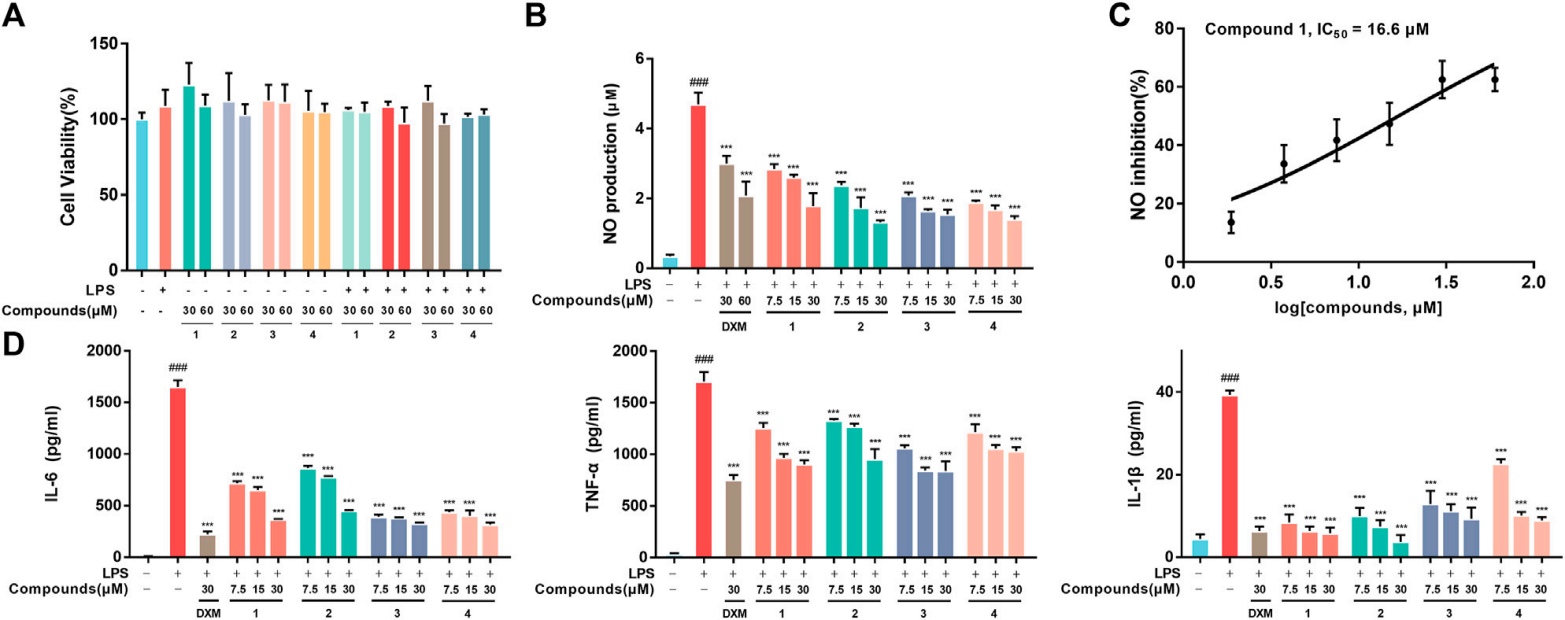
**(A)** Cell viability. RAW264.7 macrophages were treated with DMSO, LPS, compounds-30 μM, compounds-60 μM, LPS + compounds-30 μM, LPS + compounds-60 μM for 24 h, respectively. **(B)** Inhibition of LPS-induced NO production by 1-4 in LPS-stimulated RAW264.7 macrophages. **(C)** The IC_50_ value of compound **1** against LPS-stimulated NO production in RAW264.7 macrophages. **(D)** Inhibition of LPS-stimulated TNF-α, IL-6 and IL-1β production in LPS-stimulated RAW264.7 macrophages. Data are expressed as the mean ± SD. ###*p* < 0.001, compared with the normal control group. ***p < 0.001, compared with the LPS group.

RAW264.7 macrophages were first employed to evaluate the proinflammatory mediator inhibition of **1**-**4** *in vitro*. The MTT results indicated that **1-4** did not show significant cytotoxicity at the administered concentration against RAW264.7 macrophages (Figure 2A). Moreover, **1-4** significantly inhibited the excretion of NO, especially **1,** with an IC_50_ value of 16.6 μM (Figure 2B). Additionally, ELISA analysis revealed that **1**–**4** decreased the secretion of IL-6, TNF-α and IL-1β in a concentration-dependent manner in LPS-stimulated RAW264.7 macrophages (Figure 2C).

### Compounds 1-4 Inhibited the LPS-Induced Protein Expression of iNOS and COX2 and Intracellular ROS Accumulation

**FIGURE 3:**
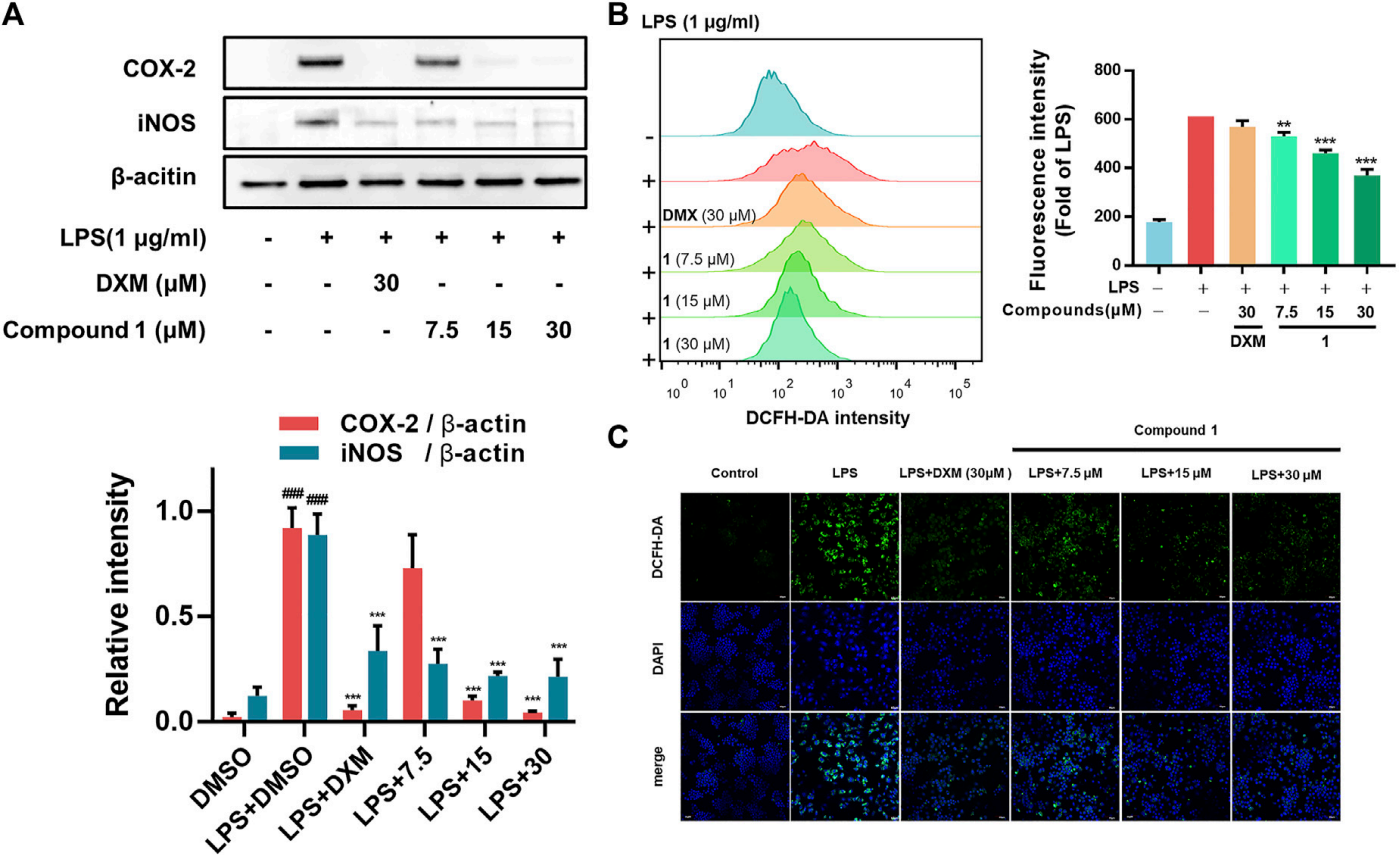
**(A)** Effects of **1** on LPS-induced COX-2 and iNOS levels in RAW264.7 macrophages. RAW264.7 macrophages were pretreated with the tested compounds (7.5, 15, and 30 μM) and dexamethasone (30 μM) as the positive control **(B)** RAW264.7 macrophages were pretreated with **1** for 1 h and then exposed to 1 μg/ml LPS for 24 h. Cells that were labelled with DCFH-DA were evaluated by flow cytometry. The geometric mean fluorescence intensity is expressed as the mean ± SD. **(C)** The protective effect of **1** in LPS-treated RAW264.7 macrophages. Green fluorescence indicating intracellular ROS stained by DCFH-DA. RAW264.7 macrophages were pretreated with **1** for 1 h and exposed to 1 μg/ml LPS for 24 h. Data are expressed as the mean ± SD. ###*p* < 0.001, compared with the normal control group. **p < 0.01, and ***p < 0.001, compared with the LPS group.

In addition to regulating the production of inflammatory cytokines, LPS stimulation induces macrophages to commonly overexpress COX-2 (which regulates prostaglandin production) and iNOS (which generates NO) (Miletic et al., 2007). In addition, as the essential components of the innate immune response against intracellular bacteria in the inflammatory process, ROS can enhance the bactericidal activity of macrophages (West et al., 2011). Our results from Western blotting assays and flow cytometry analysis revealed that **1** can significantly decrease the expression of iNOS and COX-2 (Figure 3A) and the accumulation of ROS (Figure 3B) in LPS-induced RAW264.7 macrophages, which was further confirmed by confocal microscopy imaging (Figure 3C).

### Anti-Inflammatory Effects of 1 *in vivo*

**FIGURE 4:**
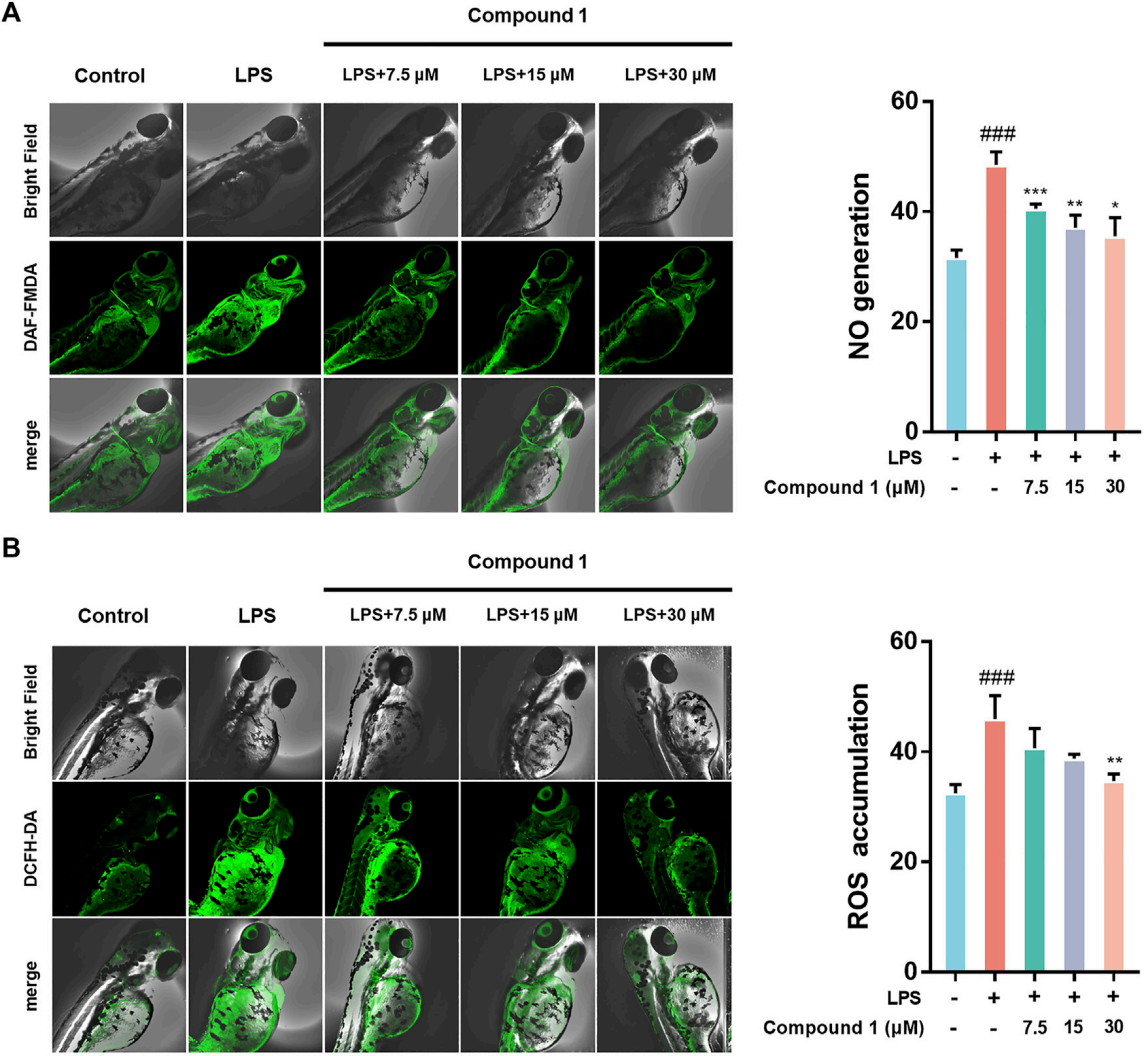
**(A) 1** inhibited the generation of NO in LPS-stimulated zebrafish. **(B) 1** inhibited the accumulation of ROS in LPS-stimulated zebrafish. Zebrafish were stimulated with LPS (10 μg/ml) and then treated with **1** or DMSO. Fluorescence intensity was quantified using ImageJ. Data are expressed as the mean ± SD. ###*p* < 0.001, compared with the normal control group. *p < 0.05, **p < 0.01, and ***p < 0.001, compared with the LPS group.

Zebrafish are promising animal models for the bioactivity and safety evaluation of trace natural products (Lin et al., 2021). The promising anti-inflammatory effects of compound **1** in LPS-induced RAW264.7 macrophages including the ability to inhibit release of NO and intracellular ROS accumulation, which aroused our concern to investigate the *in vivo* anti-inflammatory activity using a zebrafish model. The results indicate that **1** can effectively inhibit NO generation as well as ROS accumulation in LPS-induced zebrafish embryos in a concentration-dependent manner (Figure 4).

### Investigation of the Potential Mechanisms of Compound 1 by RNA-Seq

**FIGURE 5:**
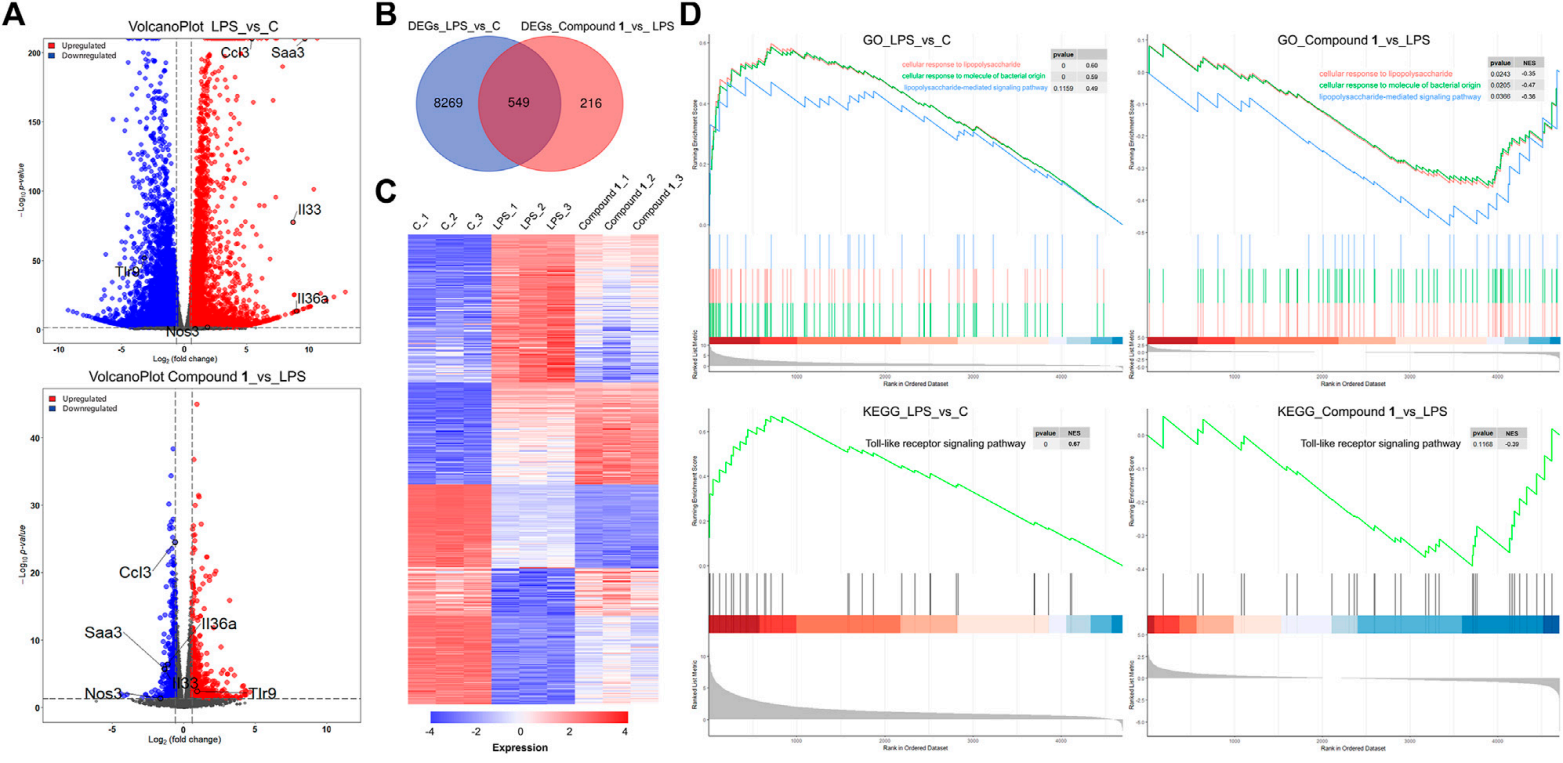
**(A)** A total of 4664 DEGs were upregulated and 4154 DEGs were downregulated in the LPS group versus the CK group, as presented by a volcano plot. A total of 371 DEGs were upregulated and 394 DEGs were downregulated in the treatment group versus the LPS group, as presented by a volcano plot, involving the mRNA expression of genes mediating inflammation reversal. **(B)** Venn diagrams of overlapping genes with DEGs from the LPS group versus the CK group and treatment group versus the LPS group. **(C)** Heatmap of 549 common DEGs screened from the Venn diagram intersection. **(D)** The gene set contains 7885 genes closely associated with inflammation and GO analysis and KEGG analysis based on gene set enrichment analysis (GSEA).

To further explore the molecular mechanisms underpinning the anti-inflammatory activity of **1**, RNA-Seq was used to generate clues about the inflammation-related signaling pathway in an unbiased manner (Huang et al., 2021). In the DESeq2 step, the cut-offs of adjusted *p* value < 0.05 and absolute fold change ≥1.5 were applied to select differentially expressed genes (DEGs). Compared to the control check (CK) group, 4664 upregulated DEGs and 4154 downregulated DEGs were observed in the volcano plot in the LPS group. A total of 371 DEGs were upregulated and 394 DEGs were downregulated in the treatment group versus the LPS group, as presented in the volcano plot, involving the mRNA expression of genes mediating inflammation reversal (Figure 5A). For example, *nos3*, which regulates NO production; *ccl3*, which encodes macrophage inflammatory protein 1α; *IL36a* and *IL33*, which are involved the activation of NF-κB and MAPK signaling pathways. Venn diagram and heat map demonstrate the reversal effect of treatment with compound **1**. To investigate the potential inflammation-related mechanism of **1**, K-means clustering was adopted to assign genes to 2 clusters (A and B) (Ge et al., 2018). After GO analysis, cluster B, which contains 7885 genes, was thought to be closely associated with inflammation (Supplementary Figure S13), and GO analysis and KEGG analysis based on gene set enrichment analysis (GSEA) were performed (Subramanian et al., 2005; Huang et al., 2022). The results of GO_GSEA indicated that 1 could reverse the LPS-induced upregulation of three biological processes, including cellular response to molecules of bacterial origin, cellular response to lipopolysaccharide, and lipopolysaccharide-mediated signaling pathway. The results of KEGG GSEA suggested that the Toll-like receptor signaling pathway related to the anti-inflammatory response was significantly enhanced by LPS and significantly reversed by **1** (Figure 5D). As a membrane receptor for LPS, the TLR4-MD-2 heterodimer can trigger the activation of NF-κB) and MAPK signaling pathways and the subsequent secretion of many proinflammatory cytokines and chemokines. Therefore, the potential mechanism of the anti-inflammatory effect of **1** may involve the MAPK and NF-κB signaling pathways.

### Compound 1 Blocked the NF-κB Pathways and Suppressed the Translocation of NF-κB/p65

**FIGURE 6:**
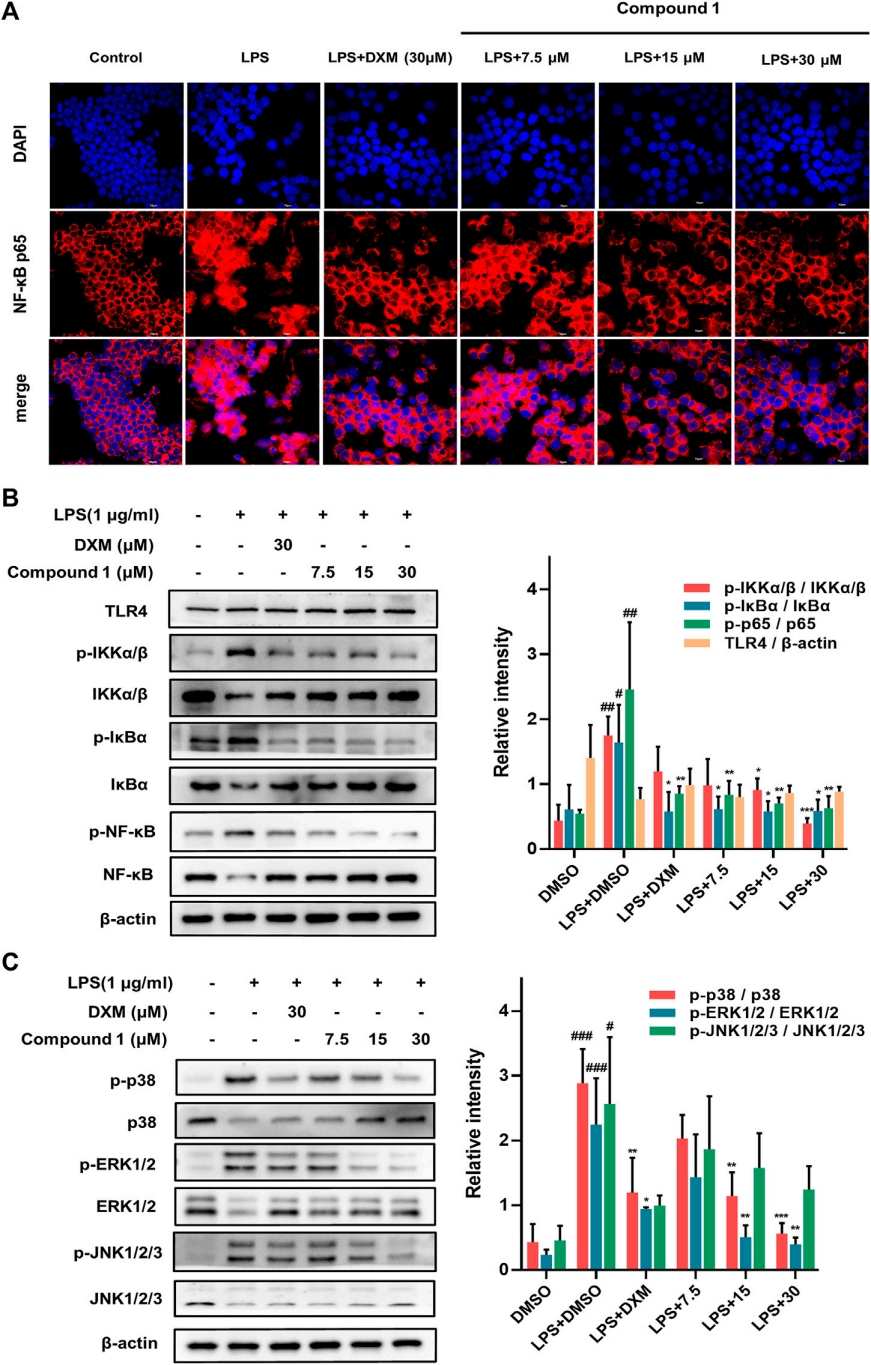
**(A)** The protective effect of **1** in LPS-treated RAW264.7 macrophages. Representative red fluorescence images indicating intracellular p65 stained by Cy3. RAW264.7 macrophages were pretreated with **1** for 1 h and then exposed to 1 μg/ml LPS for 2 h **(B,C)** RAW264.7 macrophages were pretreated with **1** for 1 h and exposed to 1 μg/ml LPS. Expression of IKK, p-IKK (LPS stimulated 1 h), NF-κB/p65, p-NF-κB/p65 (LPS stimulated 2 h), IκBα and p-IκBα (LPS stimulated 2 h) in the NF-kB pathway. Expressions of ERK, p-ERK (LPS stimulated 1 h), JNK, p-JNK (LPS stimulated 1 h), p38 and p-p38 (LPS stimulated 2 h) in the MAPK pathway. The levels of signaling pathway proteins were determined by Western blotting. Data are expressed as the mean ± SD. ###*p* < 0.001, compared with the normal control group. *p < 0.05, **p < 0.01, and ***p < 0.001, compared with the LPS group.

Activation of the NF-κB transcription factor is critically involved in executing inflammatory and immune reactions (Hochrainer et al., 2015). IκBα retains NF-κB in the cytoplasm while preventing its translocation to the nucleus. When the NF-κB pathway is activated, phosphorylated IκBα becomes polyubiquitinated and degraded, and activated NF-κB is then transferred to the nucleus to participate in transcriptional processes (Baker et al., 2011). Compared to LPS-induced RAW264.7 macrophages, **1** effectively suppressed the nuclear translocation of NF-κB/p65 (with red fluorescence) in a concentration-dependent manner (Figure 6A). Furthermore, Western blotting was employed to detect key upstream proteins regulating NF-kB nuclear translocation, including NF-κB, IκB-α and IKKα/β (Figure 6B). The results revealed that **1** could decrease the ratio of phosphorylated proteins to total proteins mentioned above and hence block the NF-κB pathways.

### Compound 1 Suppressed the MAPK Pathways

The MAPK pathways mediated by the ERK, JNK and p38 MAPK family members play a pivotal role in inflammatory stimulus responses (Kim et al., 2008). The results of Western blotting also indicated that **1** can block the phosphorylation of JNK, ERK and p38 MAPK, and the total protein levels of JNK, ERK and p38 MAPK showed an inverse change (Figure 5C), which suggests that the inhibition of the MAPK signaling pathway may also play a nonnegligible role in the anti-inflammatory effect of **1**.

### Compounds 1-4 Demonstrate Affinity for the TLR4 Protein

**FIGURE 7:**
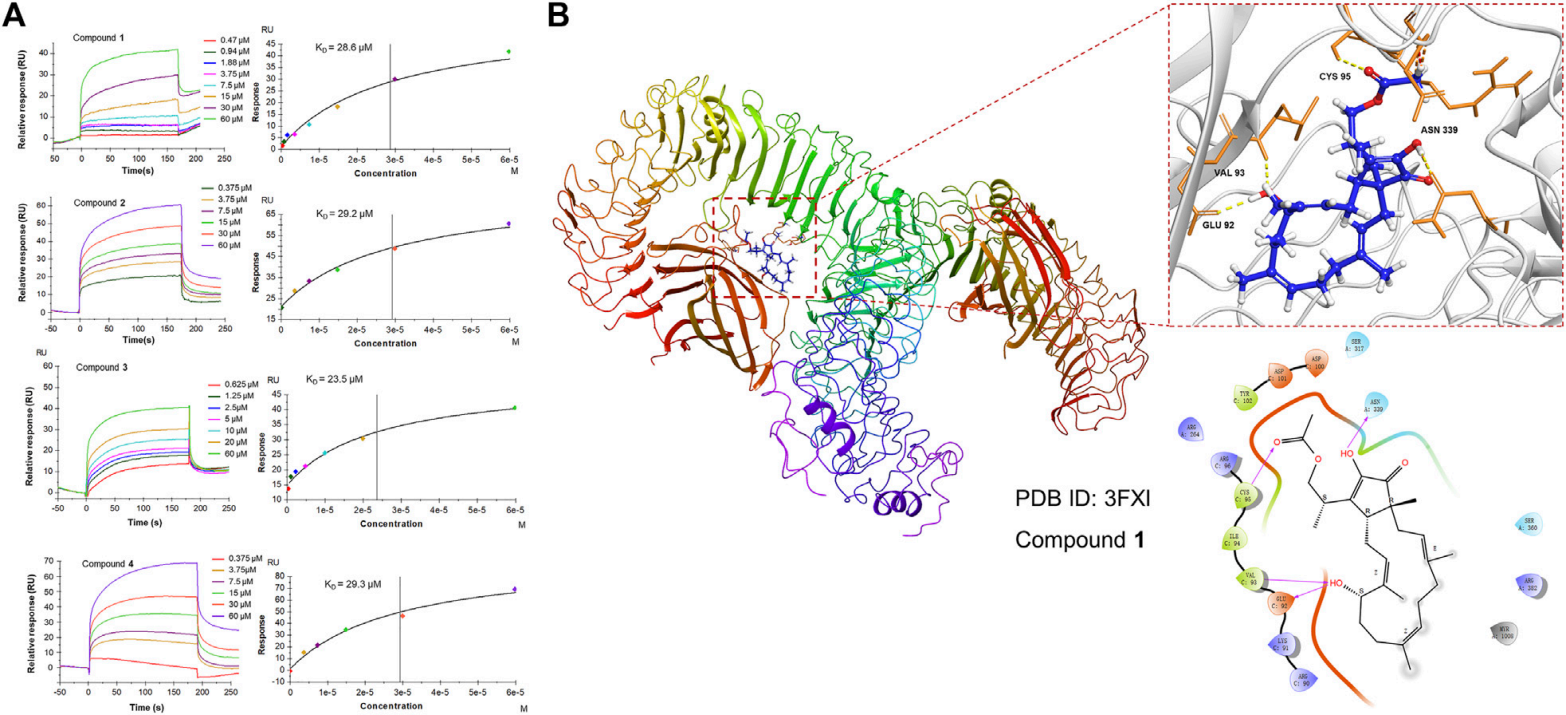
**(A)** SPR analysis of the binding affinity of **1**-**4** to the TLR4 protein. Apparent equilibrium dissociation constants (KD) were then calculated by global fitting using a steady-state affinity model in Biacore T200 evaluation software. The KD values of **1**-**4** were 28.6, 29.2, 23.5, and 29.3 μM, respectively. **(B)** Structures and orthogonal views of the pocket of binding between **1** and TLR4-MD-2 (PDB ID: 3FXI). Molecular docking simulations were obtained at the lowest energy conformation. Hydrogen bonding interactions are shown by dashes.

Since **1** can effectively inhibit inflammatory responses triggered by the NF-κB and MAPK pathways, which are downstream of TLR4, a subsequent SPR assay was performed to further assess the direct interaction of **1-4** with TLR4 *in vitro* to provide a possible interpretation for the inhibition of the downstream signaling pathways. The results demonstrated that **1**-**4** presented affinity for the TLR4 protein with KD values of 28.6, 29.2, 23.5, and 29.3 μM, respectively (Figure 7A), which may modify the conformation of the TLR4-MD-2 complex and subsequently obstruct the interaction between LPS and the TLR4-MD-2 heterodimer. Further molecular docking analysis also revealed that hydrogen bonds were formed between **1** and four amino acid residues at the TLR4-MD-2 active site, including GLU 92, VAL 93, CYS 95 and ASN 339 (Figure 7B).

## Discussion

Immune cells and mediators are involved in the inflammatory response induced by bacterial invasion, which is essential for survival (Medzhitov, 2021). As monocyte-derived innate immune cells, macrophages are extensively involved in inflammation and regulate tissue homeostasis and adaptive responses (Yu et al., 2020). In response to bacteria and LPS, macrophages undergo proinflammatory differentiation, characterized by the release of large amounts of cytokines such as TNF-α, IL-1β, and IL-6, as well as ROS (Reuter et al., 2010; Chovatiya and Medzhitov, 2014). These processes are essential for the organism’s immune defense and killing of microorganisms. However, excessive inflammatory activation in macrophages may lead to collateral tissue damage. Hence, inflammation is inextricably associated with pathology.

As a receptor for LPS, the TLR4/MD-2 complex plays an important role in inflammation initiated by bacterial invasion. Upon recognition, the TLR4/MD-2 complex dimerizes and activates downstream NF-κB and MAPK signaling pathways, which are frequently upregulated or overactivated in inflammatory disorders (Baker et al., 2011). The seminal event in NF-κB activation is the phosphorylation of IκB, which is mediated by the IKK complex (Suzuki and Verma, 2008). In addition, TLR4 uses distinct MAP3Ks to activate different MAPK effectors (JNK, p38, and ERK) (Banerjee et al., 2008). Hence, small molecules suppressing TLR4-related signaling pathways may be an effective strategy for the treatment of excessive inflammation. To date, some TLR4 specific inhibitors have been identified (e.g., eritoran, naloxone, TAK242) (Takashima et al., 2009; Barochia et al., 2011; Lewis et al., 2012), but are limited by toxicity, selectivity, and other disadvantages. Among these, a highly anticipated small molecule inhibitor of TLR4, TAK242, was declared failure in Phase III clinical trial, evoked an urgent need for a new, viable solution (Rice et al., 2010).

Sesterterpenoids are highly rewarding sources of hit compounds with anti-inflammatory activity. In this study, anti-inflammatory assays indicated that fusaproliferin (**1**) and its analogs (**2**-**4**) showed obvious anti-inflammatory activity by attenuating the secretion of proinflammatory mediators, including NO, ROS, IL-6, TNF-α, and IL-1β, as well as iNOS and COX-2 *in vitro* and *in vivo*. Transcriptome analysis is an effective way to provide valuable clues about the mechanism, which may provide a more practical guide to drug efficacy, drug toxicity assessment, and drug combination therapy. Transcriptome analyses on the basis of RNA-seq indicated that **1** could reverse the LPS-induced changes in the gene signature involved in the cellular response to molecules of bacterial origin, and lipopolysaccharide-mediated signaling pathways such as toll-like receptor 4 (TLR4) related signaling pathways. As a membrane receptor for LPS, the TLR4-MD-2 heterodimer can trigger the activation of nuclear factor kappa-B (NF-κB) and mitogen-activated protein kinase (MAPK) signaling pathways. Our western blotting results revealed that **1** inhibited the phosphorylation of IκB, degradation of IκBα, and phosphorylation of NF-κB and reduced the transportation of NF-κB. **1** also decreased the phosphorylation of MAPKs, including p38, ERK, and JNK. SPR assays indicated that **1**-**4** exhibited affinity for the TLR4 protein with KD values of 23.5–29.3 μM. Notably, molecular docking analysis indicated that several hydrogen bonds were formed by **1** with four amino acid residues at the active site of the interaction between LPS and the TLR4-MD-2 heterodimer. This obstruction might be a possible interpretation of the deregulation of TLR4-related signaling pathways. Meanwhile, the interaction between LPS, fusaproliferin and TLR4-MD-2 complexes is complex, which needs to be further elucidated by more direct evidence (Yuan et al., 2019; Wu et al., 2022). Although we have demonstrated that fusaproliferin does not show significant toxicity to zebrafish embryos at experimental concentrations, its safety and efficacy need to be evaluated in more *in vivo* animal models.

## Conclusion

The anti-inflammatory activity of fusaproliferin and its analogs (**1–4**) was confirmed by attenuating the production of ROS and NO (*in vitro* and *in vivo*), the secretion of proinflammatory cytokines, including TNF-α, IL-6, and IL-1β (*in vitro*) and the expression of iNOS and COX-2 (*in vitro*) with better or comparable activities than those of the positive control, dexamethasone. Fusaproliferin inhibited the phosphorylation of essential proteins in the TLR4 downstream NF-κB and MAPK signaling pathways, which might account for the anti-inflammatory activity of fusaproliferin. Furthermore, fusaproliferin demonstrates affinity for the active site of TLR4, which may modify the conformation of the TLR4-MD-2 complex and subsequently obstruct the interaction between LPS and the TLR4-MD-2 heterodimer. These results suggest that fusaproliferin can be considered a promising prototype (for instance, a hit compound of TLR-4 inhibitor) for the treatment of inflammation-related diseases.

## Data Availability

The datasets presented in this study can be found in online repositories. The names of the repository/repositories and accession number(s) can be found below: https://www.ncbi.nlm.nih.gov/, PRJNA788753.
