# Supplementary material for: Investigation of the Anti-Inflammatory Activity of Fusaproliferin Analogues Guided by Transcriptome Analysis
Source: Front Pharmacol. 2022 May 5;13:881182. doi: 10.3389/fphar.2022.881182 (PMC10136769; doi:10.3389/fphar.2022.881182)
Supplement: Supplementary file 1 [file DataSheet1.PDF]

Supplementary Material

Investigation of the Anti-inflammatiory Activity of Fusaproliferin Analogues Guided by Transcriptome Analysis

Qi-Xuan Kuang [1], Li-Rong Lei [1], Qing-Zhou Li [1], Wan Peng [3], Yu-Mei Wang [2], Yi-Fei Dai [4], Dong Wang [2], Yun Deng [1*] and Da-Le Guo [1*]

[1] State Key Laboratory of Southwestern Chinese Medicine Resources, School of Pharmacy, Chengdu University of Traditional Chinese Medicine, Chengdu 611137, People's Republic of China

[2] School of Basic Medical Sciences, Chengdu University of Traditional Chinese Medicine, Chengdu 611137, People's Republic of China

[3] Institute of Rare Diseases, West China Hospital of Sichuan University, Chengdu, 610065, People's Republic of China

[4] Department of Basic Medical Sciences, School of Medicine, Tsinghua University, Beijing, 100084, People's Republic of China

*** Correspondence:**
Corresponding Author
dengyun@cdutcm.edu.cn (Yun Deng); guodale@cdutcm.edu.cn (Da-Le Guo)

1 Supplementary Data

1.1 sequence read archive (SRA) accession numbers of RNA-seq

Group	Accession number	Group	Accession number	Group	Accession number
Compound_1_1	SAMN24425690	LPS_1	SAMN24425693	C_1	SAMN24425696
Compound_1_2	SAMN24425691	LPS_2	SAMN24425694	C_2	SAMN24425697
Compound_1_3	SAMN24425692	LPS_3	SAMN24425695	C_3	SAMN24425698

2 Supplementary Figures and Tables

2.1 The ^{1}H-NMR spectrum of 1

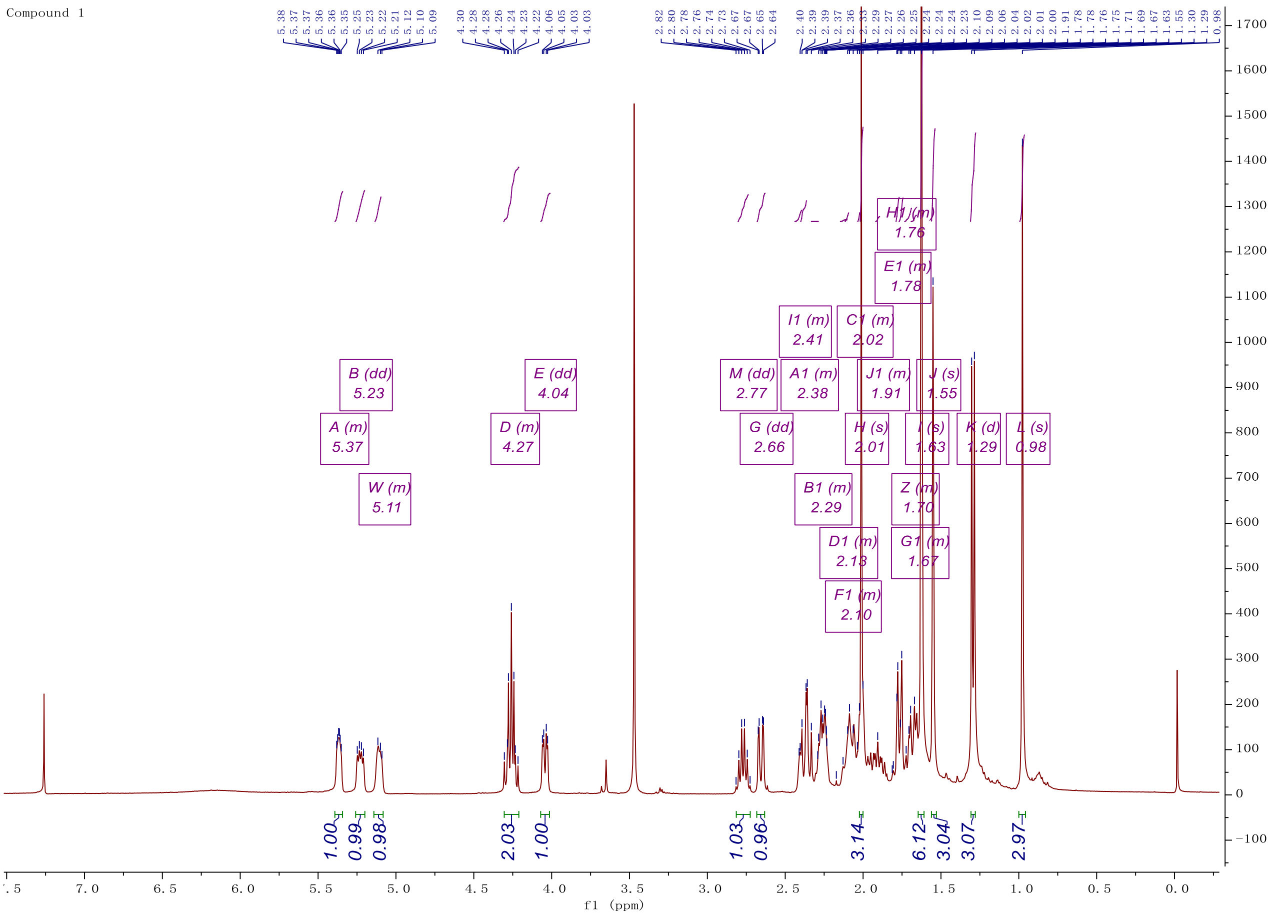

Supplementary Figure 1. ^{1}H-NMR spectrum (400 MHz) of **1** measured in $CDCl_3$ with a Bruker 400 Ascend NMR instrument.

2.2 The ^{13}C-NMR spectrum of 1

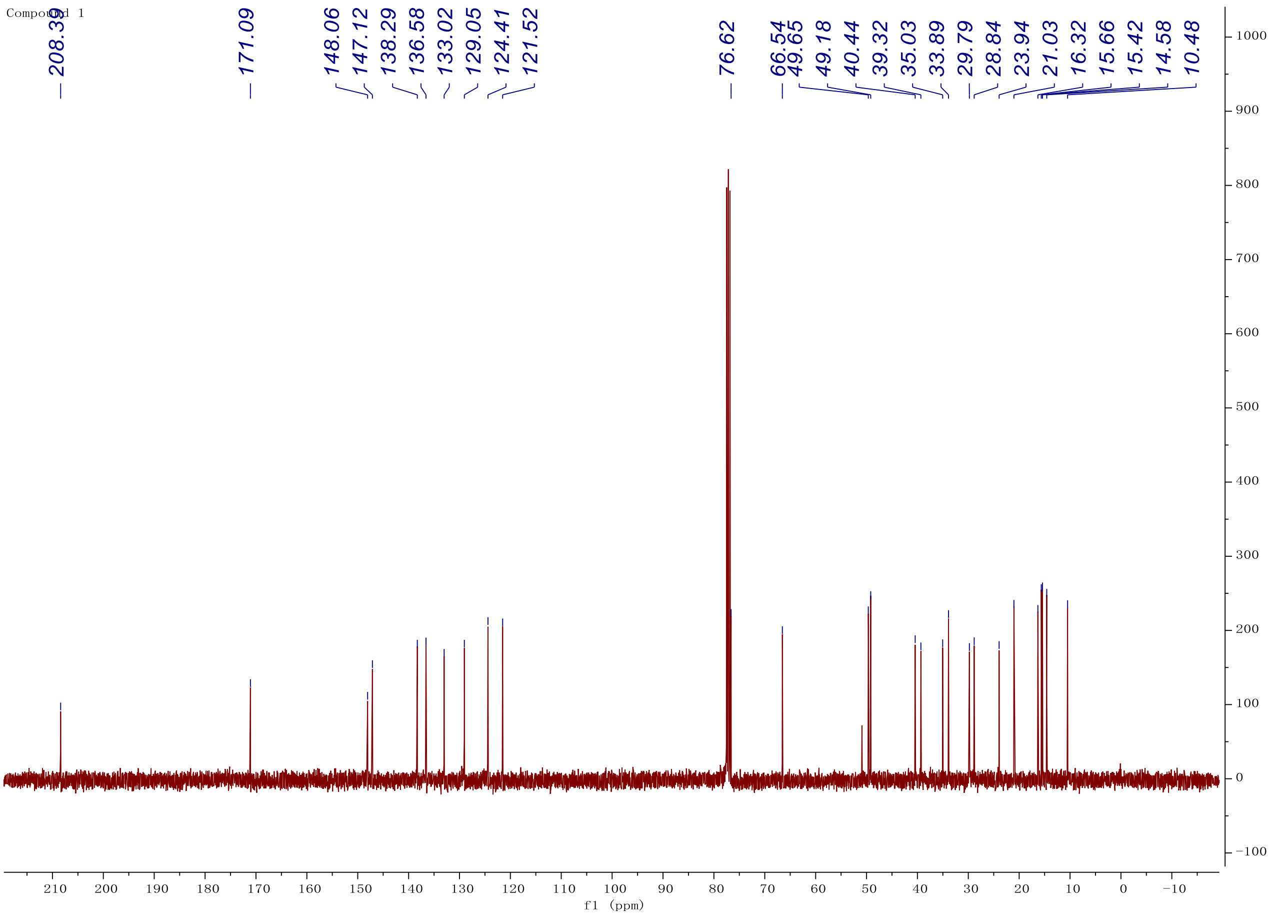

Supplementary Figure 2. ^{13}C-NMR spectrum (100 MHz) of **1** measured in $CDCl_3$ with a Bruker 400 Ascend NMR instrument.

2.3 The ^{1}H-NMR spectrum of 2

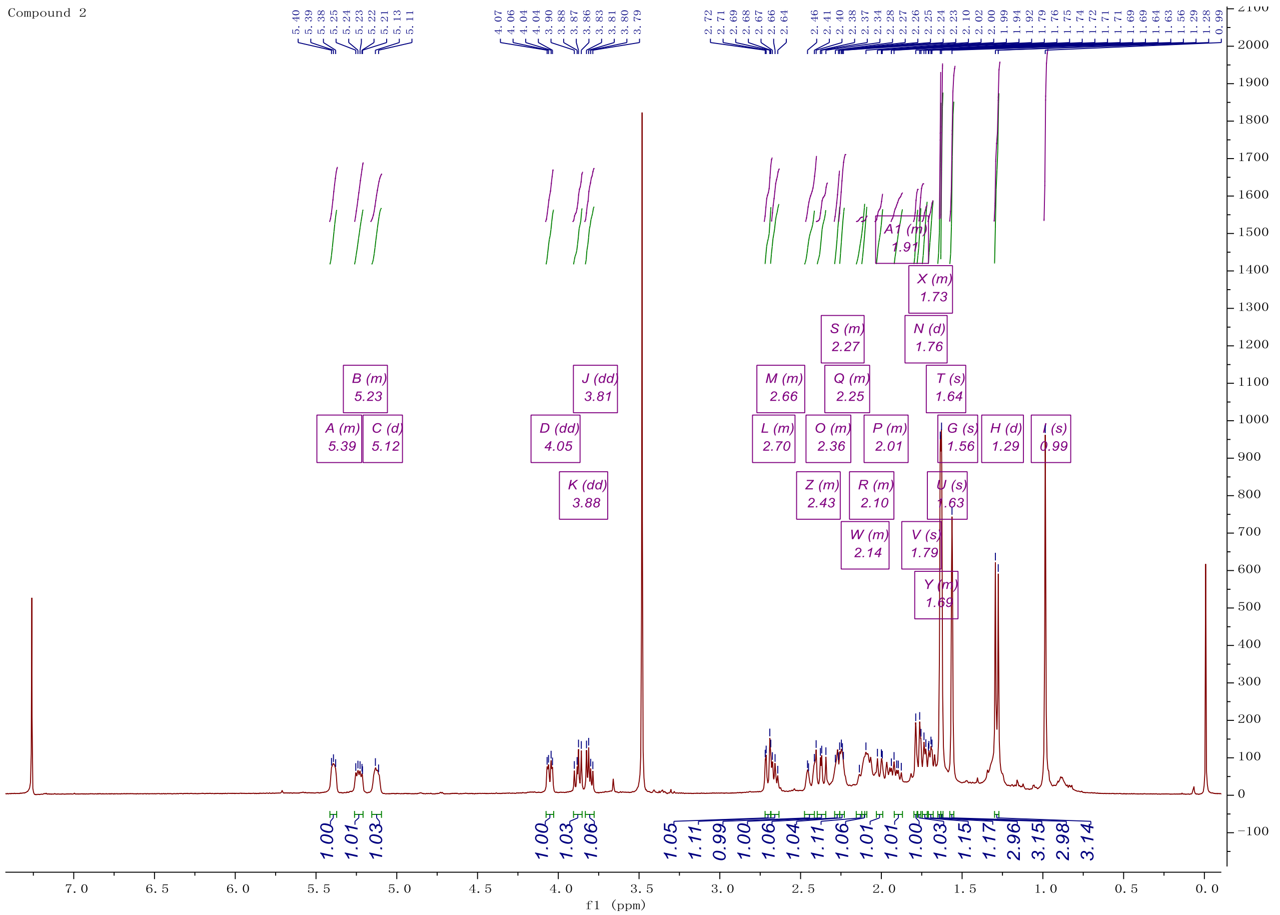

Supplementary Figure 3. ^{1}H-NMR spectrum (400 MHz) of **2** measured in $CDCl_3$ with a Bruker 400 Ascend NMR instrument.

2.4 The ^{13}C-NMR spectrum of 2

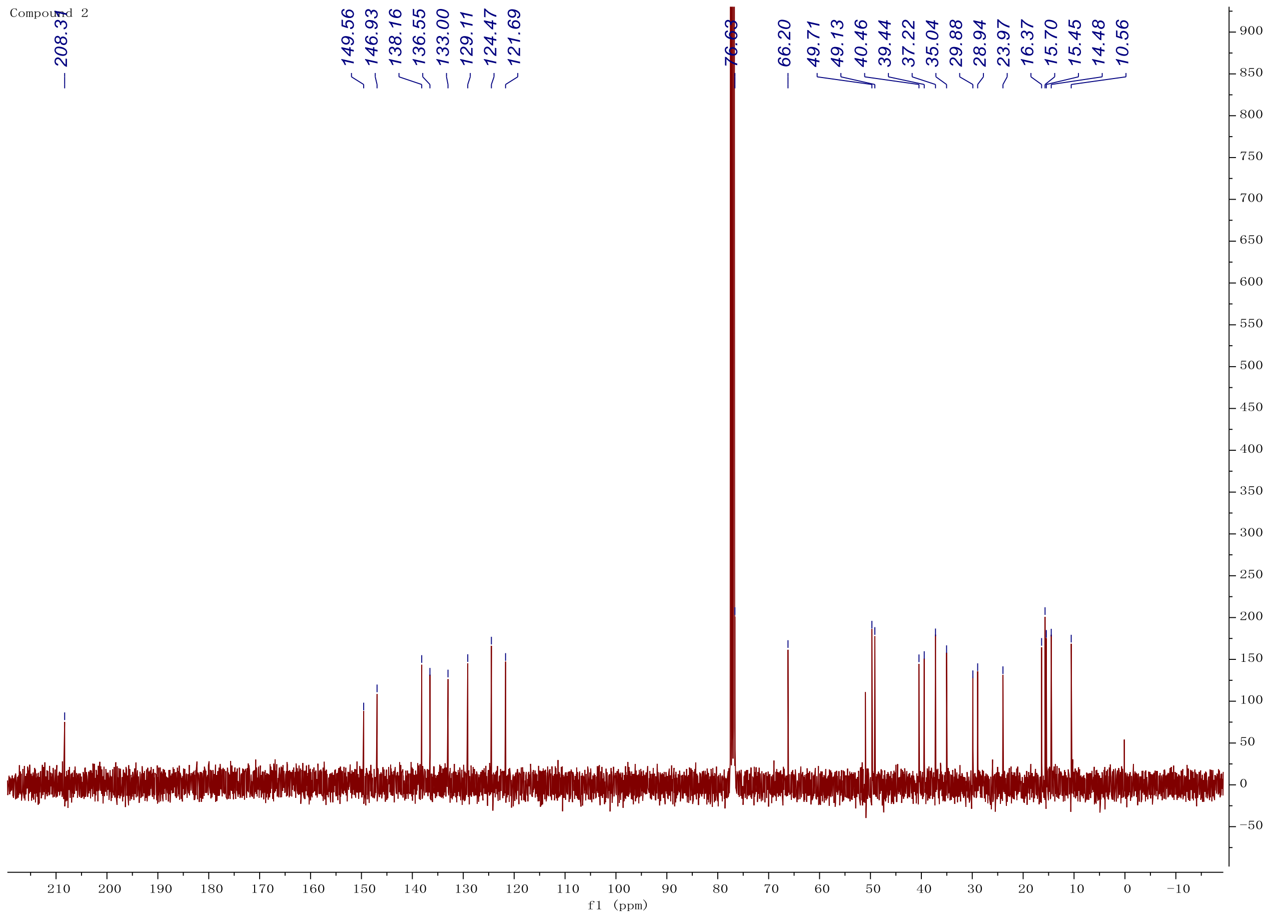

Supplementary Figure 4. ^{13}C-NMR spectrum (100 MHz) of **2** measured in $CDCl_3$ with a Bruker 400 Ascend NMR instrument.

2.5 The ^{1}H-NMR spectrum of 3

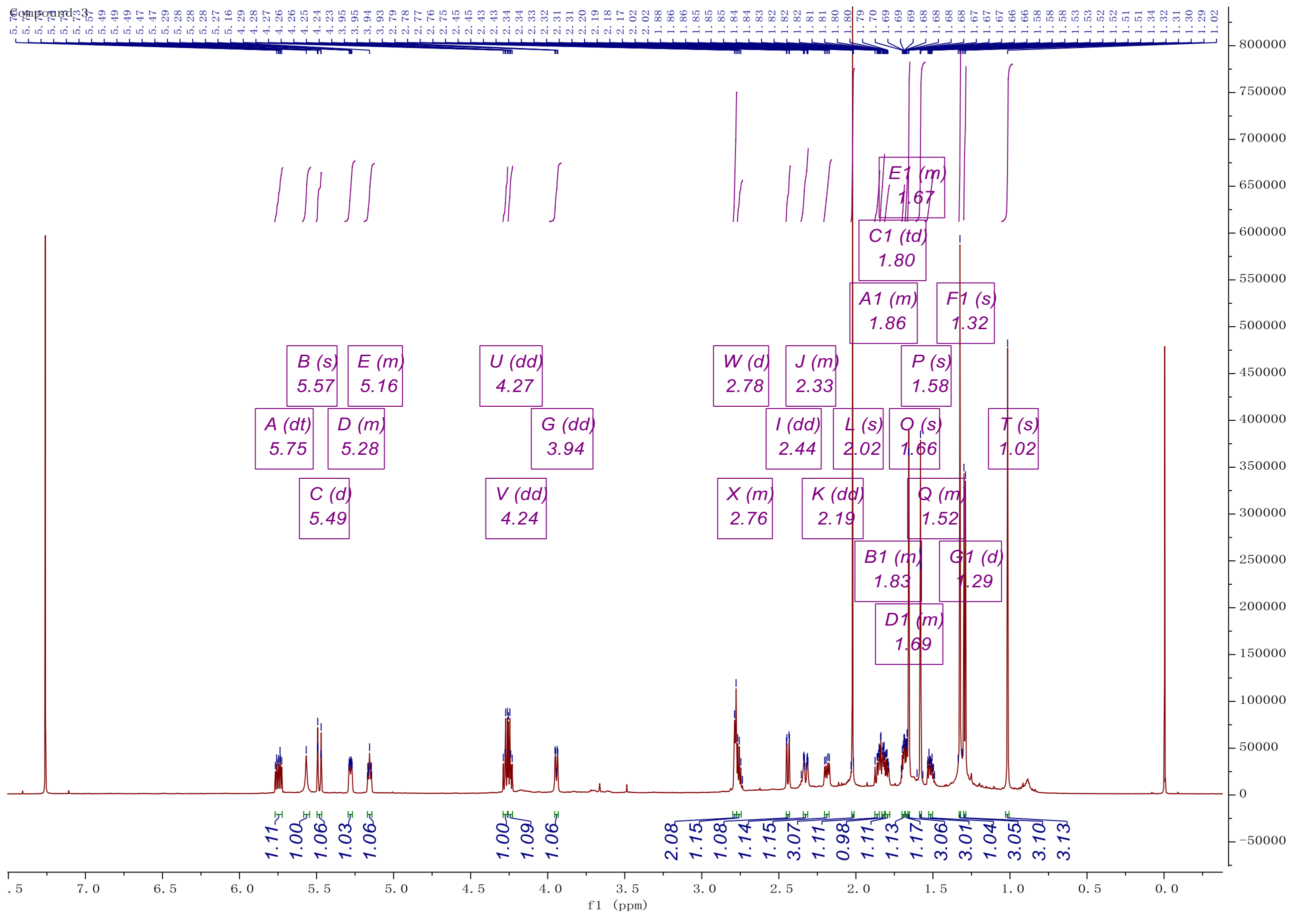

Supplementary Figure 5. ^{1}H-NMR spectrum (400 MHz) of **3** measured in $CDCl_3$ with a Bruker 400 Ascend NMR instrument.

2.6 The ^{13}C-NMR spectrum of 3

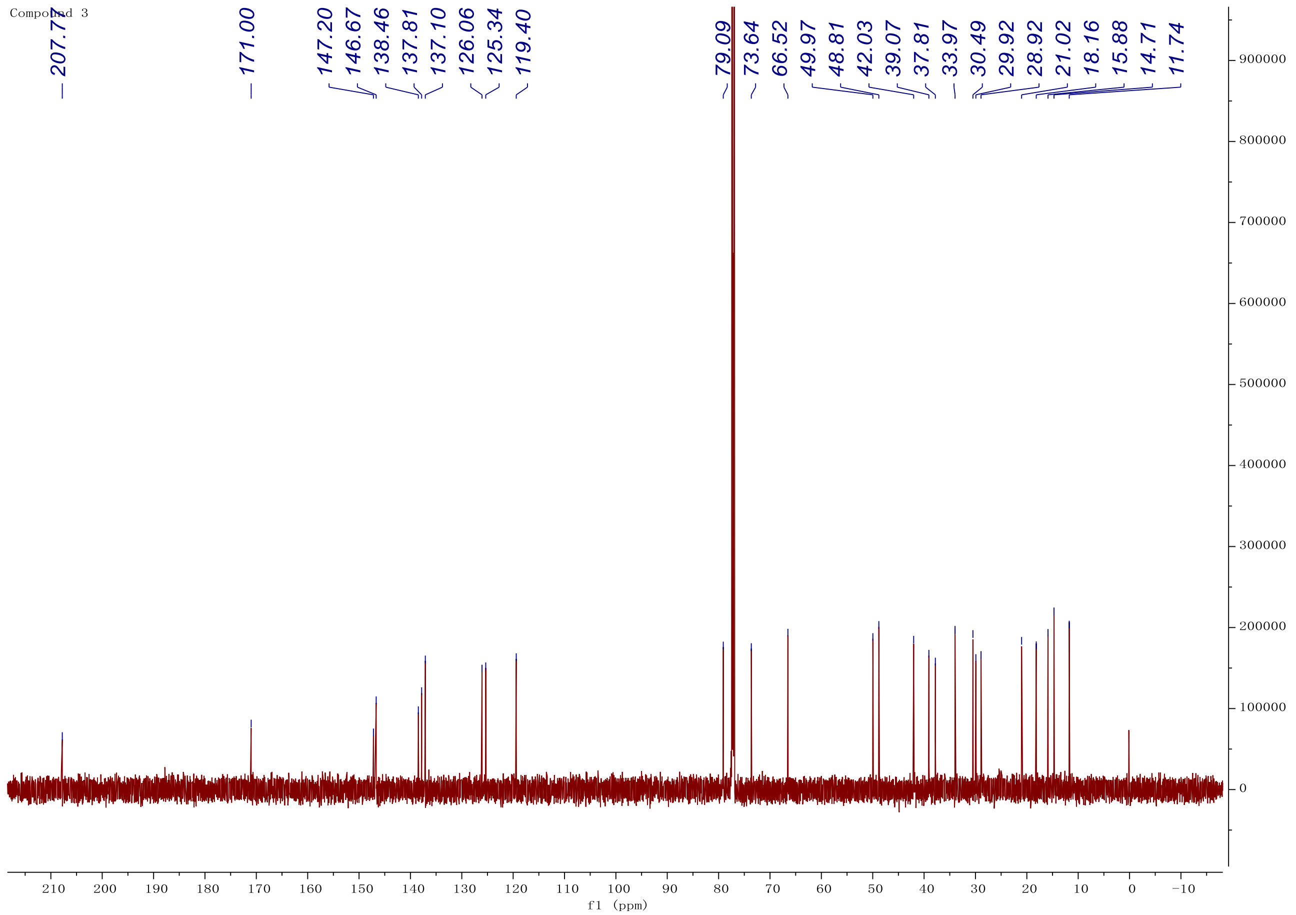

Supplementary Figure 6. ^{13}C-NMR spectrum (175 MHz) of **3** measured in $CDCl_3$ with a Bruker 700 Ascend NMR instrument.

2.7 The ^{1}H-NMR spectrum of 4

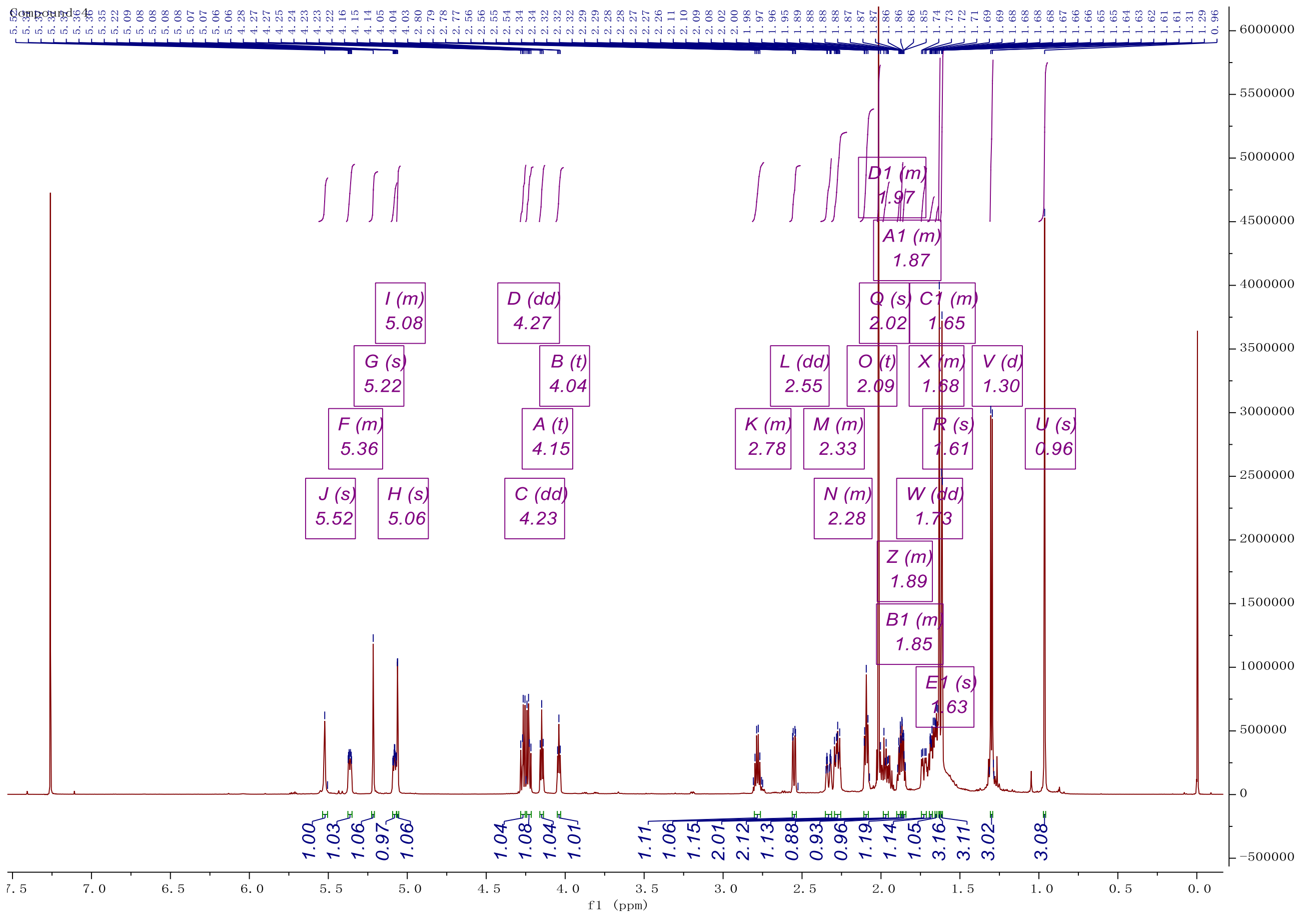

Supplementary Figure 7. ^{1}H-NMR spectrum (700 MHz) of **4** measured in $CDCl_3$ with a Bruker 700 Ascend NMR instrument.

2.8 The ^{13}C-NMR spectrum of 4

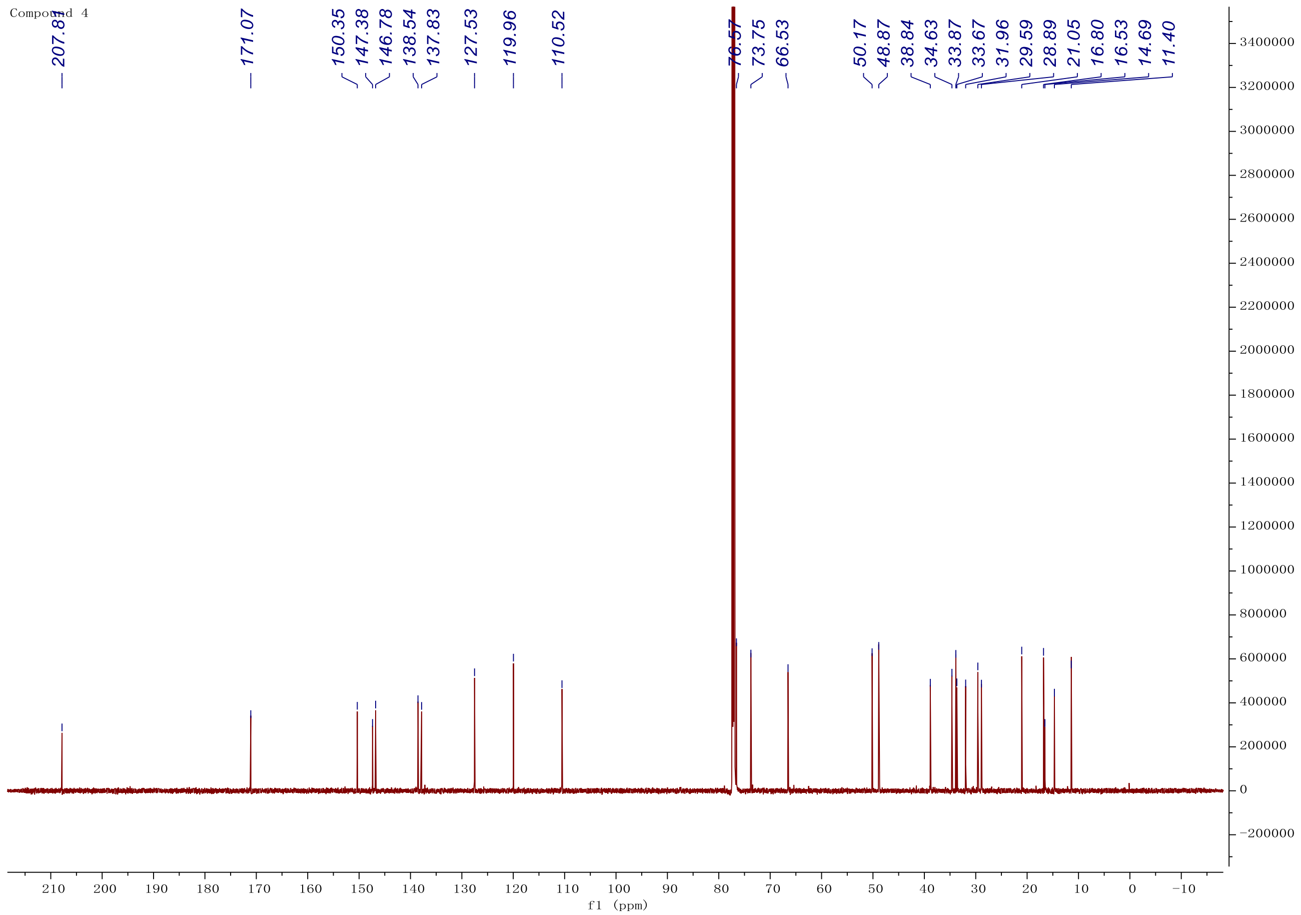

Supplementary Figure 8. ^{13}C-NMR spectrum (175 MHz) of **4** measured in $CDCl_3$ with a Bruker 700 Ascend NMR instrument.

2.9 ^{1}H-^{1}H COSY spectrum of 4

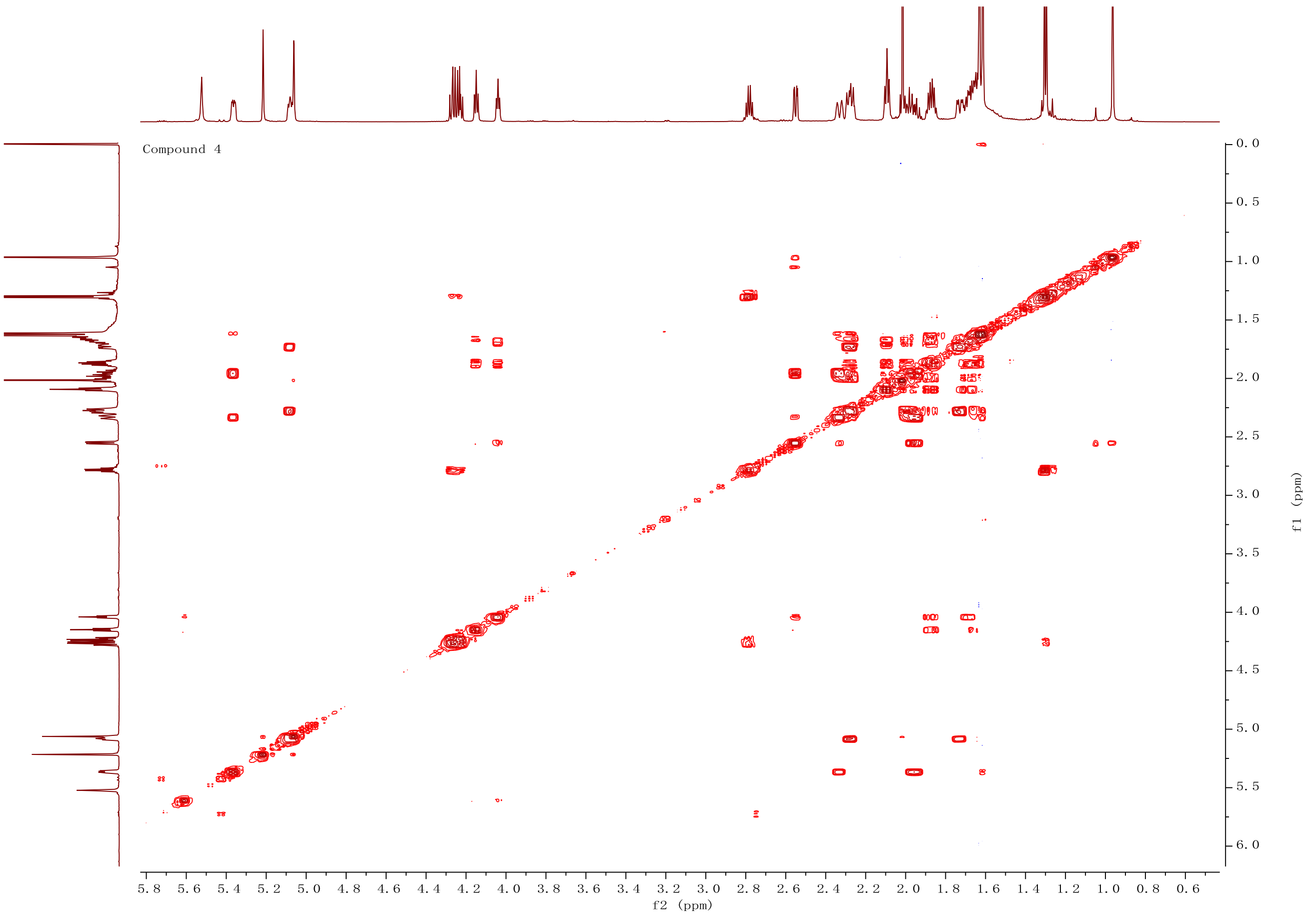

Supplementary Figure 9. ^{1}H-^{1}H COSY spectrum of **4** measured in $CDCl_3$ with a Bruker 700 Ascend NMR instrument.

2.10 HSQC spectrum of 4

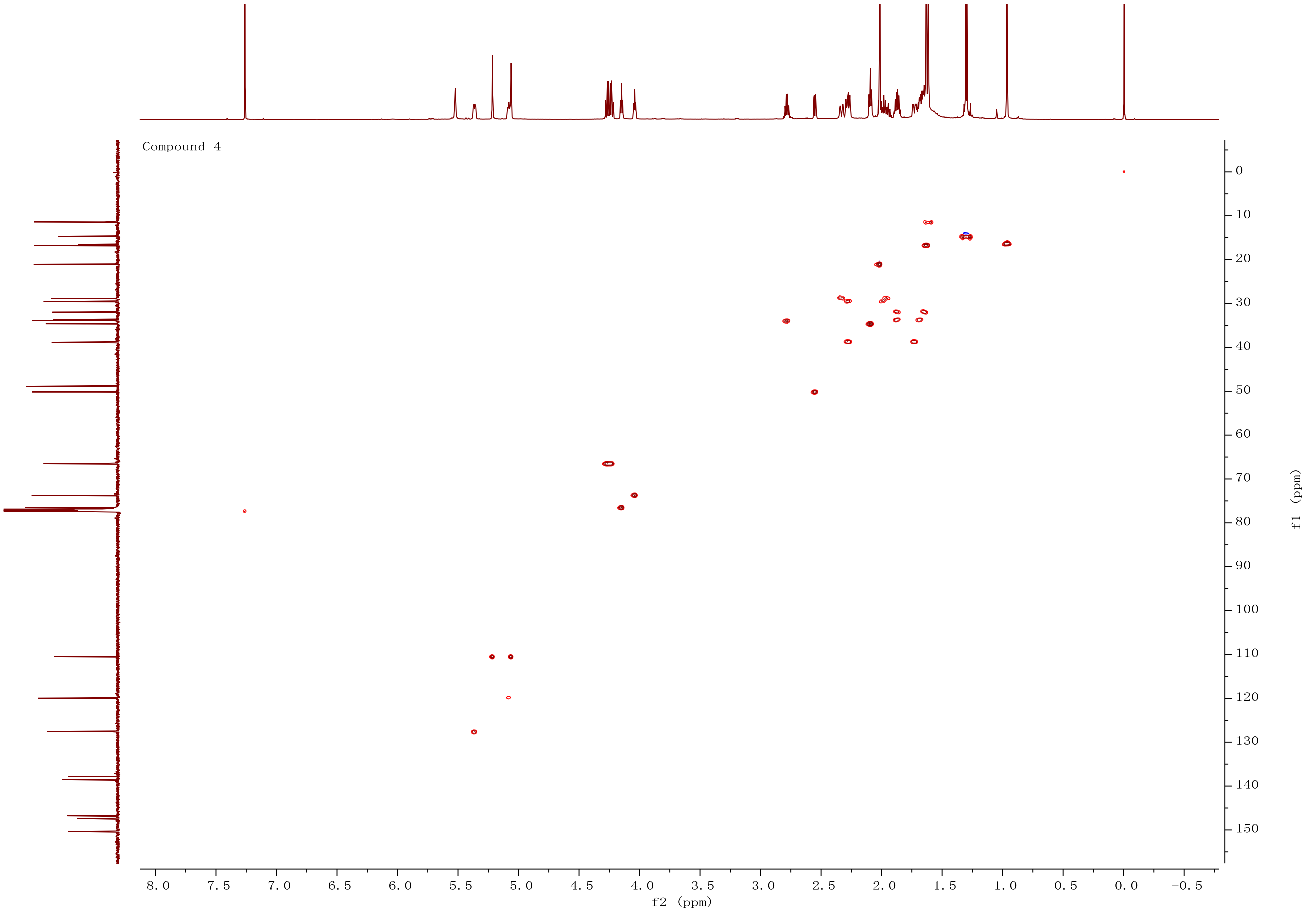

Supplementary Figure 10. HSQC spectrum of **4** measured in $CDCl_3$ with a Bruker 700 Ascend NMR instrument.

2.11 HMBC spectrum of 4

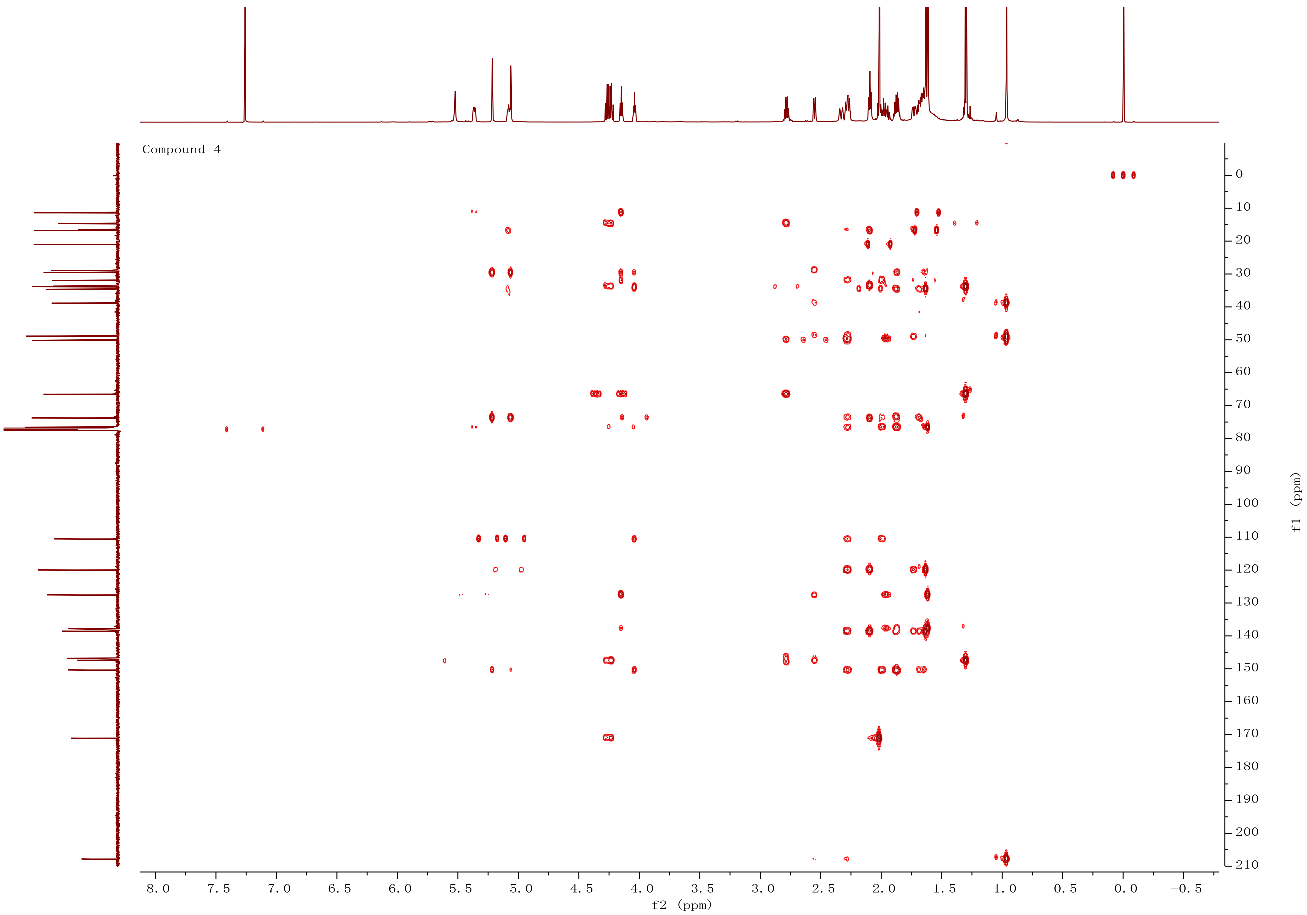

Supplementary Figure 11. HMBC spectrum of **4** measured in $CDCl_3$ with a Bruker 700 Ascend NMR instrument.

2.12 NOESY spectrum of 4

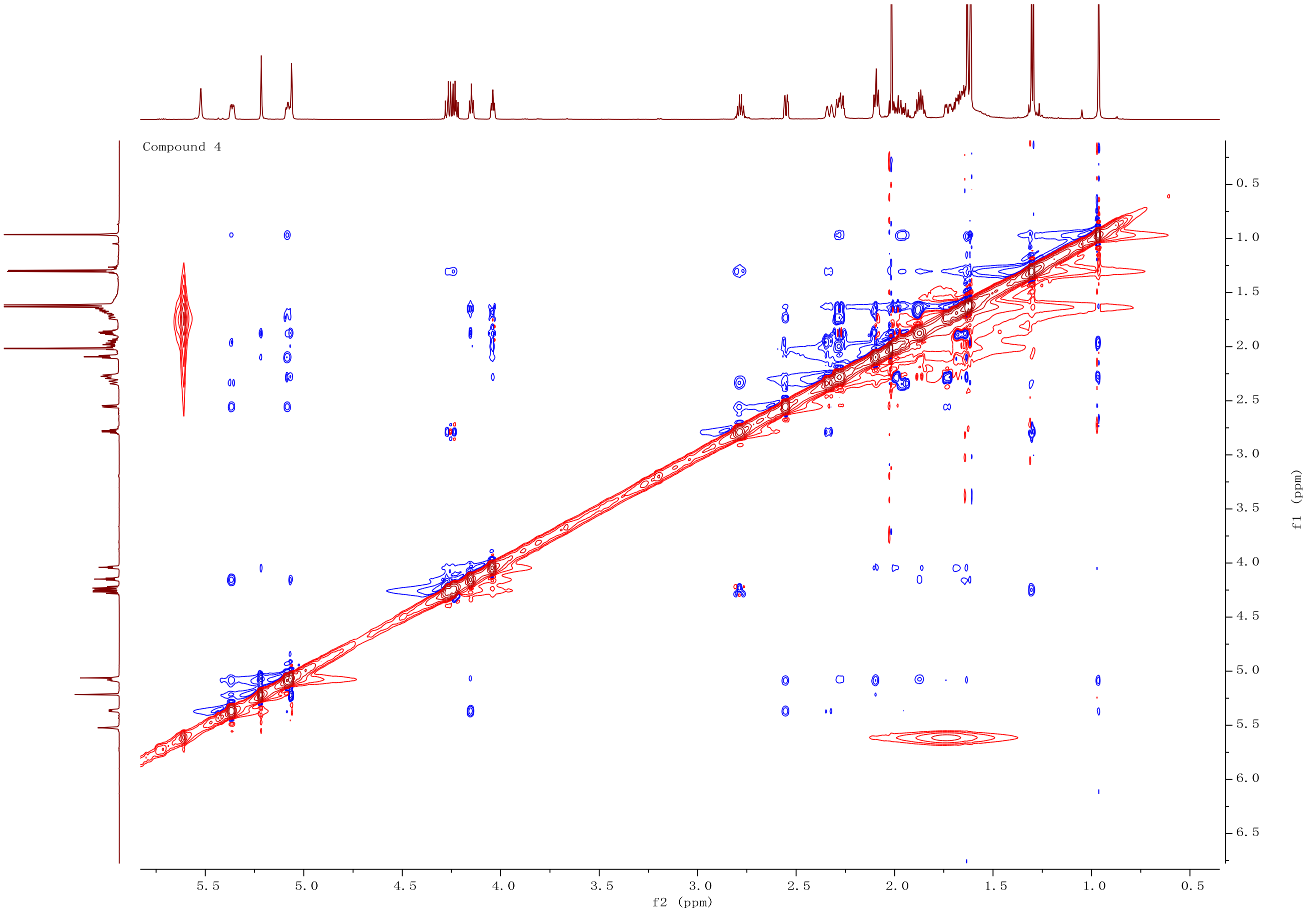

Supplementary Figure 12. NOESY spectrum of **4** measured in $CDCl_3$ with a Bruker 700 Ascend NMR instrument.

2.13 Table S1 ^{1}H NMR and ^{13}C NMR data of compounds 1-4

Table 1 ^{1}H NMR and ^{13}C NMR data of compounds **1-4**

Position	1[a]		2[a]		3[b]		4[b]	
	δ_H (*J* in Hz)	δ_C	δ_H (*J* in Hz)	δ_C	δ_H (*J* in Hz)	δ_C	δ_H (*J* in Hz)	δ_C
1	-	49.2, qC		49.1, qC	-	48.8, qC	-	48.9, qC
2	1.70, m	39.3, CH_2	1.76, m	39.4, CH_2	2.19, m	39.0, CH_2	2.28, m	38.8, CH_2
	2.38, m		2.36, m		1.83, m		1.73, m	
3	5.23, dd (10.6, 5.4)	121.5, CH	5.23, m	121.7, CH	5.16, m	119.4, CH	5.08, m	119.9, CH

4	-	138.3, qC	-	138.2, qC	-	137.8, qC	-	138.5, qC
5	2.01, m	40.4, CH_2	2.01, m	40.5, CH_2	2.78, br. d (7.0)	42.0, CH_2	2.09, t (7.3)	34.6, CH
	2.30, m		2.25, m					
6	2.13, m	23.9, CH_2	2.10, m	23.9, CH_2	5.75, dt (15.7, 7.0)	125.3, CH	1.85, m	32.0, CH_2
	2.30, m		2.27, m				1.65, m	
7	5.11, m	124.4, CH	5.12, m	124.5, CH	5.49, d (15.5)	137.1, CH	4.04, t (5,8)	73.8, CH
8	-	133.0, qC	-	133.0, qC	-	73.6, qC	-	150.4, qC
9	1.78, m	35.0, CH_2	1.79, m	35.0, CH_2	1.80, m	37.8, CH_2	2.33, m	29.6, CH_2
	2.10, m		2.14, m		1.69, m		1.89, m	
10	1.67, m	29.8, CH_2	1.69, m	29.9, CH_2	1.52, m	29.9, CH_2	1.69, m	33.7, CH_2
	1.76, m		1.73, m		1.67, m		1.87, m	
11	4.06, dd (10.0, 3.4)	76.6, CH	4.05, dd (9.8, 3.5)	76.6, CH	3.94, dd (10.2, 2.7)	79.1, CH	4.15, t (6.5)	76.6, CH
12	-	136.6, qC	-	136.6, qC	-	138.5, qC	-	137.8, qC
13	5.37, m	129.0, CH	5.39, m	129.1, CH	5.29, m	126.1, CH	5.36, m	127.5, CH
14	1.91, m	28.8, CH_2	1.91, m	28.9, CH_2	1.86, m	28.9, CH_2	1.97, m	28.9, CH_2
	2.41, m		2.43, m		2.33, m		2.28, m	
15	2.77, dd (14.4, 7.2)	49.7, CH	2.70, m	49.7, CH	2.44, dd (11.5, 2.1)	49.9, CH	2.55, dd (10.3, 2.6)	50.2, CH
16	-	147.1, qC	-	146.9, qC	-	147.2, qC	-	146.8, qC
17	-	148.1, qC	-	149.5, qC	-	146.7, qC	-	146.8, qC
18	-	208.4, qC	-	208.3, qC	-	207.8, qC	-	207.8, qC
19	0.98, s	16.3, CH_3	0.99, s	16.4, CH_3	1.02, s	15.9, CH_3	0.96, s	16.5, CH_3
20	1.63, s	15.7, CH_3	1.64, s	15.7, CH_3	1.69, s	18.1, CH_3	1.63, s	16.8, CH_3
21	1.63, s	15.4, CH_3	1.63, s	15.5, CH_3	1.32, s	30.5, CH_3	5.06, s	110.5, CH_2
							5.22, s	

22	1.55, s	10.5, CH_3	1.56, s	10.6, CH_3	1.58, s	11.7, CH_3	1.61, s	11.4, CH_3
23	2.66, dd (11.1, 2.4)	33.9, CH	2.66, m	37.2, CH	2.76, m	33.9, CH	2.78, m	33.7, CH
24	4.28, m	66.5, CH_2	3.81, dd (10.4, 5.3)	66.2, CH_2	4.24, dd (10.6, 7.8)	66.5, CH_2	4.23, dd (10.6, 6.9)	66.5, CH_2
			3.88, dd (10.4, 6.9)		4.27, dd (10.6, 6.9)		4.27, dd (10.6, 7.9)	
25	1.29, d (7.1)	14.6, CH_3	1.29, d (7.1)	14.5, CH_3	1.29, d (7.1)	14.7, CH_3	1.30, d (7.1)	14.7, CH_3
26	-	171.1, qC			-	171.0, qC	-	171.0, qC
27	2.01, s	21.0, CH_3			2.02, s	21.0, CH_3	2.02, s	21.0, CH_3

[a.] Record by a Bruker-Ascend-400-MHz spectrometer in $CDCl_3$; [b.]Record by a Bruker-Ascend-700-MHz spectrometer in $CDCl_3$

2.14 K-means clustering

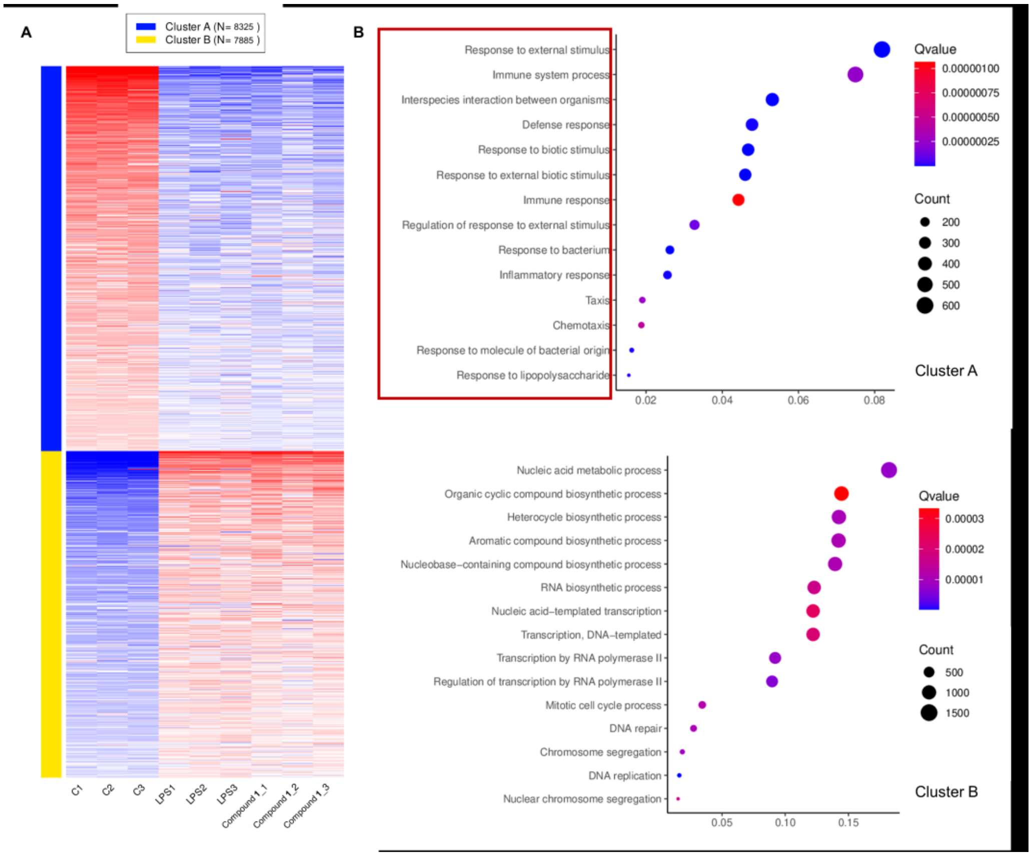

Figure S13. (**A**) K-means clustering assigns genes to 2 clusters (A and B) (**B**) GO analysis with cluster A and cluster B

2.15 Molecular docking results of **2** with TLR4-MD2

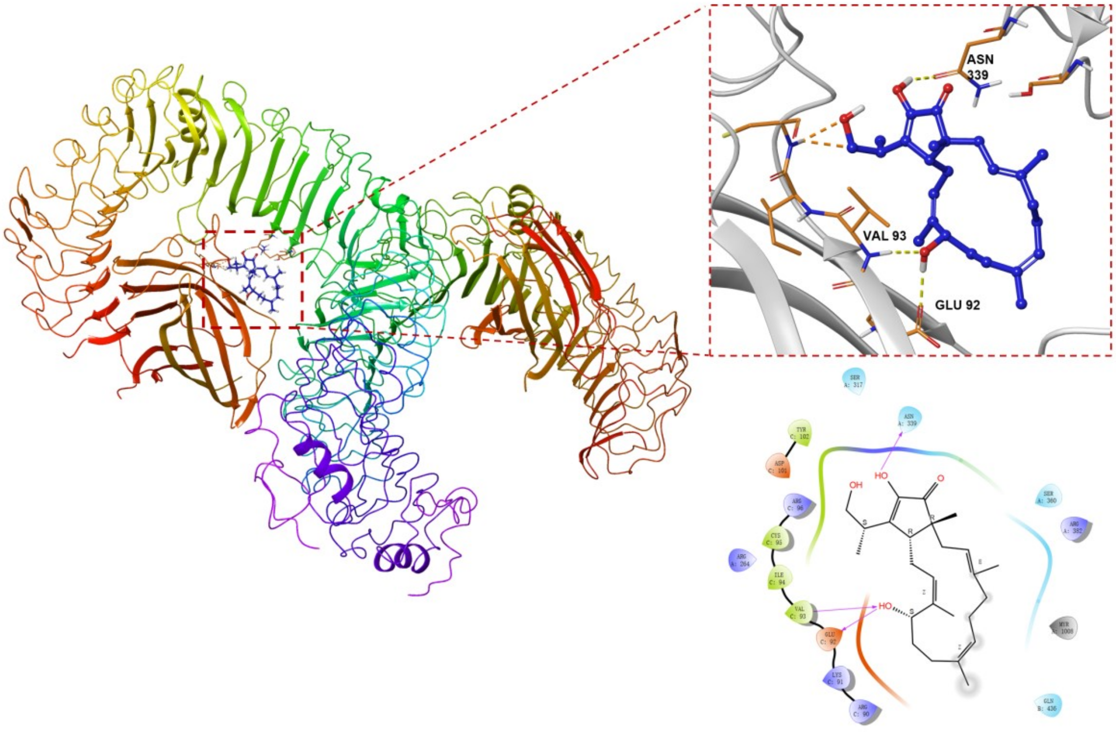

Supplementary Figure 14. Molecular docking results of **2** with TLR4-MD2. Molecular docking simulations were obtained at the lowest energy conformation. Hydrogen bonding interactions are shown by dashes.

2.16 Molecular docking results of **3** with TLR4-MD2

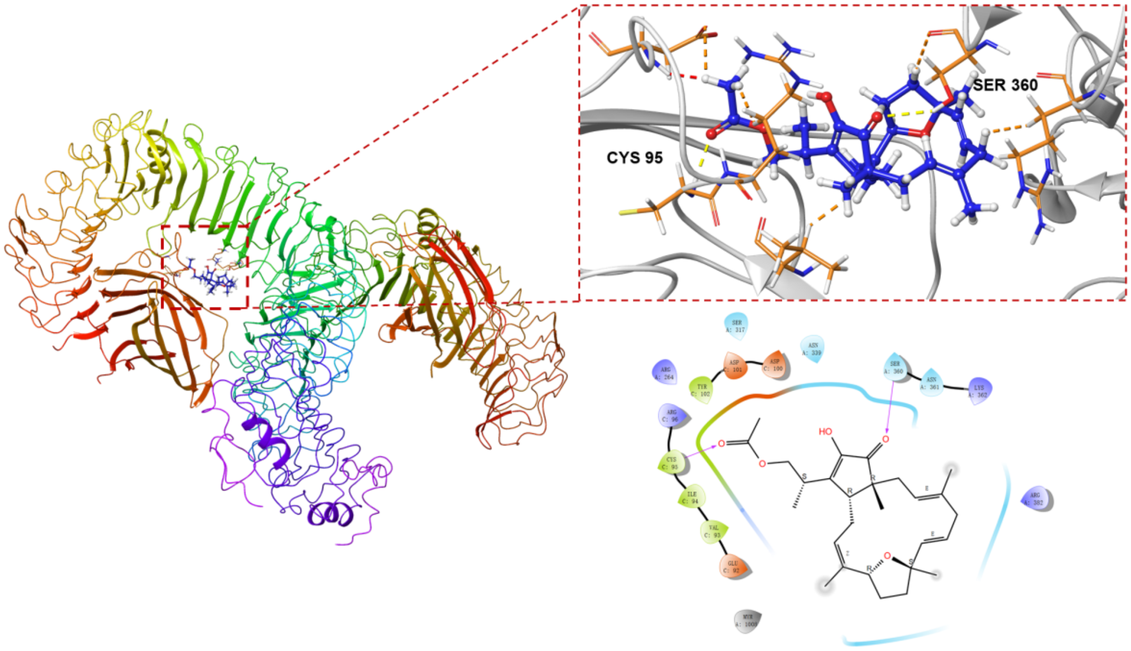

Supplementary Figure 15. Molecular docking results of **3** with TLR4-MD2. Molecular docking simulations were obtained at the lowest energy conformation. Hydrogen bonding interactions are shown by dashes.

2.17 Molecular docking results of 4 with TLR4-MD2

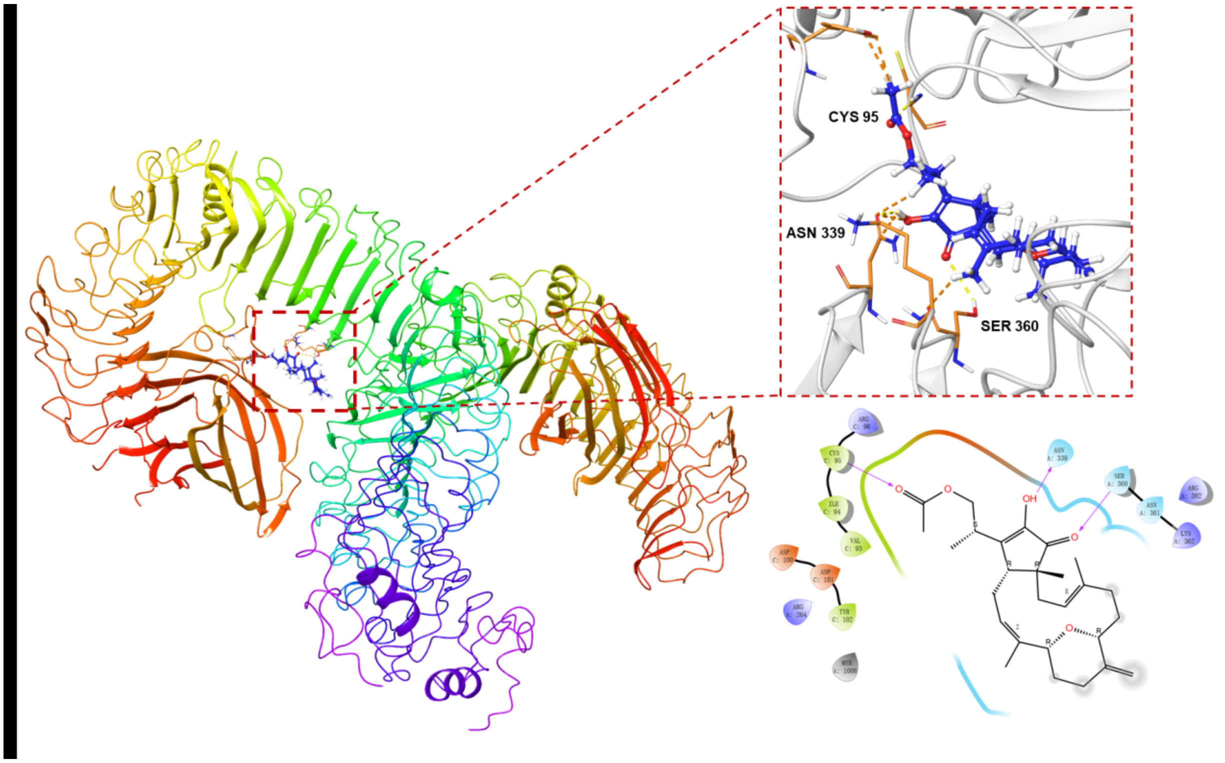

Supplementary Figure 16. Molecular docking results of **4** with TLR4-MD2. Molecular docking simulations were obtained at the lowest energy conformation. Hydrogen bonding interactions are shown by dashes.

2.18 Supplementary Tables S2. The affinity curve fitting for 1-4 carried out with Biacore T200 evaluation software by global fitting using a steady-state affinity model to obtain the affinity constant KD

Compound	KD (M)	Rmax (RU)	offset (RU)	Chi² (RU²)
**1**	2.864E-5	55.25	1.217	3.65
**2**	2.918E-5	56.25	21.12	2.03
**3**	2.357E-5	35.53	14.79	1.79
**4**	2.929E-5	97.00	1.372	9.44

2.19 Supplementary Tables S3. Reporting points for binding of 1-4 to TLR4 protein

**1**		**2**		**3**		**4**	
concentration (M)	Response (RU)	concentration (M)	Response (RU)	concentration (M)	Response (RU)	concentration (M)	Response (RU)

4.69E-07	1.64913	3.75E-07	20.6778	6.25E-07	13.7261	3.75e-7	-0.6875
9.38E-07	3.36734	3.75E-06	28.5972	1.25E-06	17.7924	3.75e-6	15.6115
1.88E-06	6.22005	1.50E-05	38.7031	2.50E-06	19.2712	7.5e-6	21.6546
3.75E-06	6.43306	7.50E-06	33.1776	5.00E-06	21.14	1.5e-5	34.4709
7.50E-06	10.7239	3.00E-05	48.7688	1.00E-05	25.4223	3e-5	46.5411
1.50E-05	18.3237	6.00E-05	60.6357	2.00E-05	30.3664	6e-5	68.9328
3.00E-05	29.7996			6.00E-05	40.5621		
