# Supplementary material for: Investigation of the Anti-Inflammatory Activity of Fusaproliferin Analogues Guided by Transcriptome Analysis
Source: Front Pharmacol. 2022 May 5;13:881182. doi: 10.3389/fphar.2022.881182 (PMC10136769; doi:10.3389/fphar.2022.881182)
Supplement: Supplementary file 2 [file DataSheet2.ZIP › Compound 1 and compound 2 reference.pdf]

Biosci. Biotechnol. Biochem., **66** (3), 685–688, 2002

Note

Phytotoxic Sesterterpene, 11-Epiterpestacin, from *Bipolaris sorokiniana* NSDR-011

Yoichiro NIHASHI,* Chi-Hwan LIM,** Chihiro TANAKA,*** Hisashi MIYAGAWA,*† and Tamio UENO*

**Division of Applied Life Sciences, ***Division of Science and Technology of Regional Environment, Graduate School of Agriculture, Kyoto University, Kyoto 606-8502, Japan.*
***Department of Agricultural Chemistry, Chungnam National University, Taejon, 305-764, Korea.*

Received September, 20, 2001; Accepted October 26, 2001

The structure of siccanol, a phytotoxic sesterterpene of fungal origin, was analyzed after chemical conversion by NMR spectroscopy. Siccanol was found to be an epimer of terpestacin that has been isolated from *Arthrinium* sp., and was thus renamed 11-epiterpestacin. Its stereochemistry was also identical with that of fusaproliferin, a structurally related mycotoxin from *Fusarium proliferatum*. Therefore, this sesterterpene may also be referred to as 24-deacetyl fusaproliferin. The phytotoxicity of 11-epiterpestacin was almost equal to that of terpestacin, but significantly higher than that of fusaproliferin.

Key words: 11-epiterpestacin; terpestacin; fusaproliferin; phytotoxin; sesterterpene

In the course of our search for new phytotoxic substances from plant pathogenic fungi, we have previously reported a bicyclic sesterterpene compound that we arbitrarily named siccanol (**1**).[1] A spectroscopic analysis determined the planar structure of siccanol to be identical with that of terpestacin (**2**) from *Arthrinium* sp., which had been isolated as an inhibitor of syncytium formation caused by the human immunodeficiency virus (HIV).[2,3] However, the stereochemistry of these compounds was thought to be different, because their optical rotation values differed: $[\alpha]_D$ for siccanol was $-23°$, while it has been reported to be $+26°$ for terpestacin. The absolute structure of terpestacin has been established on the basis of spectroscopic analyses and a total synthesis.[3,4]

As a compound similar in structure to terpestacin, fusaproliferin (**3**),[5,6] a mycotoxin from *Fusarium proliferatum*, has also been isolated. Its stereostructure has been established by NMR, molecular dynamics calculation, and single crystal X-ray diffraction analyses.[7,8] The results of these studies indicate that the differences between terpestacin and fusaproliferin are determined by whether the primary hydroxy group at C-24 is acetylated or not, and by the absolute configuration at C-11, one of four asymmetric centers, in these compounds. The absolute configuration at C-11 of terpestacin is *S*, while that at the corresponding carbon of fusaproliferin (referred to as C-10 in this compound) is *R*. It has also been briefly mentioned that 24-deacetylfusaproliferin, whose planar structure is the same as that of terpestacin, was present in the culture filtrate of *F. proliferatum*.[9] However, no physicochemical data has been published for 24-deacetylfusaproliferin as a comparison. Since we found that fusaproliferin was produced together with siccanol, as described later, we had assumed that siccanol was identical with 24-deacetylfusaproliferin. We now confirm this assumption by utilizing chemical and spectroscopic techniques.

The fungal strain used to produce siccanol was *Bipolaris sorokiniana* NSDR-011, previously referred to as *Drechslera siccans*.[1] This was a stock culture from Pesticide Research Institute in Faculty of Agriculture at Kyoto University. The strain had been isolated from a decayed ryegrass leaf with brown spot lesions, and was tentatively identified as non-sporulated *D. siccans*. However, re-examination of the DNA sequence of *Brn1*[10] in this strain (DDBJ Acc. No. AB055799) showed identity with that of the

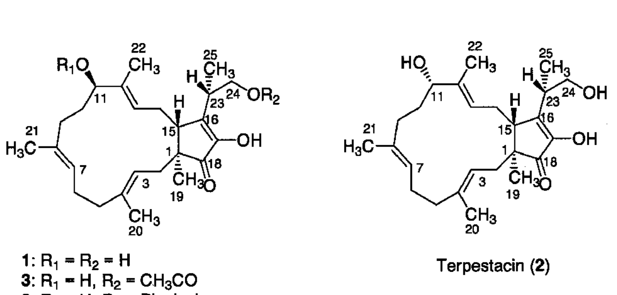

Fig. 1. Structures of the Terpestacin-related Compounds.

† To whom correspondence should be addressed. Fax: 81-75-753-6123; E-mail: miyagawa@kais.kyoto-u.ac.jp

Downloaded from https://academic.oup.com/bbb/article/66/3/685/5944835 by Chengdu University of Traditional Chinese Medicine user on 25 March 2022

Table 1. NMR Data for 11-Epiterpestacin (1) and Fusaproliferin (3)

No	1		3	
	δ_C	δ_H	δ_C	δ_H
1	49.0	—	49.0	—
2	39.9	1.75 (m)	39.2	+
		2.36 (dd, 13.7, 10.6)		+
3	121.5	5.25 (dd, 10.4, 5.4)	121.4	5.24 (m)
4	138.0	—	138.1	—
5	40.3	2.01, 2.24 (m)	40.3	+
6	23.8	2.11, 2.26 (m)	23.8	+
7	124.3	5.13 (m)	124.2	5.12 (m)
8	132.9	—	132.9	—
9	34.9	1.78, 2.18 (m)	34.9	+
10	29.8	1.70, 1.75 (m)	29.7	+
11	76.5	4.07 (dd, 9.7, 3.6)	76.5	4.06 (m)
12	136.5	—	136.4	—
13	128.9	5.38 (m)	128.9	5.38 (m)
14	28.8	1.92 (m)	28.7	+
		2.44 (d, 17.0)		
15	49.6	2.72 (dd, 11.4, 2.1)	49.9	2.79 (dd, 14.4, 7.2)
16	146.6	—	147.0	—
17	148.8	—	147.9	—
18	207.8	—	208.2	—
19	16.2	0.99 (s)	16.1	0.99 (s)
20	15.6	1.65 (s)	15.5	1.64 (s)
21	15.3	1.64 (s)	15.3	1.64 (s)
22	10.4	1.56 (s)	10.3	1.57 (s)
23	37.1	2.66 (m)	33.7	2.68 (dd, 11.1, 2.1)
24	66.1	3.80 (dd, 10.4, 5.5)	66.4	4.28 (m)
		3.85 (dd, 10.4, 7.0)		
25	14.4	1.29 (d, 7.3)	14.4	1.31 (d, 7.1)
$CH_3\underline{C}O$-			170.9	—
$\underline{CH_3}CO$-			20.8	2.03 (s)

The mark + indicates that the signal was included in the multiplet peaks found in the region δ_H 1.60–2.45 and is unassigned.

reference strains of *B. sorokiniana* (DDBJ Acc. No. AB011653). Consequently, this strain was re-identified as *B. sorokiniana* NSDR-011.

The fungus was cultured on a PSA medium in Petri dishes (90 mm i.d.) for 14 days in the dark at 25°C. The mycelial body with the agar medium was extracted with acetone. The extract was concentrated under reduced pressure, and the resulting aqueous solution was extracted with ethyl acetate. The ethyl acetate extract was concentrated and subjected to silica gel column chromatography. The column was eluted with a solvent mixture of *n*-hexane and ethyl acetate, and then with methanol. Compound **1** was detected as a phytotoxic principle, and was eluted in the methanol fraction. Compound **3** was found as the major component in the *n*-hexane-ethyl acetate (4:6–3:7) fractions, although it had little phytotoxicity. These compounds were further purified by HPLC with a Cosmosil 5C18 column, and a mobile phase of MeOH/H_2O (80/20) for **1** and (85/15) for **3**. Compounds **1** and **3** were each obtained as amorphous solid material with a respective yield of 66 mg and 27 mg from 1000 agar plates.

The obtained compounds were analyzed spectroscopically.[11,12] As has been described previously,[1] the data for **1** (Table 1) closely resembled those for **2**, except for the specific rotation value. Compound **3** was identified as fusaproliferin, based on agreement of the NMR spectral data and specific rotation value with the literature data. The coexistence of **1** with **3** in the same culture suggested that **1** was the deacetylated derivative of **3**.

The absolute configuration of C-1, C-15 and C-23 of **1** was determined to agree with that of terpestacin (**2**) by applying the same method as that used for **2**.[3] This method included the chemical conversion of **1** to **7**, as shown in Fig. 2, and spectroscopic analyses of the obtained derivatives. Correlation between the signals of H-15 and H-25 was observed in the NOESY experiments on **5**, indicating that these protons were present on the same side of the five-membered ring (Fig. 2b), while no correlation was apparent between H-15 and H-19. A *trans* orientation between the hydroxyl group at C-18 and the C-19 methyl group in **6** was evident from NOE observed between H-18 and H-19. The CD spectrum of **7** exhibited negative exciton chirality (λ_{max} 230 nm, $\Delta\varepsilon = -38.2$), indicating that the configuration of C-18 was *S* (Fig. 2c). These data are consistent with those in the literature, and therefore, the absolute configuration was concluded

Downloaded from https://academic.oup.com/bbb/article/66/3/685/5944835 by Chengdu University of Traditional Chinese Medicine user on 25 March 2022

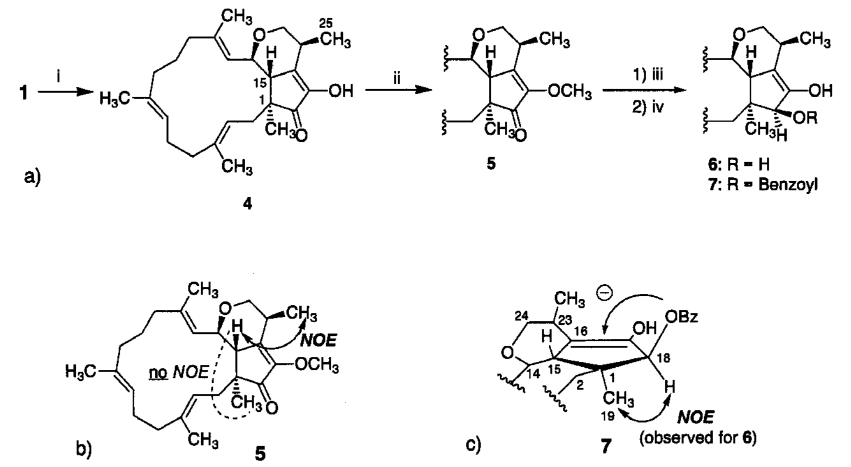

Fig. 2. Analysis of the Configurations at C-1, C-15 and C-23 of **1**.

a) Chemical conversion: i, *p*-TsOH in CH_2Cl_2; ii, TMS-diazomethane in *n*-hexane; iii, $LiAlH_4$ in THF; iv, benzoyl chloride and 4-dimethylaminopyridine in toluene. b) Stereostructure of **5**. c) Stereostructure of **7**.

Table 2. ^{1}H-NMR Data of (*R*)-(−)- and (*S*)-(+)-MTPA Esters of **1**

No.	δ_H (ppm)		$\Delta\delta_H$
	(*R*)-(−)-MTPA ester	(*S*)-(+)-MTPA ester	(*S*-*R*)
2	2.41	2.35	−0.06
	1.70	1.70	0
3	5.23	5.23	0
5	2.35	2.32	−0.03
	2.12	2.15	0.03
6	2.30	2.30	0
	2.08	2.10	0.02
7	5.15	5.16	0.01
9	1.99	2.05	0.06
	1.81	1.85	0.04
10	1.83	1.92	0.09
	1.76	1.81	0.04
11	5.45	5.42	−0.03
13	5.62	5.60	−0.02
14	2.42	2.40	−0.02
	1.93	1.88	−0.05
15	2.77	2.77	0
19	0.97	0.96	−0.01
20	1.65	1.64	−0.01
21	1.65	1.66	0.01
22	1.57	1.40	−0.17
23	2.80	2.79	−0.01
24	4.29	4.29	0
	4.24	4.24	0
25	1.32	1.32	0
Pivaloyl	1.18	1.19	
MTPA phenyl	7.43, 7.33	7.43, 7.33	

to be 1*S*, 15*R*, 23*S*.

The absolute configuration at C-11 was determined by the modified Moscher method, using α-methoxy-α-trifluoromethylphenylacetic acid (MTPA) esters.[13] After the hydroxyl group at C-24 had been protected by converting to pivaroyl ester **8**, a set of (*R*)- and (*S*)-MTPA esters (**9a** and **9b**) was prepared by reacting **8** with the respective MTPA chlorides in pyridine.

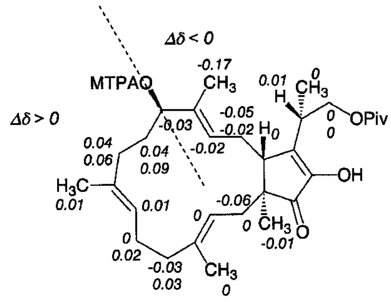

Fig. 3. Δδ Values for the MPTA Esters of **1**.

The ^{1}H-NMR spectrum of each compound was recorded in $CDCl_3$, using a Bruker ARX500 spectrometer. The differences of chemical shifts of all proton signals between **9a** and **9b** ($\Delta\delta = \delta S\text{-}\delta R$) were measured (Table 2), after a complete signal assignment by H-H and C-H COSY experiments. As shown in Fig. 3c, a systematic arrangement of Δδ values with respect to the MPTA residue was observed, which indicates that the absolute configuration at C-11 of **1** was *R*. This is opposite to the case of **2**, and is the same as that of the corresponding carbon in fusaproliferin. The data for **9a** and **9b** are in good agreement with the values for the MTPA derivatives of fusaproliferin, although only part of this data has been presented in the literature.[7] Thus, compound **1** was determined to be an epimer of terpestacin at C-11, or a deacetyl derivative of fusaproliferin at C-24.

Compound **1** almost completely inhibited the root growth of Italian ryegrass seedlings at 100 ppm. This inhibitory potency is not significantly different from that of terpestacin (**2**), which has also been isolated as a phytotoxic compound from *Bipolaris cynodontis*.[14] This shows that the absolute configuration at C-11 has no effect on the phytotoxic activity. On the other hand, **3** showed no apparent inhibition at 200 ppm,

Downloaded from https://academic.oup.com/bbb/article/66/3/685/5944835 by Chengdu University of Traditional Chinese Medicine user on 25 March 2022

indicating that the hydroxyl group at C-24 plays an important role in the expression of phytotoxicity. These results are in contrast with the cytotoxicity of these compounds. Compound **1** has been reported to have had no significant toxicity toward *Artemia salina* L. brine shrimp larvae, while **3** was highly toxic.[9]

In conclusion, the sesterterpene phytotoxin from *Bipolaris sorokiniana* was identified as 11-epiterpestacin. It is also referred to as 24-deacetylfusaproliferin.

References and Notes

1) Lim, C. H., Miyagawa, H., Ueno, T., Takenaka, H., and Sung, N. D., Siccanol: sesterterpene isolated from pathogenic fungus *Drechslera siccans*. *Agric. Chem. Biotechnol.*, **39**, 241–244 (1996).
2) Oka, M., Iimura, S., Tenmyo, O., Sawada, Y., Sugawara, M., Ohkusa, N., Yamamoto, H., Kawano, K., Hu, S.-L., Fukagawa, Y., and Oka, T., Terpestacin, a new syncytium formation inhibitor from *Arthrinium* sp. *J. Antibiot.*, **46**, 367–373 (1993).
3) Oka, M., Iimura, S., Narita, Y., Furumai, T., Konishi, M., Oki, T., Gao, Q., and Kakisawa, H., Stereochemistry and biosynthesis of terpestacin, a new syncytium formation inhibitor. *J. Org. Chem.*, **58**, 1875–1881 (1993).
4) Tatsuta, K. and Masuda, N., The first total synthesis of natural (+)-terpestacin, syncytium formation inhibitor. *J. Antibiot.*, **51**, 602–606 (1998).
5) Randazzo, G., Foliano, V., Ritieni, A., Mannina, L., Rossi, E., Scarallo, A., and Segre, A. L., Proliferin, a new sesterterpene from *Fusarium proliferatum*. *Tetrahedron*, **49**, 10883–10896 (1993).
6) Ritieni, A., Fogliano, V., Randazzo, G., Scarallo, A., Logrieco, A., Moretti, A., Mannina, L., and Bottalico, A., Isolation and characterization of fusaproliferin, a new toxic metabolite from *Fusarium proliferatum*. *Nat. Toxins*, **3**, 17–20 (1995).
7) Manetti, C., Fogliano, V., Ritieni, A., Santini, A., Randazzo, G., Logrieco, A., Mannina, L., and Segre, A. L., Determination of the structure of fusaproliferin by ^{1}H-NMR and distance geometry. *Structural Chemistry*, **6**, 183–189 (1995).
8) Santini, A., Ritieni, A., Fogliano, V., Randazzo, G., Mannina, L., Logrieco, A., and Benedetti, E., Structure and absolute stereochemistry of fusaproliferin, a toxic metabolite from *Fusarium proliferatum*. *J. Nat. Prod.*, **59**, 109–112 (1996).
9) Ritieni, A., Monti, S. M., Randazzo, G., Logrieco, A., Moretti, A., Peluso, G., Ferracane, R., and Fogliano, V., Teratogenic effects of fusaproliferin on chicken embryos. *J. Agric. Food Chem.*, **45**, 3039–3043 (1997).
10) Shimizu, K., Tanaka, C., and Tsuda, M., Cloning of *Brn1*, a reductase gene involved in melanin biosynthesis in *Cochliobolus heterostrophus*. *J. Gen. Appl. Microbiol.*, **43**, 145–150 (1997).
11) Physicochemical properties of **1**: $[\alpha]_D^{15}$ $-23°$ (c 0.6, MeOH); IR ν_{max} ($CHCl_3$) cm^{-1}: 3500, 1699, 1670; EI-MS m/z: 402(M^+), 384, 215, 187, 147, 137, 93, 81, 55; see Table 1 for the NMR data.
12) Physicochemical properties of **3**: $[\alpha]_D^{32}$ $-35.8°$ (c 1.1, $CHCl_3$); EIMS m/z: 444 (M^+), 384, 147, 135, 81, 43; see Table 1 for the NMR data.
13) Ohtani, I., Kusumi, T., Kashman, Y., and Kakisawa, H., High-field FT NMR application of Mosher's method. The absolute configuration of marine terpenoids. *J. Am. Chem. Soc.*, **113**, 4092–4096 (1991).
14) Lim, C.-H., Miyagawa, H., Ueno, T., Takenaka, H., and Tsurushima, T., Isolation and structure elucidation of terpenoid phytotoxins produced by the plant pathogenic fungus *Bipolaris cynodontis*. In *Abstracts of Papers of 37th Symposium on the Chemistry of Natural Products, Tokushima, Japan*, 325–330 (1995).

Downloaded from https://academic.oup.com/bbb/article/66/3/685/5944835 by Chengdu University of Traditional Chinese Medicine user on 25 March 2022
