# Supplementary material for: Investigation of the Anti-Inflammatory Activity of Fusaproliferin Analogues Guided by Transcriptome Analysis
Source: Front Pharmacol. 2022 May 5;13:881182. doi: 10.3389/fphar.2022.881182 (PMC10136769; doi:10.3389/fphar.2022.881182)
Supplement: Supplementary file 2 [file DataSheet2.ZIP › Compound 1_ reference_1.pdf]

Isolation and Characterization of Fusaproliferin, a New Toxic Metabolite From *Fusarium proliferatum*

Alberto Ritieni, Vincenzo Fogliano, Giacomino Randazzo, Angela Scarallo, Antonio Logrieco, Antonio Moretti, Luisa Mannina, and Antonio Bottalico
Dipartimento di Scienza degli Alimenti dell'Università degli Studi di Napoli "Federico II," Portici, Napoli (A.R., V.F., G.R., A.S.); Istituto Tossine e Micotossine da Parassiti Vegetali del C.N.R., Bari, (A.L., A.M.); Istituto di Strutturistica Chimica C.N.R., Roma (L.M.); and Istituto di Patologia Vegetale dell'Università degli Studi, Sassari (A.B.), Italy

ABSTRACT A new toxic sesterterpene, named *fusaproliferin,* was purified from corn kernel cultures (120 mg/kg dry culture) of a strain of *Fusarium proliferatum* isolated from corn ear rot in northern Italy. The strain, designated ITEM-1494, also produced fumonisin B_1 (1.500 mg/kg dry culture) and beauvericin (90 mg/kg dry culture), but not moniliformin. To monitor toxicity, the brine shrimp assay was used throughout the isolation procedure. Fusaproliferin had a molecular formula of $C_{27}H_{40}O_5$, and it is the first sesterterpene isolated from a *Fusarium* species. © 1995 Wiley-Liss, Inc.

Key Words: Sesterterpene, Mycotoxin, Fusaproliferin, *Fusarium proliferatum*

INTRODUCTION

Fusarium proliferatum (Matsushima) Nirenberg, a member of the *Liseola* Woll. section, occurs worldwide in infected plants as well as on a great variety of agricultural commodities [Nirenberg, 1976; Gerlach and Nirenberg, 1982]. Mainly together with *F. moniliforme* Sheldon, it was reported associated with corn implicated in some animal mycotoxicoses, such as leukoencephalomalacia in horses and pulmonary edema in pigs [Marasas et al., 1984; Ross et al., 1990]. It has been shown that strains of *F. proliferatum* produce several mycotoxins in culture, including moniliformin [Marasas et al., 1986], fumonisins [Ross et al., 1990], and beauvericin [Moretti et al., 1993].

A survey of *Fusarium* species causing corn ear rot in northern Italy led to the identification of some toxic strains of *F. proliferatum*. Preliminary investigation of the occurrence of toxic metabolites in culture extracts of Italian *F. proliferatum* strains showed that the toxicity to brine shrimp larvae (*Artemia salina* L.) was not related to the presence of fumonisin, moniliformin, or beauvericin. This paper describes the isolation and characterization of a new toxic metabolite from *F. proliferatum* in autoclaved corn cultures.

MATERIALS AND METHODS

Fungal Strains

Strains of *F. proliferatum* were isolated during the 1991 harvest crop from infected corn ear rot samples collected in northern Italy. The growth and colour of single-conidium colonies were studied on potato-dextrose agar (PDA), while the morphological characteristics of conidia and conidiophores were studied on carnation leaf agar (CLA) [Nelson et al., 1983]. The subcultures were incubated for 7 days at 25°C under white and black (ultraviolet) fluorescent lamps (2700 lux), for 12 h/day, and identified according to the classification system described by Nelson et al. [1983]. To preserve the cultures for toxigenic studies, mycelia and conidia from the wild strains grown on CLA were transferred aseptically into 1 ml sterile 18% (v/v) aqueous glycerol and frozen at −75°C. The strains were deposited at the collection of the Istituto Tossine e Micotossine da Parassiti Vegetali (ITEM) in Bari, Italy.

Toxin Production

F. proliferatum ITEM-1494 was grown on 100 g corn kernels var "Plata," brought overnight to about 45% moisture in 500 ml Erlenmeyer flasks, and then autoclaved for 20 min at 120°C. Inoculation was performed using 2 ml per flask of water of conidia suspension containing approximately 10^7 conidia/ml. The harvested culture material was dried in a forced draught oven at 60°C for 48 h, finely ground, and stored at 4°C until used. Control corn cultures were obtained under the same conditions, without inoculation.

Received December 10, 1993; accepted for publication April 15, 1994.

Address reprint requests to Antonio Logrieco, Istituto Tossine e Micotossine da Parassiti Vegetali, C.N.R., Via Luigi Einaudi 51, Bari, Italy.

© 1995 Wiley-Liss, Inc.

Isolation of Toxin

The fresh material (244 g) was ground in a Waring blender and extracted by stirring with 1.2 l of absolute methanol-1% aqueous NaCl (55:45 v/v) for 48 h at room temperature. The suspension was filtered and the methanol was removed under vacuum. The aqueous residue (500 ml) was then extracted with n-hexane (250 ml, three times), and subsequently the aqueous phase was extracted exhaustively with CH_2Cl_2 (250 ml, three times). The organic extracts were combined, dried over Na_2SO_4, filtered, and evaporated under reduced pressure. The organic phase assayed by *A. salina* bioassay caused 100% mortality. The organic extracts produced an oily residue (709 mg), which was dissolved in 1 ml of $CHCl_3$-propan-2-ol (95:5 v/v) and applied to a SiO_2 (Merck 70-230 mesh) column (350 g, 100 cm high, 25 mm i.d.) and eluted with the same solvent. The first fraction collected was 50 ml, and subsequent fractions of 10 ml each were collected, monitored by thin-layer chromatography (TLC), in the same eluent, and visualized by UV light and iodine vapour. The homogeneous fractions were combined, dried under vacuum, and bioassayed using *A. salina* to check their activity. Two of 10 fractions were highly toxic and were further purified. The first fraction contained beauvericin, while the last fraction, which was more hydrophobic, produced 296 mg of a crude residue on evaporation. This fraction was dissolved in $CHCl_3$, applied to an alumina (Fluka 100-125 mesh) column, and eluted with the same solvent. The active fractions were pooled (75.8 mg) and further purified by preparative TLC run with $CHCl_3$-MeOH (95:5 v/v). A fluorescent band at R_f 0.73 was detected at 254 nm, scraped from the plates, extracted by MeOH, and dried under vacuum. This procedure yielded 29.8 mg of pure product, which migrated as a single compound on TLC in two different solvent systems on SiO_2: $CHCl_3$-MeOH (95:5 v/v, $R_f = 0.73$) ethyl acetate-n-hexane (50:50 v/v, $R_f = 0.60$), and by reversed phase Rp18 plates run with CH_3CN-H_2O (75:25 v/v, $R_f = 0.50$).

Chemical Apparatus

^1H and ^{13}C NMR spectra were recorded in a $CDCl_3$ solution with a Bruker AMX600 at 600.13 MHz for ^1H and 150.92 MHz for ^{13}C. Low resolution electronic impact mass spectrometry data were obtained using a TRIO 2000 Fisons at 70 eV, 400 μA, with a source temperature of 200°C. The IR spectra were recorded on a Perkin-Elmer 399 instrument in $CHCl_3$; UV spectra were measured on a spectrophotometer (Kontron Uvikon 930) in MeOH. The optical rotation was measured on a Perkin-Elmer 141 polarimeter. The melting point (uncorrected) was measured using a Gallenkamp melting point apparatus. Elemental analysis was determined using a Carlo Erba 1106 Elemental Analysis instrument. All solvents were spectral grade for spectroscopy. Analytical and preparative TLC were performed on SiO_2 (silica gel, Merck plates F_{254}, 20 × 20 cm thickness, 0.25 and 0.5 mm) and reversed phase Rp18 (Whatman F_{254}, 20 × 20 cm thickness 0.25 mm). The spots were visualized after air-drying by exposure to UV light and/or exposure to iodine vapour.

Characterization of Fusaproliferin

After adding n-hexane to an ethyl acetate solution of the pure compound an amorphous solid mp 142–147°C was obtained, $[\alpha]^{25}_D$-35° (c = 0,255, MeOH). IR ($CHCl_3$); 1728, 1708, 1663 cm^{-1}; λ_{max} nm 261 (ϵ = 6000). MS: $[M^+]$ 444 m/z; $[M^+$-$CH_3COOH]$ 384; $[M^+$-$H_2O]$ 426. ^1H-NMR (600.13 MHz, $CDCl_3$); δ 1.70 (s, J = 6.6, 13.6 Hz, 1H), 2.38 (m, J = 10.6, 13.6 Hz, 1H), 5.24 (m, 1H), 2.30 (m, 1H), 2.01 (m, 1H), 2.30 (m, J = 8.8 Hz, 1H), 2.13 (m, J = 4.5 Hz, 1H), 5.12 (m, 1H), 2.11 (m, 1H), 1.78 (m, 1H), 1.77 (m, 4 Hz, 1H), 1.68 (m, 10 Hz, 1H), 4.05 (dd, 1H), 5.38 (m, 1H), 2.40 (m, 3, 17, 2.5 Hz, 1H), 1.92 (m, 6-7, 17, 11.1 Hz, 1H), 2.67 (dd, 11,1 Hz, 1H), 2.78 (m, 1H), 1.64 (s, 3H), 1.64 (s, 3H), 1.56 (s, 3H), 0.99 (s, 3H), 4.28 (m, 7.6, 10.6 Hz, 2 × 1H), 4.25 (m, 6.9 Hz, 1H), 1.31 (d, 2 Hz, 3H), 2.02 (s, 3H), 5.56 (s, 1 H), 1.61 (m, 1H). ^{13}C-NMR (150.92 MHz, $CDCl_3$): δ 207.86, 170.91, 147.27, 146.71, 138.20, 136.54, 132.93, 128.89, 124.31, 121.38, 76.51, 66.43, 49.56, 49.01, 40.33, 39.14, 34.93, 33.71, 29.72, 28.72, 23.83, 16.19, 15.55, 15.32, 14.52, 10.38. Analysis calculated for $C_{27}H_{40}O_5$: C, 72.94; H, 9.07. Found C, 72.53; H, 8.77.

Fumonisin B_1, Moniliformin, and Beauvericin Analyses

Analysis of fumonisin B_1 was performed according to the procedures of Shephard et al. [1990]. Briefly, the residue was dissolved in 1 ml methanol and compared by TLC and HPTLC with a standard kindly provided by Dr. R. Vesonder (USDA, Peoria, IL), using two different solvent systems: chloroform-methanol-acetic acid (55:36:8:1 v/v) and chloroform-methanol (60:40 v/v). Identification and quantitative analyses of moniliformin and beauvericin were performed according to the method previously described using TLC [Bottalico et al., 1989] and high-performance thin-layer chromatography (HPTLC) [Logrieco et al., 1993], respectively. The moniliformin and beauvericin standards were purchased from Sigma Chemical Co. (St. Louis, MO).

Brine Shrimp Bioassay

The toxicity of the culture extracts and fractions of column eluates were tested on brine shrimp larvae (*Artemia saline* L.) according to the procedure of Bottalico et al. [1989]. Briefly, the bioassay was performed in cell culture plates (Corning, New York) with 24 wells containing about 30–40 larvae in 500 μl sea water per well, using toxic test solutions containing 1% (v/v) methanol (4 replicates per test). The number of dead shrimp was recorded after incubation at 27°C for 24 h. The total number of shrimp in each well was measured after killing the remaining shrimp by freezing at −20°C for 12 h.

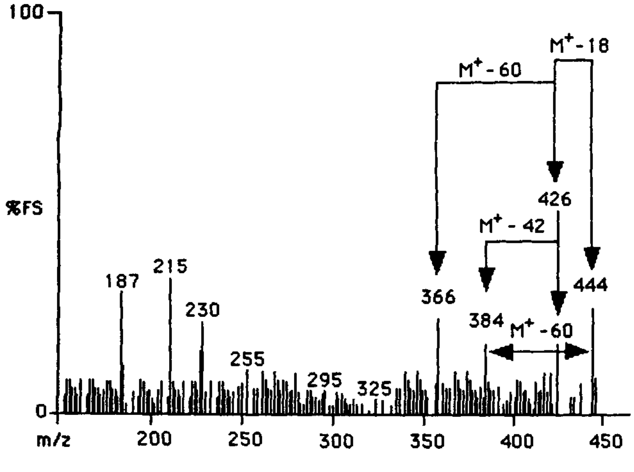

Fig. 1. Ms spectrum (EI) of fusaproliferin.

RESULTS AND DISCUSSION

Single conidia cultures of *F. proliferatum* ITEM-1494 showed slightly floccose aereal mycelium and the presence of a greyish-white pigment after 2–3 days, which turned into a dark vinaceous after 7–10 days. This strain grew quickly (colony of PDA 6.6–7.5 cm diameter in 7 days, 25°C), forming abundant microconidia, both in chains and in false heads, first from monophialides and later also from polyphialides. The microconidia were club shaped, mostly without septate, with a flattened base. No pear-shaped microconidia were observed. The macroconidia were produced from monophialides on branched conidiophoras in sporodochia, and were mostly 3-septate, measuring 34 ± 4 × 2.8 ± 0.6 μm. Chlamydospores were absent. The identity of ITEM-1494 as *F. proliferatum* was confirmed by Prof. P.E. Nelson, who deposited the strain in the Collection of the Fusarium Research Center (University Park, PA; accession number M-7308).

Crude methanolic extract from autoclaved corn cultures were highly toxic in the brine shrimp assay and caused 100% mortality within 24 h. Both fumonisin B_1 and moniliformin were not considered responsible for this toxicity because they should remain in the aqueous phase during extraction. Fumonisin B_1 (1.500 mg/Kg dry culture), but not moniliformin was indeed found in the aqueous phase. Of the 10 fractions obtained from chromatography on SiO_2, 2 showed a high toxicity towards *A. salina*. One of these fractions contained the well-known compound beauvericin (90 mg/kg dry culture). Consequently, investigations were directed to the other toxic fraction. The purification procedure yielded 29.8 mg of the pure substance (120 mg/kg of culture).

This unknown toxic substance produced a molecular peak m/z 444 in MS (Fig. 1). The ^{13}C NMR spectrum contained 27 signals and, according to their multiplicity due to direct couplings, there were 38 protons attached to carbons in the molecule. According to the ^{13}C NMR spectrum, there should be one OCH at 76.51 ppm, one OCH_2 at 66.43 ppm, one ester carbonyl at 171.91 ppm, and one ketone carbonyl at 207.86 ppm. The 3-proton singlet at 2.02 ppm in the ^1H NMR spectrum indicates the presence of an acetate group. Its presence in the molecule was confirmed by the M^+-60 peak in the MS spectrum and by an IR absorption at 1728 cm^{-1}. The chemical shift of the OCH_2 carbon at 66.43 ppm suggested that this group is acetylated. The IR spectrum also corroborates the presence of a ketone (ν_{max} 1708 cm^{-1}) [Nakanishi, 1962]. To account for the observed molecular ion, the molecule had to contain 5 oxygen atoms, which means 2 OH groups. The presence of 2 OH groups was proved by the formation on an acetate derivative of the compound that exhibited M^+ at 528 (an increase of 84 amu, i.e., 2 CH_3CO) and 3 acetyl singlets in the ^1H NMR spectrum.

The deduced molecular formula, $C_{27}H_{40}O_5$, contained 8 unsaturations. Two carbonyls and 4 double bonds (one of them tetrasubstituted) inferred from ^{13}C NMR data account for 6 unsaturations. Therefore, the molecule had to contain 2 rings. Because there was an extensive overlap of proton signals in the 1.65–2.40 ppm region, the structure of the compound was determined using 2D NMR strategies (DEPT, COSY, NOESY, TOCSY, HMBC). Details of this study are presented elsewhere [Randazzo et al., 1993].

In the proposed structure (Fig. 2) 2 anomalous rings were present: The larger one was a macrocycle, which is uncommon in natural products, whereas the smaller one contained an enolic OH near the keto group. Only one proton was present in this ring, so its structural determination using NMR was a remarkable success. In fact, the keto-enol tautomerism was hampered by the stiffness of the 5-membered ring, and this explains the width of the OH proton signal at 5.56 ppm (1H). UV data also confirmed this system: (RCO-C(OH) = CR) [Silverstein et al., 1981].

Interpretation of all the data led to a molecular structure with a isoprenoid nature that belonged to the sesterterpene family, as it was possible to identify 5 isoprenic units linked according to the "biogenetic isoprene rule" [Cordell, 1974]. This class of compounds is known to be active in a variety of bioassays [Crews et al., 1991], the most notable of which refers to anti-inflammatory properties [Reynolds et al., 1988]. Fusaproliferin has a new skeleton terpene ring, which has not been previously found in any known sesterterpene. Moreover, this toxic metabolite is the first sesterterpene reported in *Fusarium* species.

Although *F. proliferatum* is a recently described species, it is widely distributed in temperate countries, including Italy. It is usually involved in a wide range of plant diseases [Gerlach and Nirenberg, 1982], including corn stalk and ear rot [Logrieco and Bottalico, 1988; Leslie et al., 1990; Nirenberg, 1976]. The capability of *F. proliferatum* ITEM-1494 to produce fusaproliferin, in addition to other well-established toxins, such as fumonisin B_1 [Ross et al., 1990] and beauvericin [Moretti et al., 1993], suggests that extending the investigation of fusaproliferin production to other close

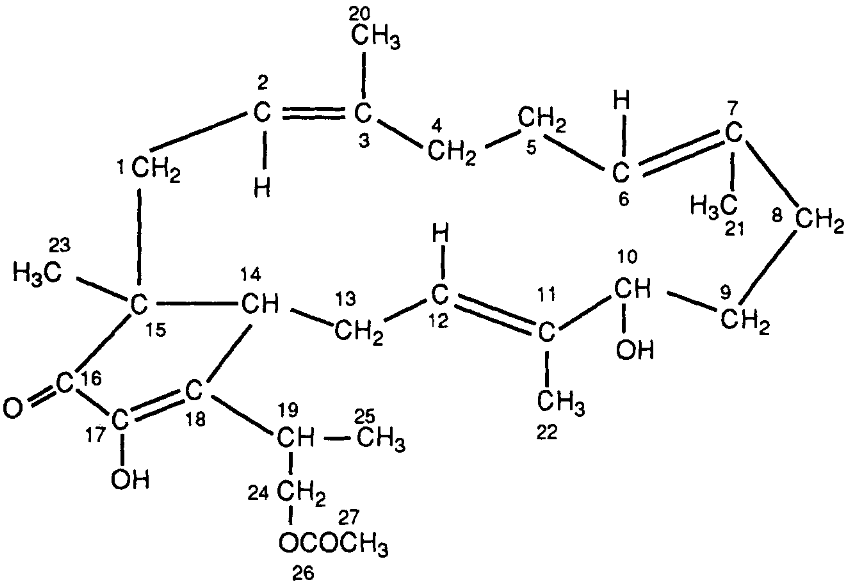

Fig. 2. Chemical structure of fusaproliferin.

phylogenetic *Liseola* species, such as *F. moniliforme* and *F. subglutinans* would be worthwhile. Moreover, *A. salina* proved to be a reliable test organism for monitoring toxic activity during purification [Harwig and Scott, 1971], but further studies on the biological properties of fusaproliferin are obviously needed to assess its role in plant and animal diseases.

We are grateful to Dr. Ferracane Rosalia for her technical assistance; to Dr. Vesonder of the Northern Regional Research Center, the U.S. Department of Agriculture, Peoria, Illinois, for fumonisin B_1 standard; and Prof. Nelson of Pennsylvania State University for taxonomic assistance.

REFERENCES

Bottalico A, Logrieco A, Visconti A (1989): *Fusarium* species and their mycotoxins in infected cereals in field and in stored grains. In Chelkowski J (ed): "*Fusarium*—Mycotoxins, Taxonomy and Pathogenicity." Amsterdam: Elsevier Press, pp 85–119.

Cordell GA (1974): The occurrence, structure elucidation and biosynthesis of the sesterterpenes. Phytochemistry 13:2343–2364.

Crews P, Jimenez C, O Neil-Johnson M (1991): Using spectroscopic and database strategies to unravel structures of polycyclic bioactive marine sponge sesterterpenes. Tetrahedron 47:3585–3600.

Gerlach W, Nirenberg H (1982): The genus *Fusarium*—a Pictorial Atlasmitteilungen aus der Biologischen Bundesanstalt fur Land und Forstwirtschaft. Berlin: Dahlem, p 406.

Harwig J, Scott PM (1971): Brine shrimp (*Artemia salina* L.) larvae as a screening system for fungal toxins. Appl Microbiol 21:1011–1016.

Leslie JF, Pearson CAS, Nelson PE, Toussoun TA (1990): *Fusarium* spp. from corn, sorghum and soybean fields in the central and eastern United States. Phytopathology 80:343–350.

Logrieco A, Bottalico A (1988): *Fusarium* species of the *Liseola* section associated with stalk and ear rot of maize in southern Italy, and their ability to produce moniliformin. Trans Br Mycol Soc 90:215–219.

Logrieco A, Moretti A, Ritieni A, Chelkowski J, Bottalico A, Randazzo G (1993): Natural occurrence of beauvericin in pre-harvest *Fusarium subglutinans* infected corn ears in Poland. J Agric Food Chem 41:2149–2152.

Marasas WFO, Nelson PE, Toussoun TA (1984): "Toxigenic Fusarium species—Identity and Mycotoxicology." University Park: Pennsylvania State University Press, p 328.

Marasas WFO, Thiel PG, Rabie CJ, Nelson PE, Toussoun TA, Van Wyk PS (1986): Moniliformin production in *Fusarium* section *Liseola*. Mycologia 78:242–247.

Moretti A, Logrieco A, Bottalico A, Ritieni A, Randazzo G (1993): Production of beauverican by *Fusarium proliferatum* from corn, in Italy. Abs. of 6th International Congress of Plant Pathology, Montreal, p 442.

Nakanishi K (1962): "Infrared Absorption Spectroscopy." San Francisco: Holden-Day, p 233.

Nelson PE, Toussoun TA, Marasas WFO (1983): "*Fusarium* Species. An Illustrated Manual for Identification." University Park: Pennsylvania State University Press, p 193.

Nirenberg H (1976): "Under suchungen uber die Morphologische und Biologische Differenzierung in der *Fusarium*-sektion Liseola" Mitt. Biol. Bundesanst Land-Forstwirtsch. Berlin: Dahlem, 169:1–117.

Randazzo G, Fogliano V, Ritieni A, Mannina L, Rossi E, Scarallo A, Segre AL (1993): Proliferin, a new sesterterpene from *Fusarium proliferatum*. Tetrahedron 49:10883.

Reynolds LJ, Morgan B, Hite GA, Mihelich ED, Dennis EA (1988): Phospholipase A_2 inhibition and modification by manoalogue. J Am Chem Soc 110:5172–5175.

Ross PF, Nelson PE, Richard JL, Osweiler JF, Rice LG, Plattner RD, Wilson TM (1990): Production of fumonisins by *Fusarium moniliforme* and *Fusarium proliferatum* isolates associated with equine leukoencephalomalacia and a pulmonary edema syndrome in swine. Appl Environ Microbiol 56:3225–3226.

Shephard GS, Sydenham EW, Thiel PG, Gelderblom CA (1990): Quantitative determination of fumonisins B_1 and B_2 by high-perfomance liquid chromatography with fluorescence detection. J Liquid Chromatogr 13: 2077–2087.

Silverstein RM, Bassler CG, Morril CT (1981): "Spectrometric Identification of Organic Compounds. 4th ed." New York: John Wiley & Sons, p 442.
