# Supplementary material for: Investigation of the Anti-Inflammatory Activity of Fusaproliferin Analogues Guided by Transcriptome Analysis
Source: Front Pharmacol. 2022 May 5;13:881182. doi: 10.3389/fphar.2022.881182 (PMC10136769; doi:10.3389/fphar.2022.881182)
Supplement: Supplementary file 2 [file DataSheet2.ZIP › Compound 1_ reference_2.pdf]

Tetrahedron Vol. 49, No. 47, pp. 10883-10896, 1993
Printed in Great Britain

0040-4020/93 $6.00+.00
© 1993 Pergamon Press L

Proliferin, a New Sesterterpene from Fusarium proliferatum

Giacomino Randazzo,[a*], Vincenzo Fogliano[a], Alberto Ritieni[a], Luisa Mannina[b] Enrico Rossi[b], Angela Scarallo[c] and Anna Laura Segre[d].

[a]*Istituto di Industrie Agrarie dell'Università degli Studi di Napoli "Federico II", Parco Gussone, 80055 Portici, Napoli, Italy;* [b]*C.N.R. Area della Ricerca di Roma,* [c]*CO.E.PO.;* [d]*Istituto di Strutturistica Chimica C.N.R., Roma.*

(*Received in UK* 30 *July* 1993; *accepted* 17 *September* 1993)

ABSTRACT: A new bicyclic sesterterpene, named proliferin, has been characterized from Fusarium proliferatum. The pure compound exhibit toxic activity. The structure elucidation of the molecula was accomplished using data from 2D-NMR strategies. Proliferin was shown to have a novel ring skeleton and molecular formula $C_{27}H_{40}O_5$

INTRODUCTION

Our laboratory has a continuing interest in the chemistry of mycotoxin produced by fungi. In this paper we report the structure determination of a new sesterterpene isolated from Fusarium proliferatum (Matsushima) Nirenberg, a member of the Liseola Woll. section, that occurs worldwide as a pathogen on host plants[1,2]. The new compound shows toxic activity on Artemia Salina L. bioassay. This fungal strain is known to produce other toxin[3,4,5] whose chemical nature is quite different from terpenes.

The usual spectral methodologies (UV, IR, MS, and 1D ^1H and ^{13}C NMR) do not allow one to obtain an univocal structure, so the molecular structure is achieved by the new generation of 2D-NMR spectroscopy; in particular the inv4lplrnd sequence has proved to be a very helpful tool to gather the microstructure.

RESULTS AND DISCUSSION

F. proliferatum is a fungus that makes maize ear rot. It was recently isolated and identified in northern Italy. Methanolic extract of *F. proliferatum*, grown on autoclavated corn kernels, was highly toxic on brine shrimp bioassay, causing 100% of mortality of larvae within 48 h. In the water phase of colture, fumonisin was found[4], while two different toxic compounds were isolated in methanolic extract. The former was identified as beauvericin[5], while the latter, rather hydrophobic, was purified as described in experimental section obtaining the pure compound.

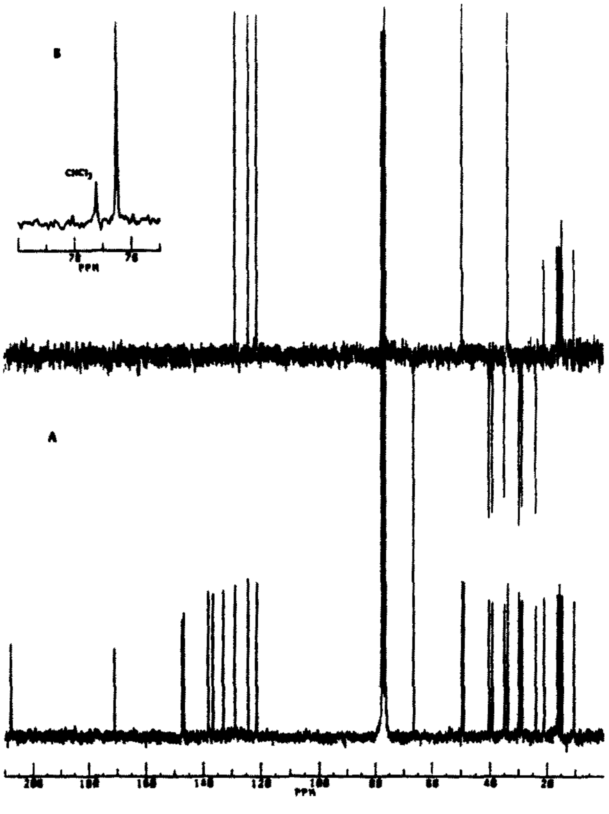

Figure 1. Bottom A: 50,3 MHz, {H} Waltz broad band decoupled, ^{13}C NMR spectrum of proliferin. Top B: the DEPT 135° is shown; in the small insert, the CH resonance at 76,51. The $CDCl_3$ is zeroed by the DEPT sequence.

A preliminary characterization was achieved using traditional spectroscopic methods (MS, UV, IR) togheter with preliminary NMR spectra run on a Bruker AC200 spectrometer. ^{13}C {1H} spectrum Waltz broad band decoupled (Fig. 1A)[6] and 135° DEPT edited (Fig.1B)[7] showed the presence of 6 CH_3 groups, 7 CH_2 groups, 6 CH groups and 8 non-bearing protons C atoms.

The ^1H spectrum, see Fig. 2, showed the presence of one OH group at 5.56 ppm and a sharp signal at 2.02 ppm (3H) that well account for the presence of an acetyl group. This group was also confirmed by IR (ν_{max} 1728 cm^{-1}), ^{13}C spectrum (peak at 171.91 ppm) and MS that gave M^+-60 signal. Moreover ^{13}C gave a peak at 207.86 ppm due to a keto group, ν_{max} 1708 cm^{-1}. Data obtained up to now clearly have proved the presence of 27 C, 39 H and 4 O. The reasonable ipothesis of another OH group arose to account for the MS data that gave M^+ 444 m/z. The presence of two OH groups was confirmed by MS of acetylated derivative (m/z 528; M^+ + $2CH_3CO$). Thus, the molecular formula was $C_{27}H_{40}O_5$; it suggested the occurence of 8 unsaturation: in fact two C=O groups were present, and ^1H and ^{13}C spectra suggested the presence of four double bounds (one, detectable only in the ^{13}C spectra, must be fully substituted); thus considering the observed molecular formula the molecula must have two rings. The assignment of proton signals between 1.65 ppm and 2.40 ppm was quite hard as the presence in this region of six CH_2 with non-equivalent proton maked the ^1H spectrum too overlapped. That is why, a Bruker AMX600 spectrometer was used for the following experiments.

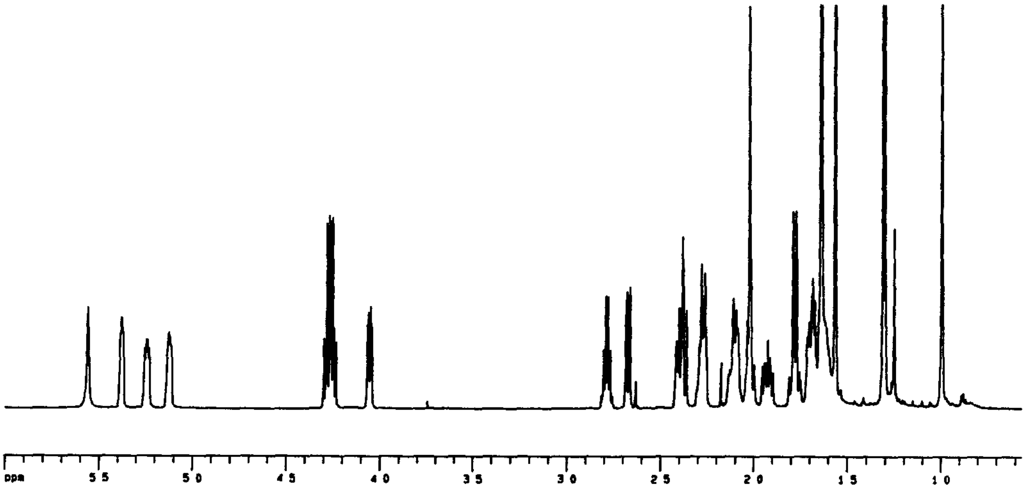

Figure 2. 600,13 MHz, ^1H NMR spectrum of proliferin.

To get the complete microstructure it was necessary to perform 2D-NMR experiments. ^1H-^1H COSY[8], not shown, allows one to identify five different spin systems, which were confirmed by TOCSY[9] spectrum, Fig. 3. Five independent spin systems, Fig. 4, were outlined as follows: (in parenthesis the final assignment is shown)

1) ABC X3 : 1,70 (1'); 2,38 (1"); 5,24 (2'); 1,64 (20).
2) ABCD XY3: 2,30(4'); 2,01(4"); 2,30(5'); 2,13(5"); 5,12(6'); 1,64(21).
3) ABCD X: 2,11(8'); 1,78 (8"); 1,77 (9'); 1,68 (9"); 4,05(10').
4) ABC XY3: 2,67(14'); 2,40(13');1,92(13"); 5,38(12'); 1,56 (22)
5) ABC X3: 2,78 (19'); 4,28 (24'); 4,25 (24"); 1,31 (25)

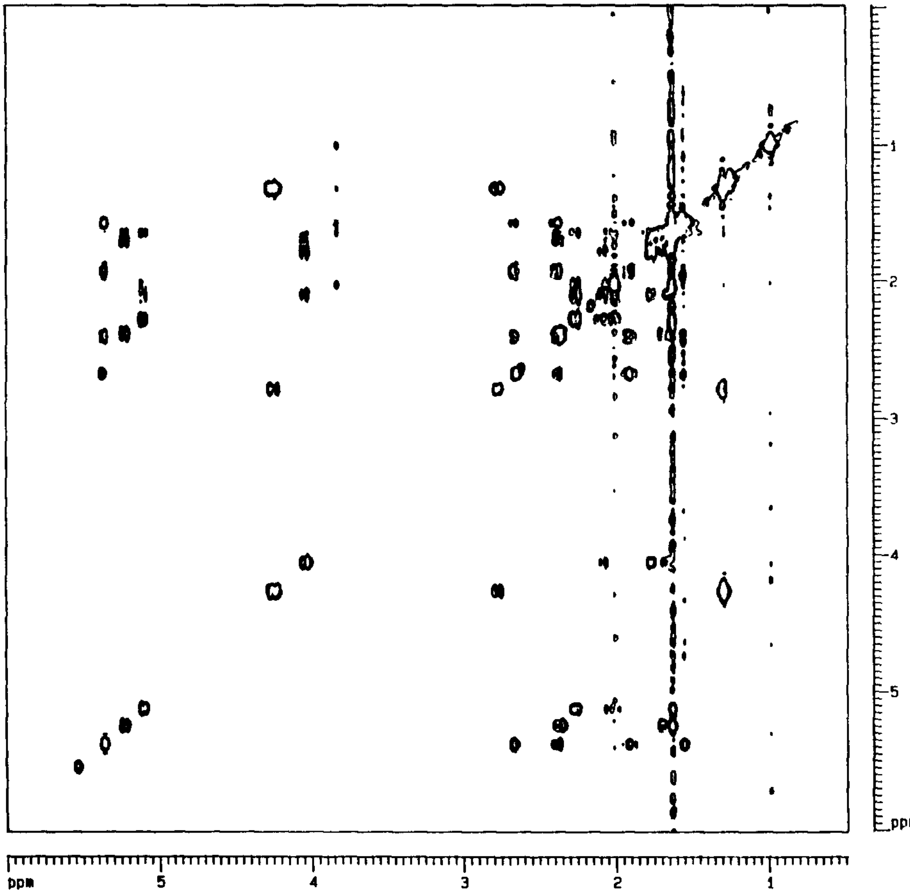

Figure 3. 600,13 MHz, ^1H-^1H TOCSY map of proliferin.

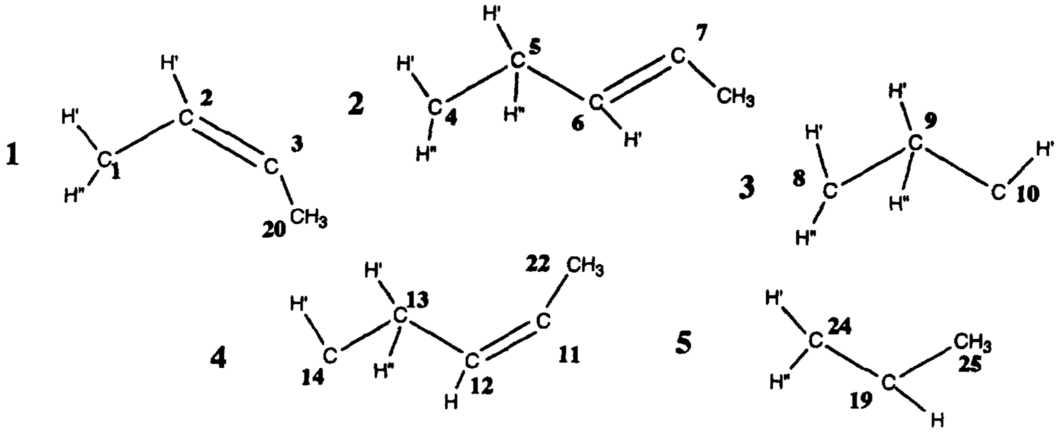

Figure 4. Sketch of spin system obtained from ^{1}H-^{1}H COSY and ^{1}H-^{1}H TOCSY.

The ^{13}C resonances, corresponding to the five spin systems, can be found in the not shown ^{1}H-^{13}C correlated map, obtained with hxcobicp sequence, i.e. a 2D heteronuclear shift correlation with ^{1}H-^{1}H decoupling in F1, using a bird pulse[10,11,12]; thus all protons bearing C atoms were assigned and were reported in Table I.

It is worth noting that this approach strictly matches literature suggestions for unraveling unknown structures[13]; however for our molecula, due to the high number of unsaturation and quaternary C atoms, the necessity of a full assignment of C spectrum arose. In fact, we did not know anything about the position of three groups: i.e. the full substituted double bond, the keto group (C-16) and the quaternary C-15. In order to connect the different spin systems and to assign quaternary C atoms resonances, an inv4lplrnd experiment was perfomed[14], i.e. a 2D ^{1}H-^{13}C correlation in reverse detection via heteronuclear zero and doublequantum coherence, see Fig. 5. The user sequence was able to connect quaternary C atoms to any proton second o third neighbour; note that the J coupling constant used in the experiment was only 6 Hz[15]. It is also worth noting that, notwithstanding the absence of decoupling and a low-pass J filter (J one bond assumed 150 Hz), CH groups with two o three bond connections can be well observed. The chemical shift of some 2D cross peaks does not correspond to any peak of the proton decoupled carbon spectrum, but it corresponds to the "in phase" component of the undecoupled spectrum, which, to clarify the assignment, is reported in F1 of Fig. 5 instead of a projection. For each CH_2 groups, the assignment is easier; in fact the relative cross peak matches the central peak of the CH_2 triplet, which is equal to the correspondent fully

Table I ^{1}H-and ^{13}C NMR data

C nr.	type	13C (ppm)	1H (ppm)	J (Hz)
1	CH2	39,14	H'=1,70	1'-2'=6,6
			H"=2,38	1"-2'=10,6
				1'-1"=13,6
2	CH	121,38	5,24	
3	C	138,2		
4	CH2	40,33	H'=2,30	
			H"=2,01	
5	CH2	23,83	H'=2,30	5'-6'=8,8
			H"=2,13	5"-6'=4,5
6	CH	124,31	5,12	
7	C	132,93		
8	CH2	34,93	H'=2,11	
			H"=1,78	
9	CH2	29,72	H'=1,77	9'-10'=4
			H"=1,68	9"-10'=10
10	CH	76,51	4,05	
11	C	136,54		
12	CH	128,89	5,38	
13	CH2	28,72	H'=2,40	13'-12'=3
			H"=1,92	13"-12'~6-7
				13'-13"=17
14	CH	49,56	2,67	14'-13'=2,5
				14'-13"=11,1
15	C	49,01		
16	C	207,86		
17	C	147,27		
18	C	146,71		
19	CH	33,71	2,78	
20	CH3	15,55	1,64	
21	CH3	15,32	1,64	
22	CH3	10,38	1,56	
23	CH3	16,19	0,99	
24	CH2	66,43	H'=4,28	24'-19'=7,6
			H"=4,25	24"-19'=6,9
				24'-24"=10,6
25	CH3	14,52	1,31	
26	C	170,91		
27	CH3	20,91	2,02	
	OH (17)		5,56	
	OH (10)		1,61	

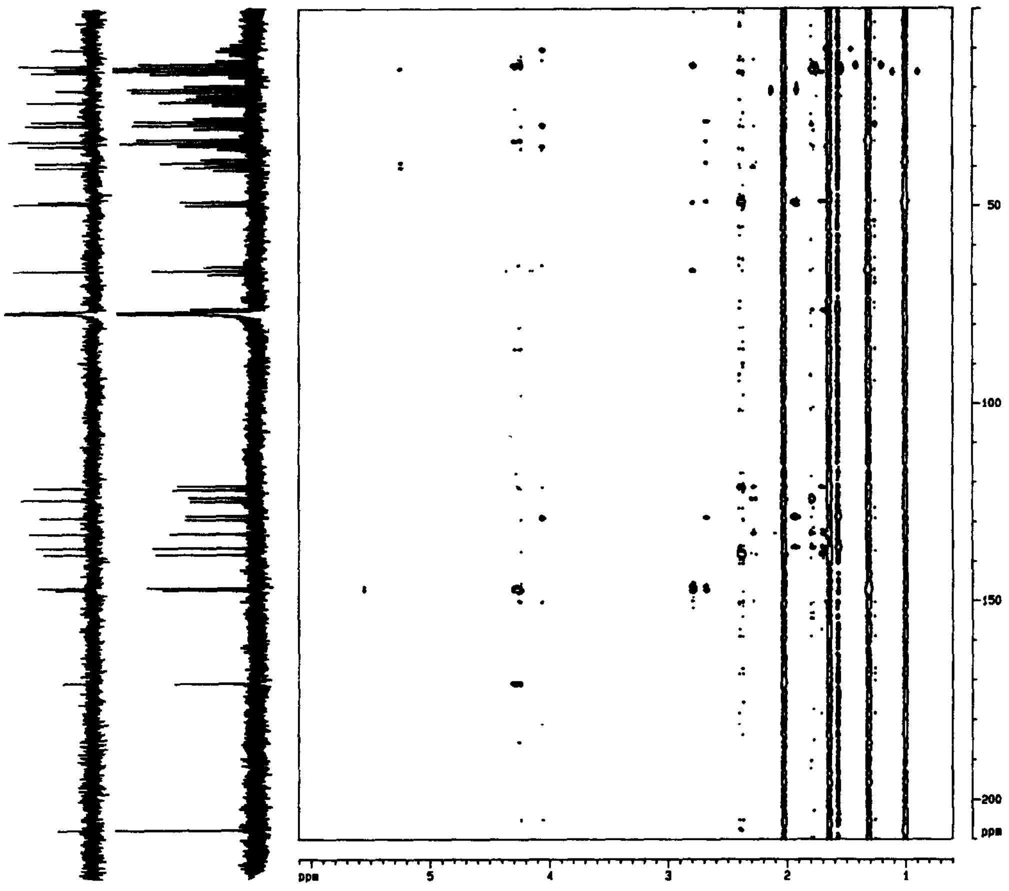

Figure 5. 2D ^1H-^{13}C correlation via heteronuclear zero and double quantum coherence optimized on long range couplings reverse detection, no decoupling during acquisition. Instead than Fl projection a ^{13}C undecoupled is reported, and in top of this a fully decoupled spectrum is also reported. The proton frequency is 600.13 MHz.

decoupled spectrum, which is reported in top of the fully coupled carbon spectrum, both obtained at 150.92 MHz. In this way, it was possible to connect all previously mentioned spin systems (Fig. 6). Anyway the main goal of this experiment was to assign the position of non-bearing protons C atoms. As shown in Fig. 6 the H-14' plays an essential role, being the only proton on the five membered ring. This proton gave a very helpful contact with C-15, C-17 and C-18.

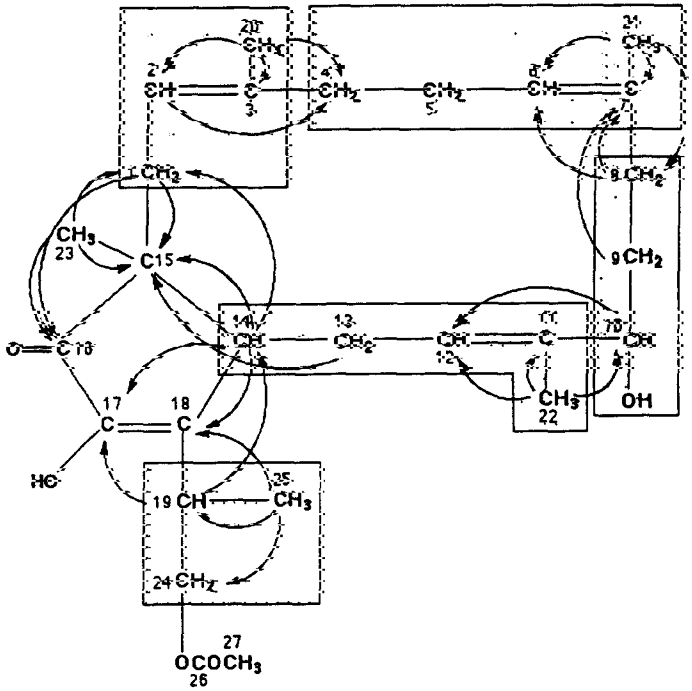

Figure 6. Sketch of structure of proliferin shown the main connection between the five spin systems obtained from the 2D map of Fig. 5.

It is important to observe that each CH_3 group gave contact with the two second neighbours allowing to link the spin systems and to position the C=O group near the quaternary carbon bearing the CH_3-23. The structure of the five membered ring was also in full agreement with UV (λ_{max} nm 261 ε= 6000)[16] and IR data (=C(OH)-C=O) 1663 cm^{-1} [17].

From all this data a chemical structure can be proposed, see Fig. 8.

A NOESY experiment[18], Fig. 7, fully confirms previous findings. Three double bonds are present in a fifteen membered ring. Noesy 2D map clearly shows that in the double bonds C(2)=C(3) and C(11)=C(12) the CH_3 and the proton are in a E

configuration (cross peak 5.24-1.64 ppm and 5.38-1.56 ppm respectively), while in the double bond C(6)=C(7) (cross peak 5.12-1.64 ppm) the CH_3 and the proton are in a Z configuration.

Full interpration of NOESY data is under progress, in order to gain the relative configuration of chiral C atoms, distances and angles. The results of all 2-D experiments are summarized in Table II.

We proposed for this compound the trivial name of proliferin. As it was possible to identify five isoprenic units this molecula is a sesterterpene. However proliferin is an unusual sesterterpene, since its skeleton ring is definitively new. Further studies are in progress to clarify the biosynthetic pathway and the absolute configuration of the four chiral centers.

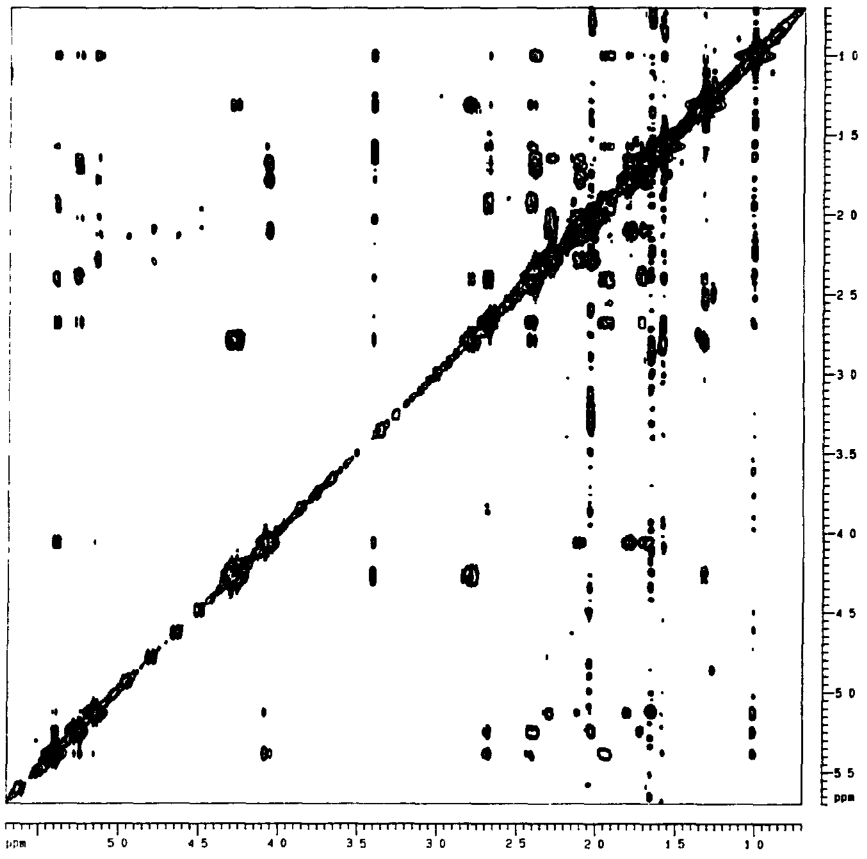

Figure 7. 600.13 MHz NOESY map, contact time 300 ms.

Table II ^{1}H and ^{13}C 2D correlation

C nr.	H	COSY	TOCSY	INV4LPLRND	NOESY
1	H'	1"; 2'		2'; 14'	14'
	H"	1'; 2'			23
2	H'	1'; 1"; (20)	20	1"; 1'; 4'; 20; 13'	14'; 12'; 23; 4"; 6'
3				20; 1'; 1"; 4'; 4"	
4	H'	4";5';5"	20;6'	2'; 5'; 20	20
	H"	4'; 5'; 5"	20; 6'		2'; 6'
5	H'	5"; 4'; 4"; 6'	21		20
	H"	5'; 4'; 4"; 6'; 21			
6	H'	5'; 5" (21)	4'; 4"; 21	5'; 5"; 4'; 4"; 8"; 9'; 21	23; 10'; 2'; 12'; 4"; 8'; 9'
7				5'; 5"; 8"; 8'; 9"; 9'	
8	H'	8"; 9'; 9"; 21	10'	10'; 9'; 9"	6'; 10'
	H"	8'; 9'; 9"	10'		23; 22
9	H'	9"; 8"; 8'; 10'		10'; 8'	23; 6'
	H"	10'; 9'; 8'; 8"			5'
10	H'	9'; 9"	8'; 8"	9'; 9"; 22	12'; 22; 6'; 8'
11				13';13";9'; 9";8";22	
12	H'	13'; 13"; 22	14'	10'; 14'; 13'; 22	10';14';23;2';22; 6'
13	H'	13"; 12'; 14'; 22		14'	19'; 25; 23; 22
	H"	13'; 12'; 14'	22		23; 22
14	H'	13'; 13"	12'; 22	1"; 13'; 13"; 23; 19'	12';2'; 22; 21;19';1';23;25
15				23;19';14';13';13";1";1'	
16				23; 1"	
17				5,56 (OH) 24'; 14' 24"; 19'; 25	
18				5,56 (OH) 24';14' 24"; 19'; 25	
19	H'	24'; 24"; 25		24'; 24";14'; 25	13'; 14'; 20
20	3H	2'	4'; 4"	2'	4'; 5'
21	3H	8'; 5"	5'; 4'; 4"	8"; 9'	14'
22	3H	13'; 12'	14' ; 13"	10'	13";13';14'; 8";10'; 12'
23	3H			1"; 1'	1"; 12';13"; 14'; 8" 2'; 6'
24	H'	19'; 24"	25	19'; 25	25
	H"	19'; 24'	25		25
25	3H	19'	24'; 24"	24'; 24"; 19'	13'; 24'; 24"
26				24'; 24"; 27	

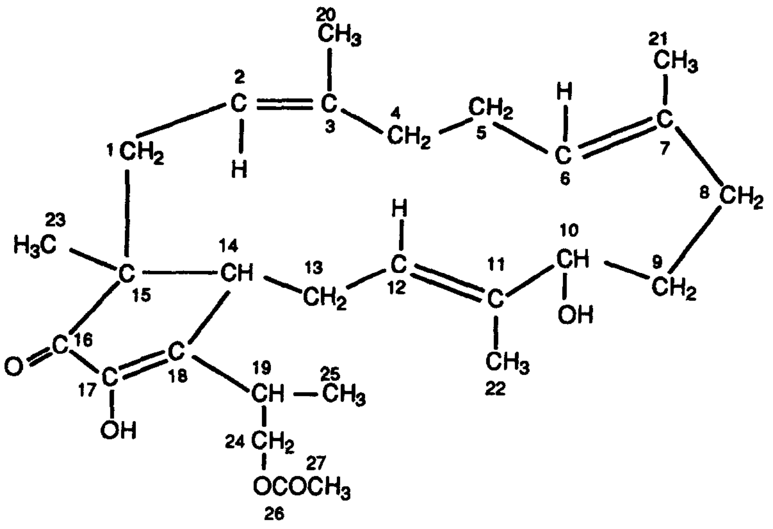

Figure 8. Sketch of structure of proliferin. The configuration of double bond is assigned with a noesy experiment, see Fig 7.

EXPERIMENTAL SECTION

Proton and Carbon NMR spectra were run in $CDCl_3$ (5 mg/ml) on a Bruker AM200 and on a Bruker AMX600 spectrometers operating at 200.13 and 50.33 and 600.13 and 150.92 MHz respectively. Literature pulse sequences were used for the 1D and 2D experiments: ^1H-^1H COSY: 512x512 data matrix size; time domain 512 in Fl and 1024 in F2; relaxation delay (rd)= 2s; number of scans (ns)=8; dummy scans (ds)= 4; ^1H-^1H TOCSY: data matrix size 512x512; time domain 512 in Fl and 1024 in F2; rd= 3s; ns= 8; ds= 8; mixing time 87 ms. NOESY: data matrix size 512x512; time domain 512 in Fl and 1024 in F2; rd= 2s; ns= 40; ds= 32, contact time 0.3 s.

Inv4lplrnd: 512x512 matrix size, time domain in Fl 1K, in F2 512; ds= 4, ns= 96, rd=1s, low pass filter 0.0033s, delay for evolution 0.1s; hxcobicp same as inv4lplrnd with delay corresponding to a J=150 Hz.

Low resolution electronic impact mass spectrometry data were obtained by TRIO 2000 Fisons at 70 eV, 400 μA and source temperature 200°C. All solvents were spectral grade for spectroscopy. The Mps was uncorrected and was perfomed using a Gallenkamp Melting Point Apparatus; the IR spectra was recorded on a Perkin-Elmer 399 instrument in $CHCl_3$ solution; UV spectra was

measured on a spectrophometer Kontron Uvikon 930 in MeOH solution. The optical rotatory was measured on a Perkin-Elmer 141 polarimeter. Analytical and preparative TLC were perfomed on SiO_2 (Silica gel, Merck plates F_{254}, 20 x 20 cm thickness 0,25 mm and 0,5 mm) and reversed phase Rp18 (Whatman F_{254}, 20 x 20 cm thickness 0,25 mm); the spots were visualized after air dried by exposure to uv light and/or exposition by iodine vapours.

Identification: F. proliferatum was isolated from infected corn ear rot in northern Italy and the strain deposited with number 1494 at the collection of Istituto di Tossine e Micotossine da parassiti vegetali (ITEM), Bari. The identification was confirmed by P.E. Nelson who deposited the strain in Fusarium Research Center, University Park, USA as accession number M-7308.

Isolation Procedures: Proliferin was product by inoculation with F. proliferatum ITEM 1494 on autoclaved yellow corn kernels. 250 g of fresh material was grinded in a waring-blender and extracted with 1,2 l of methanol-1% aqueous NaCl (55:45 v/v) for 48 hours. The suspension was filtered and the methanol was removed by distillation under reduced pressure. Then the water residue (500 ml) was extracted with n-hexane (250 ml) for three times to separate lipidic inactive fraction. The aqueous phase was extracted three times with CH_2Cl_2 (250 ml), obtaining an oily residue (426 mg). The residue was dissolved in 1 ml of $CHCl_3$-iPrOH (95-5 v/v) was applied to SiO_2 column (350 g, 100 cm high, 25 mm id.) and eluted with the same solvent. The fractions were monitored by TLC, in the same eluent, and visualized by uv light and iodine vapours. The omogenus fractions were combined, dried under vacuum and bioassayed for their activity. Fractions showed mycotoxic activity gave a crude residue of 80.7 mg that was further purified by preparative TLC run out with $CHCl_3$-MeOH(95-5 v/v). The fluorescent band was detected at 254 nm, scraped from the plates, extracted by MeOH and dried under vacuum. This procedure yielded 48 mg of pure product.

Proliferin: Adding n-hexane to a solution in ethyl acetate of pure compound an amorfous solid was obtained with mp 142-147 °C; $[\alpha]_D$ -35° (MeOH c=0.255). NMR spetra are described in the results and discussion. IR ν_{max} cm^{-1}: 1728 (C=O ester), 1708 (C=O ketone), 1663 (=C(OH)-C=O). λ_{max} nm 261 (ε= 6000)

Acetyl-proliferin: The acetyl-proliferin was obtained by reaction of proliferin (5 mg) with Ac_2O (500 µl) and Pyridine (500 µl) at room temperature

for 12 hours. After addition of cold MeOH, the mixture was dried under reduced pressure with the formation of azeotropic moisture benzene/pyridine: $[\alpha]_D$ -44° (MeOH c=0,135). IR ν_{max} cm^{-1}: 1728 (C=O ester), 1708 (C=O ketone), 1663 (=C(OH)-C=O). λ_{max} nm 228 (ε= 6500) and λ_{max} nm 207 (ε= 7200).

ACKNOWLEDGMENTS: This research was supported by C.N.R. special program "Chimica Fine II"; NMR service of the Area of the research of C.N.R. is also acknowledged.

REFERENCES AND NOTES

1) Nirenberg, H.; "Mitt. Biol. Bundesanst" Land-Forstwirtsch; Berlin-Dahlem, **1976**, *169*, 1.

2) Gerlach, W. and Nirenberg, H.; "Mitt. Biol. Bundesanst" Land-Forstwirtsch; Berlin-Dahlem, **1982**, *209*, 1.

3) Marasas, W.F.O.; Thiel, P.G; Rabie, C.J.; Nelson, P.E. and Toussoun, T.A.; *Mycologia*; **1986**, *78*, 242.

4) Ross, P.F.; Nelson, P.E.; Richard, J.L.; Osweiler, J.F.; Rice, L.G.; Plattner R.D. and Wilson T.M.; *Appl. Environ. Microbiol.*; **1990**, *56*, 3225.

5) Moretti, A.; Logrieco, A.; Bottalico, A.; Ritieni, A.; Randazzo, G.; Abs. of 6th International Congress of Plant Pathology, Montreal, **1993**, 442.

6) Neuhas ,D.; Keeler, J.; Freeman, R.; *J. Magn. Res.* **1985**, *61*, 553.

7) Morris, G.A. "Topics in ^{13}C NMR Spectroscopy" Wiley, New York **1984**; vol. 4.

8) Jeener, J.; Ampère International Summer School, Basko Polje, Yugoslavia **1971.**

9) Bax, A. and Davis, D.G.; *J. Magn Res.* **1985**, *65*, 355.

10) Bax, A; *J. Magn. Res.* **1983**, *53*, 517.

11) Rutar, V.; *J Magn. Res.* **1984**, *58*, 306.

12) Wilde, J.A. and Bolton, P.H.; *J. Magn. Res.* **1984**, *59*, 343.

13) Crews, P.; Jimenez, C.; O'Neil-Johnson, M.; *Tetrahedron*, **1991**, *47*, 585.

14) Bax, A and Summers, M.F.; *J. Am. Chem. Soc.*, **1986**, *108*, 2093.

15) Stothers, J.B. in "Carbon-13 NMR Spectroscopy"; Acad. Press, New York, **1972**, 348.

16) Silverstein, R.M.; Bassler C.G; and Morril C.T. in" Spectrometric identification of organic compounds"; Wiley & Sons, New York, **1981**, Fourth Edition

17) Nakanishi, K. in "Infrared Absorption Spectroscopy"; Holden-Day, San Francisco, **1962**, 17.

18) Ernst R.R.; Bodenhausen and Wokaun A. in "Principles of Nuclear Magnetic Resonance in One and Two Dimensions"; Oxford Sci., New York, **1987**
