# Supplementary material for: Investigation of the Anti-Inflammatory Activity of Fusaproliferin Analogues Guided by Transcriptome Analysis
Source: Front Pharmacol. 2022 May 5;13:881182. doi: 10.3389/fphar.2022.881182 (PMC10136769; doi:10.3389/fphar.2022.881182)
Supplement: Supplementary file 2 [file DataSheet2.ZIP › Compound 3 and compound 4 reference.pdf]

Sesterterpenes and 2*H*-Pyran-2-ones (= α-Pyrones) from the Mangrove-Derived Endophytic Fungus *Fusarium proliferatum* MA-84

by **Dong Liu**[a])[b]), **Xiao-Ming Li**[a]), **Chun-Shun Li**[a]), and **Bin-Gui Wang***[a])

[a]) Key Laboratory of Experimental Marine Biology, Institute of Oceanology, Chinese Academy of Sciences, Nanhai Road 7, Qingdao 266071, P. R. China
(phone: + 86-532-82898553; fax: + 86-532-82880645; e-mail: wangbingui@gmail.com)
[b]) Graduate School of the Chinese Academy of Sciences, Yuquan Road 19A, Beijing 100049, P. R. China

Two new tricyclic sesterterpenes, fusaprolifins A and B (**1** and **2**), and three new 2*H*-pyran-2-one derivatives, prolipyrones A – C (**3** – **5**), were isolated and characterized from *Fusarium proliferatum* MA-84, an endophytic fungus obtained from the fresh tissue of the marine mangrove plant *Bruguiera sexangula*. In addition, two known sesterterpenes, terpestacin (**6**) and fusaproliferin (**7**), and one known 2*H*-pyran-2-one derivative, gibepyrone D (**8**), were also identified. The structures of these compounds were elucidated by detailed spectroscopic analyses. Fusaprolifin A (**1**) showed moderate activity against brine shrimp (*Artemia salina*), with a lethality rate of 49.5% at 100 μg/ml, while fusaprolifin B (**2**) showed weak activity.

Introduction. – Marine microorganisms, especially marine-derived fungi, are known to be a rich source of structurally interesting and biologically active compounds [1 – 3]. In recent years, chemical investigations of mangrove-derived endophytic fungi, especially those from the subtropical Hainan Island, China, have shown a sharp increase [4 – 6]. During our ongoing search for bioactive metabolites from marine-derived fungi [7 – 15], a fungal strain of *Fusarium proliferatum* MA-84, isolated from the inner tissue of the marine mangrove plant *Bruguiera sexangula* collected from Hainan Island, China, was found to have potent brine-shrimp lethality activity. An organic extract of the rice solid culture led to the isolation of five new secondary metabolites including two new sesterterpenes, fusaprolifins A and B[1]) (**1** and **2**), and three new 2*H*-pyran-2-ones, prolipyrones A – C[1]) (**3** – **5**). In addition, two known sesterterpenes, terpestacin (**6**) [16] and fusaproliferin (**7**) [17], and one known 2*H*-pyran-2-one derivative, gibepyrone D (**8**) [18][19], were also isolated and identified. The antimicrobial and brine shrimp (*Artemia salina*) lethality assays of the isolated compounds were evaluated. This paper describes the isolation, structure determination, and bioactivity of compounds **1** – **8**.

Results and Discussion. – The AcOEt extract derived from the rice culture of the fungal strain *F. proliferatum* MA-84 was suspended in MeOH/H_2O (9 : 1) and was extracted with hexane to remove the nonpolar fraction. The MeOH-soluble fraction was then concentrated and further purified by a combination of column chromatog-

[1]) Trivial or arbitary numbering; for systematic names, see *Exper. Part*.

© 2013 Verlag Helvetica Chimica Acta AG, Zürich

raphy (SiO_2, *Sephadex LH-20*) and semipreparative HPLC to yield the five new metabolites **1**–**5** and the three known metabolites **6**–**8** (*Fig. 1*).

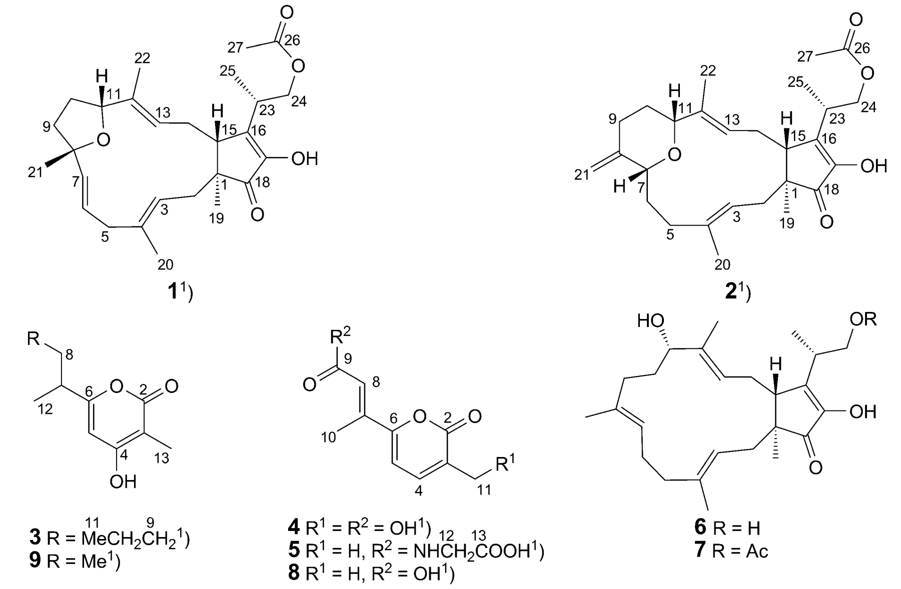

Fig. 1. *Isolated compounds* **1**–**8** *and reference compound phomapyrone C* (**9**)

Compound **1** was obtained as colorless amorphous solid. Low-resolution ESI-MS displayed a quasimolecular-ion peak at *m/z* 443 ($[M+H]^+$). The molecular formula was determined as $C_{27}H_{38}O_5$ on the basis of the positive-mode HR-ESI-MS, implying nine degrees of unsaturation. The ^{1}H-NMR spectrum (*Table 1*) exhibited diagnostic signals for one CH–O group at δ(H) 3.96 (H–C(11)), one CH_2O group at δ(H) 4.24–4.30 (CH_2(24)), six Me groups at δ(H) 1.03 (Me(19)), 1.30 (Me(25)), 1.34 (Me(21)), 1.59 (Me(22)), 1.70 (Me(20)), and 2.03 (Me(27)), and four olefinic H-atoms at δ(H) 5.17 (H–C(3)), 5.29 (H–C(13)), 5.49 (H–C(7)), and 5.76 (H–C(6)). The ^{13}C-NMR (DEPT) data (*Table 1*) revealed the presence of 27 C-atoms including six Me, six CH_2, and seven CH groups, and eight quaternary C-atoms. Detailed analyses of the 1D- and 2D-NMR data of **1** (*Table 1*) and comparison with those of fusaproliferin (**7**), a toxic sesterterpene from the fungal strain of *F. proliferatum* [17], revealed that the structures of these two compounds were similar, except for several apparent differences. Comparison of the ^{13}C-NMR spectra of **1** and fusaproliferin (**7**) showed that the CH_2(6) C-atom of **7** at δ(C) 23.8 was replaced by an olefinic CH(6) at δ(C) 124.6 in **1**. Accordingly, in the ^{1}H-NMR spectrum of **1** the CH_2(6) group was missing, and the olefinic H–C(6) was observed at δ(H) 5.76. At the same time, the olefinic quaternary C(8) of **7** at δ(C) 132.9 was replaced by the oxygenated quaternary C(8) at δ(C) 72.9 in **1**. The above evidence indicated that the structure of compound **1** had the C=C bond transposed from C(7) to C(6) compared to **7**. In addition, an O-bridge was positioned between C(8) and C(11) of **1**, based on the fact that the molecular formula of both compounds **1** and **7** contained five O-atoms but compound **1** exhibited an additional

Table 1. *^{1}H- and ^{13}C-NMR Data* (500 and 125 MHz, resp.; $CDCl_3$) *of* **1** *and* **2**[1]). δ in ppm, *J* in Hz.

Position	**1**		**2**	
	δ(H)	δ(C)	δ(H)	δ(C)
C(1)		48.1 (*s*)		48.7 (*s*)
CH_2(2)	2.20–2.23 (*m*, H_α), 1.80–1.85 (*m*, H_β)	38.4 (*t*)	2.25–2.29 (*m*, H_α), 1.69–1.75 (*m*, H_β)	38.7 (*t*)
H–C(3)	5.17 (br. *d*, *J* = 6.9)	118.7 (*d*)	5.06–5.09 (*m*)	119.8 (*d*)
C(4)		137.1 (*s*)		138.4 (*s*)
CH_2(5)	2.79 (br. *d*, *J* = 6.6)	41.3 (*t*)	2.09 (*t*, *J* = 7.5)	34.5 (*t*)
H–C(6) or CH_2(6)	5.76 (*dt*, *J* = 15.5, 6.6)	124.6 (*d*)	1.80–1.83 (*m*, H_α), 1.60–1.63 (*m*, H_β)	32.0 (*t*)
H–C(7)	5.49 (*d*, *J* = 15.5)	136.4 (*d*)	4.04 (*t*, *J* = 5,8)	73.6 (*d*)
C(8)		72.9 (*s*)		150.4 (*s*)
CH_2(9)	1.81–1.83 (*m*, H_α), 1.68–1.70 (*m*, H_β)	37.1 (*t*)	2.33–2.37 (*m*, H_α), 1.84–1.88 (*m*, H_β)	29.4 (*t*)
CH_2(10)	1.54–1.56 (*m*, H_α), 1.63–1.65 (*m*, H_β)	29.2 (*t*)	1.64–1.69 (*m*, H_α), 1.81–1.85 (*m*, H_β)	33.5 (*t*)
H–C(11)	3.96 (*dd*, *J* = 10.2, 2.2)	78.4 (*d*)	4.15 (*t*, *J* = 6.5)	76.4 (*d*)
C(12)		137.8 (*s*)		137.8 (*s*)
H–C(13)	5.29 (*dd*, *J* = 8.9, 3.0)	125.3 (*d*)	5.36 (*dd*, *J* = 9.0, 3.3)	127.3 (*d*)
CH_2(14)	1.84–1.87 (*m*, H_α), 2.31–2.36 (*m*, H_β)	28.2 (*t*)	1.92–1.96 (*m*, H_α), 2.24–2.28 (*m*, H_β)	28.8 (*t*)
H–C(15)	2.45 (*dd*, *J* = 11.3, 1.2)	49.2 (*d*)	2.55 (*dd*, *J* = 10.2, 2.4)	50.0 (*d*)
C(16)		146.4 (*s*)		146.9 (*s*)
C(17)		146.0 (*s*)		146.6 (*s*)
C(18)		207.0 (*s*)		207.5 (*s*)
Me(19)	1.03 (*s*)	15.2 (*q*)	0.96 (*s*)	16.4 (*q*)
Me(20)	1.70 (*s*)	17.4 (*q*)	1.63 (*s*)	16.6 (*q*)
Me(21) or CH_2(21)	1.34 (*s*)	29.8 (*q*)	5.06 (*s*), 5.21 (*s*)	110.3 (*t*)
Me(22)	1.59 (*s*)	11.0 (*q*)	1.61 (*s*)	11.3 (*q*)
H–C(23)	2.76–2.78 (*m*)	33.2 (*d*)	2.76–2.79 (*m*)	33.7 (*d*)
CH_2(24)	4.24–4.30 (*m*)	65.8 (*t*)	4.21–4.29 (*m*)	66.4 (*t*)
Me(25)	1.30 (*d*, *J* = 7.0)	14.0 (*q*)	1.30 (*d*, *J* = 7.1)	14.6 (*q*)
C(26)		170.3 (*s*)		170.8 (*s*)
Me(27)	2.03 (*s*)	20.2 (*q*)	2.01 (*s*)	20.8 (*q*)
OH	5.67 (br. *s*)		5.55 (*s*)	

oxygenated quaternary C-atom at δ(C) 72.9 (C(8)) in its ^{13}C-NMR spectrum. The ^{1}H,^{1}H-COSY (*Fig. 2*) cross-peaks CH_2(10)/CH_2(9)/H–C(11) as well as the HMBCs (*Fig. 2*) Me(21)/C(7), C(8), and C(9) and Me(22)/C(11), C(12), and C(13) supported the above deduction. The relative configuration of **1** was determined by analyses of coupling constants and by NOESY experiments as well as by comparison with the literature data. The large coupling constants for H–C(6) and H–C(7) (*J* = 15.5 Hz) and for H_α–C(14) and H–C(15) (*d*, *J* = 11.3 Hz) indicated the *trans* relationship for the two H-atom pairs. In the NOESY experiment, Me(19) displayed an NOE correlation to H_α–C(14) but not to H–C(15) (*Fig. 3*), suggesting a *trans* fusion of the 5- and 15-membered rings. In addition, the NOEs H–C(3)/H–C(7) and H–C(13) and H_α–C(14)/Me(22), as well as comparison with the literature data [17], established (*E*)-geometries

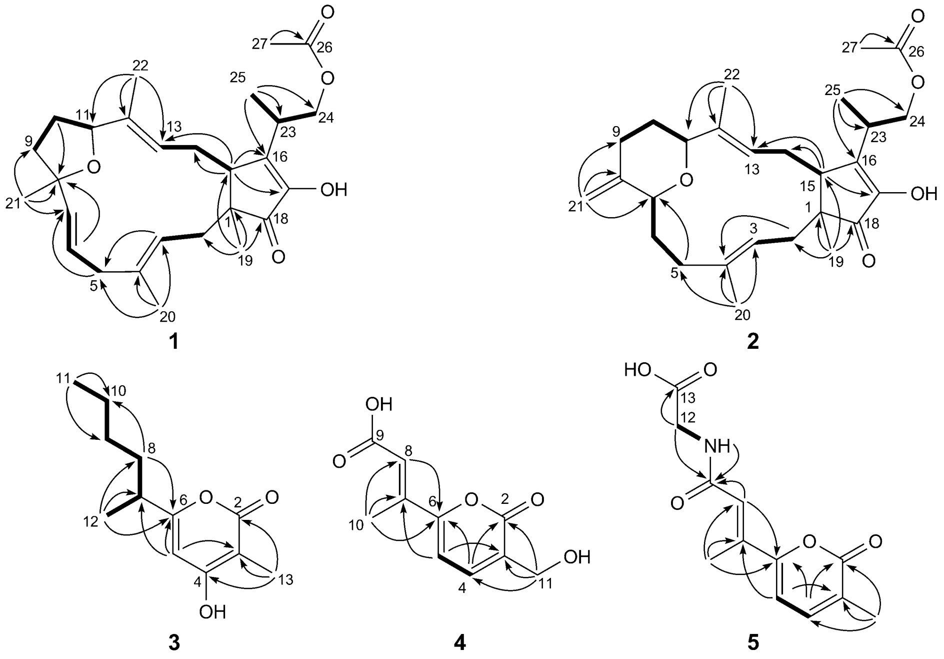

Fig. 2. *Key COSY* (—) *and HMBC* (H → C) *features of compounds* **1**–**5**[1])

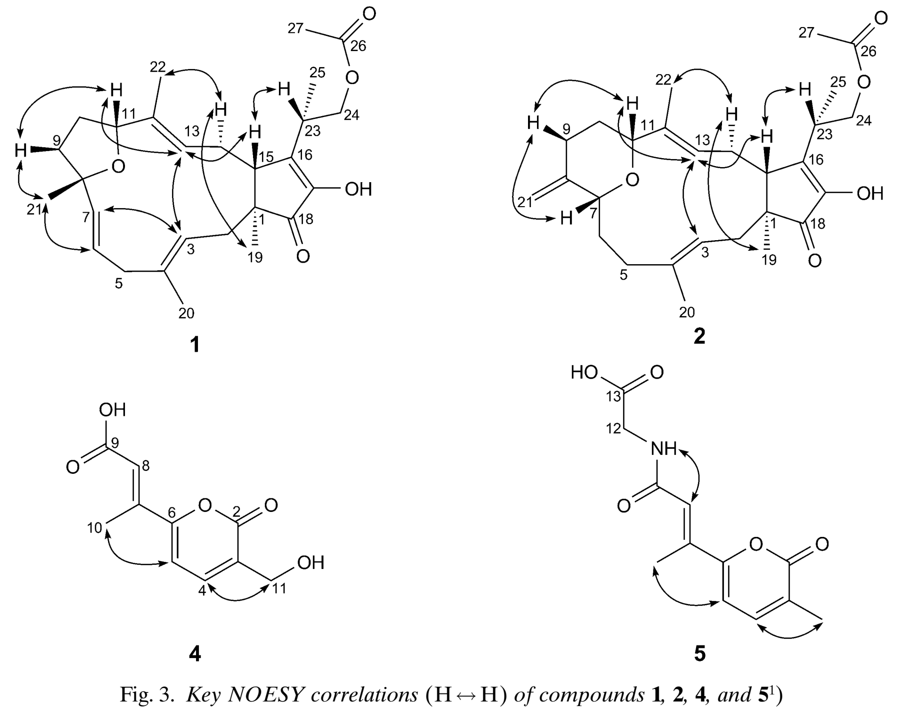

Fig. 3. *Key NOESY correlations* (H ↔ H) *of compounds* **1**, **2**, **4**, *and* **5**[1])

for the C=C bonds at C(3) and C(12). Furthermore, the NOEs H_β–C(9)/Me(21) and H–C(11), H–C(13)/H–C(11) and H–C(15), and H–C(23)/H–C(15) indicated the β-configurations of H–C(11), H–C(15), Me(21), and H–C(23). Based on the above evidence, the structure of compound **1** was assigned, and it was named fusaprolifin A.

Compound **2**, another colorless amorphous solid, was assigned the molecular formula $C_{27}H_{38}O_5$, the same as that of **1**, on the basis of HR-ESI-MS data. The NMR and UV spectra of **2** were very similar to those of **1**, indicating that **2** had the same skeleton as **1**. However, in the ^{13}C-NMR spectrum of **2** (*Table 1*), the two olefinic CH groups at δ(C) 124.6 (C(6)) and 136.4 (C(7)) of **1** were replaced by an aliphatic CH_2 group at δ(C) 32.0 (C(6)) and a CH–O group at δ(C) 73.6 (C(7)), respectively. Moreover the oxygenated quaternary C-atom and Me(21) group of **1** at δ(C) 72.9 (C(8)) and 29.8 (C(21)), respectively, were replaced in **2** by an olefinic quaternary C-atom and a terminal olefinic CH_2 group at δ(C) 150.4 (C(8)) and 110.3 (C(21)), respectively. Accordingly, the olefinic H–C(6) and H–C(7) of **1** at δ(H) 5.76 and 5.49, respectively, and the Me(21) of **1** at δ(H) 1.34 were replaced in **2** by the corresponding CH_2(6) (δ(H) 1.60–1.63 and 1.80–1.83), the oxymethine (H–C(7) (δ(H) 4.04), and two terminal olefinic H-atoms (δ(H) 5.06 (H_a–C(21)) and 5.21 (H_b–C(21)); *Table 1*). The above evidence indicated a shift of the C=C bond from C(6)=C(7) in **1** to C(8)=C(21) in **2** as well the transformation of the furan ring in **1** to a pyran ring in **2**. The ^{1}H,^{1}H-COSY cross-peaks CH_2(10)/CH_2(9)/H–C(11) (*Fig. 2*) and the HMBCs H_a–C(21)/C(7), C(8), and C(9) and Me(22)/C(11), C(12), and C(13) (*Fig. 2*) supported the above deduction. The relative configuration of **2** was also determined by analyses of coupling constants and by NOESY experiments as well as by comparison with the literature data. The large coupling constant for H_α–C(14)/H–C(15) (J = 10.2 Hz) indicated their *trans* relationship. Moreover, the obvious NOE correlation Me(19)/H_α–C(14) (*Fig. 3*) suggested a *trans* fusion of the 5- and 15-membered rings. The NOE correlations H–C(3)/H–C(13) and H_α–C(14)/Me(22), as well as comparison with the literature data [16] [17], established (*E*)-geometries for the two C=C bonds at C(3) and C(12). In addition, the NOE correlations H_β–C(9)/H–C(7) and H–C(11), H–C(13)/H–C(11) and H–C(15), and H–C(23)/H–C(15) indicated the β-configurations of these H-atoms. The structure of compound **2** was thus assigned, and it was named as fusaprolifin B.

Prolipyrone A (**3**), also obtained as a colorless amorphous solid, was determined to have the molecular formula $C_{12}H_{18}O_3$ by HR-ESI-MS, with four degrees of unsaturation. Detailed analyses of the NMR data (*Table 2*) suggested that **3** had a similar structure to phomapyrone C (**9**) [20]. The only difference was that the Me substitution at C(8) in **9** was replaced by a propyl group in **3**. The ^{1}H,^{1}H-COSY plot indicated the presence of the side-chain fragments from C(7) to C(12) and from C(7) to C(11) (*Fig. 2*). Additionally, the observed HMBC cross-peaks Me(11)/C(9) and C(10) and Me(12)/C(6), C(7), and C(8) supported the above deduction, and established that the side chain was located at C(6). The structure of **3** was therefore determined, and it was named prolipyrone A.

Compound **4** was also obtained as colorless amorphous powder. Its molecular formula was determined to be $C_{10}H_{10}O_5$ by HR-ESI-MS, having six degrees of unsaturation. Detailed analyses of the NMR data of **4** (*Table 2*) and gibepyrone D (**8**) [18] [19] suggested that the structures of these two compounds were very similar, except

Table 2. *^{1}H- and ^{13}C-NMR Data* (500 and 125 MHz, resp.; (D_6)DMSO) *of* **3**–**5**[1]). δ in ppm, *J* in Hz)

Position	**3**		Position	**4**		**5**	
	δ(H)	δ(C)		δ(H)	δ(C)	δ(H)	δ(C)
C(2)		165.4 (*s*)	C(2)		159.7 (*s*)		161.5 (*s*)
C(3)		94.5 (*s*)	C(3)		130.0 (*s*)		125.3 (*s*)
C(4)		169.0 (*s*)	H–C(4)	7.52 (*dt*, *J* = 7.0, 1.4)	137.2 (*d*)	7.44 (*dd*, *J* = 6.9, 1.2)	139.9 (*d*)
H–C(5)	5.80 (*s*)	100.4 (*d*)	H–C(5)	6.85 (*d*, *J* = 7.0)	105.6 (*d*)	6.65 (*d*, *J* = 6.9)	105.4 (*d*)
C(6)		164.7 (*s*)	C(6)		156.6 (*s*)		157.1 (*s*)
H–C(7)	2.38 – 2.45 (*m*)	37.1 (*d*)	C(7)		140.7 (*s*)		137.8 (*s*)
CH_2(8)	1.34 – 1.41, 1.47 – 1.55 (2*m*)	33.6 (*t*)	H–C(8)	6.45 (*s*)	119.7 (*d*)	6.69 (br. *s*)	121.1 (*d*)
CH_2(9)	1.13 – 1.28 (*m*)	28.7 (*t*)	C(9)		167.1 (*s*)		165.5 (*s*)
CH_2(10)	1.21 – 1.29 (*m*)	21.9 (*t*)	Me(10)	2.30 (*s*)	13.0 (*q*)	2.32 (*d*, *J* = 0.8)	12.6 (*q*)
Me(11)	0.84 (*t*, *J* = 7.2)	13.7 (*q*)	CH_2(11) or Me(11)	4.30 (br. *s*)	57.9 (*t*)	2.03 (br. *s*)	16.1 (*q*)
Me(12)	1.07 (*d*, *J* = 6.9)	18.2 (*q*)	CH_2(12)			3.82 (*d*, *J* = 5.8)	40.6 (*t*)
Me(13)	1.68 (*s*)	8.6 (*q*)	C(13)				171.0 (*s*)
			NH			8.70 (*t*, *J* = 5.8)	

for the Me group of **8** at δ(H) 2.10 and δ(C) 17.5 (Me(11); measured in CD_3OD) [19] being replaced in **4** by a CH_2O group at δ(H) 4.30 and δ(C) 57.9 (CH_2(11)). This was supported by the HMBCs CH_2(11)/C(2), C(3), and C(4) (*Fig. 2*). Additionally, the correlations Me(10)/C(6), C(7), and C(8) and H–C(4)/C(2) and C(6) were also observed in the HMBC spectrum of **4**. The (*E*)-geometry for the C=C bond at C(7) was established by the observed NOE correlation Me(10)/H–C(5) and by the lacking NOE correlation H–C(8)/Me(10). The structure of compound **4** was therefore determined, and it was named prolipyrone B.

Compound **5** was obtained as colorless amorphous powder. The molecular formula was determined as $C_{12}H_{13}NO_5$ on the basis of its positive-mode HR-ESI-MS, with seven degrees of unsaturation. The NMR and UV spectra of **4**, **5**, and **8** were similar to each other, indicating that the three compounds had the same basic C-atom skeleton. However, detailed analyses of the ^{13}C-NMR (DEPT) data (*Table 2*) showed that **5** had an additional CH_2 and C=O group appearing at δ(C) 40.6 (C(12)) and 171.0 (C(13)), respectively, when comparing with that of gibepyrone D (**8**) [18][19]. In addition, the CH_2 signals at δ(H) 3.82 (CH_2(12)) and the signal of an exchangable H-atom at δ(H) 8.70 (NH) were also observed in the ^{1}H-NMR spectrum of **5** (*Table 2*). Moreover, the ^{1}H,^{1}H-COSY cross-peak CH_2(12)/NH and the HMBCs NH/C(9), and CH_2(12)/C(9) and C(13), Me(10)/C(6), C(7), and C(8), H–C(8)/C(6) and C(9), Me(11)/C(2), C(3), and C(4), and H–C(4)/C(2) and C(6) (*Fig. 2*), allowed the structure determination of **5**. The observed NOE correlations H–C(5)/Me(10) and H–C(8)/NH established the relative configuration of **5**. This compound was assigned the trival name prolipyrone C.

The antimicrobial activity of compounds **1**–**8** against two bacteria and four plant-pathogen fungi was evaluated. No obvious activity could be observed for these compounds. The lethality activity of the new compounds **1**–**5** against brine shrimp

(*Artemia salina*) was also evaluated. Fusaprolifins A and B (**1** and **2**) showed moderate and weak activity, with lethality rates of 49.5% and 9.6%, at 100 μg/ml, respectively.

This work was financially supported by the *Natural Science Foundation of China* (30910103914) and by the *Ministry of Science and Technology of China* (2010CB833800).

Experimental Part

General. TLC: precoated silica gel plates (SiO_2 *GF-254*; *Qingdao Haiyang Chemical Factory*). Column chromatography (CC): commercial silica gel (SiO_2; 100–200 and 200–300 mesh; *Qingdao Haiyang Chemical Factory*) and *Sephadex LH-20* (*Pharmacia*). Anal. HPLC: *Dionex-P680* HPLC system comprised of a *P680* pump, an *ASI-100* automated sample injector, a *TCC-100* column oven, a *UV-DAD-340U* detector, and a *Dionex-Acclaim-ODS* column (5 μm; 4.6 × 250 mm). Semi-prep. HPLC: *Dionex-UltiMate-U3000* system with UV detection; *Elite-ODS-BP* column (10 μm; 10.0 × 300 mm); t_R in min. Optical rotations: *Optical-Activity-AA-55* polarimeter. UV Spectra: *Gold-Spectrumlab-54* UV/ViS spectrophotometer (*Shanghai Lengguang Tech. Co.*); λ_{max} (log ε) in nm. NMR Spectra: *Bruker-Avance-500* spectrometer; at 500 (^1H) and 125 MHz (^{13}C); δ in ppm rel. to Me_4Si as internal standard, J in Hz. Low- and high-resolution ESI-MS: *VG-Autospec-3000* mass spectrometer; in *m*/*z*.

Fungal Isolation and Identification. The endophytic fungus *Fusarium proliferatum* MA-84 was isolated from the fresh tissue of the marine mangrove plant *Bruguiera sexangula* which was collected from Hainan Island of China. Fungal identification was performed based on sequencing of the ITS regions as previously reported [15]. The sequence data of 5.8S rDNA and ITS regions derived from the fungal strain has been deposited at GenBank with accession No. JQ693101. The nucleotide BLAST search result showed that the sequence was the most similar (99%) to the sequence of *Fusarium proliferatum* CMU16 (compared to accession No. GQ924905). The strain *F. proliferatum* MA-84 is preserved at the Key Laboratory of Experimental Marine Biology, Institute of Oceanology, Chinese Academy of Sciences.

Fermentation, *Extraction*, *and Isolation.* Mass growth of the fungus for the isolation and identification of secondary metabolites was carried out in *Erlenmeyer* flasks (1 l each). The fungus was grown on rice solid medium (to commercially available rice (100 g) was added peptone (0.6 g) and sea water (100 ml), and the mixture was kept overnight prior to autoclaving) at r.t. under static conditions for 30 d.

The rice culture (60 flasks) of the fungal strain was extracted with AcOEt. The crude extract was dried and partitioned between hexane and 90% MeOH. The 90% MeOH-soluble material (20 g) was subjected to vacuum liquid chromatography (VLC; SiO_2, different solvents of increasing polarity from petroleum ether to MeOH): *Fractions. 1–10*, (TLC monitoring). *Fr. 5* (2.0 g) was further purified by CC (SiO_2, petroleum ether/AcOEt 10:1 → 0:1, then *Sephadex-LH-20*, MeOH), and by semi-prep. HPLC (*Elite ODS-BP*, 80% MeOH/H_2O, 3 ml/min): **1** (8.0 mg; t_R 16.0), **2** (3.2 mg; t_R 20.9), **6** (10.0 mg; t_R 22.0), and **7** (50.0 mg; t_R 25.3). *Fr. 7* (1.0 g) was further purified by CC (SiO_2, $CHCl_3$/MeOH 0:1 → 1:1, then *Sephadex LH-20*, MeOH), and semi-prep. HPLC (40% MeOH/H_2O, 3 ml/min): **3** (3.0 mg; t_R 22.0), **4** (8.0 mg; t_R 12.6), **5** (6.0 mg; t_R 18.3), and **8** (10.0 mg; t_R 10.9).

Fusaprolifin A (= *rel-(3a*R*,5*E*,7*R*,10*S*,11*E*,14*E*,16a*S*)-3-[(1*S*)-2-(Acetyloxy)-1-methylethyl]-4,7,8,9,10,13,16,16a-octahydro-2-hydroxy-6,10,14,16a-tetramethyl-7,10-epoxycyclopentacyclopentadecen-1(3a*H*)-one*; **1**): Colorless amorphous solid. $[\alpha]_D^{20} = -8.4$ ($c = 0.8$, MeOH). UV (MeOH): 263 (3.96). ^1H- and ^{13}C-NMR: *Table 1.* HR-ESI-MS (pos.): 443.2798 ($[M+H]^+$, $C_{27}H_{39}O_5^+$; calc. 443.2797).

Fusaprolifin B (= *rel-(3a*R*,5*E*,7*R*,11*R*,14*E*,16a*S*)-3-[(1*S*)-2-(Acetyloxy)-1-methylethyl]-4,7,8,9,10,11,12,13,16,16a-decahydro-2-hydroxy-6,14,16a-trimethyl-10-methylene-7,11-epoxycyclopentacyclopentadecen-1(3a*H*)-one*; **2**): Colorless amorphous solid. $[\alpha]_D^{20} = +11.1$ ($c = 0.4$, MeOH). UV (MeOH): 263 (3.92). ^1H- and ^{13}C-NMR: *Table 1.* HR-ESI-MS (pos.): 443.2794 ($[M+H]^+$, $C_{27}H_{39}O_5^+$; calc. 443.2797).

*Prolipyrone A (=4-Hydroxy-3-methyl-6-(1-methylpentyl)-2*H*-pyran-2-one*; **3**): Colorless amorphous solid. $[\alpha]_D^{20} = -20.0$ ($c = 0.3$, MeOH). UV (MeOH): 212 (4.08), 266 (3.68). ^1H- and ^{13}C-NMR: *Table 2.* HR-ESI-MS (pos.): 233.1152 ($[M+Na]^+$, $C_{12}H_{18}NaO_3^+$; calc. 233.1153).

Prolipyrone B (= *(2*E*)-3-[3-(Hydroxymethyl)-2-oxo-2*H*-pyran-6-yl]but-2-enoic Acid*; **4**): Colorless amorphous powder. UV (MeOH): 239 (3.74), 330 (3.77). ^{1}H- and ^{13}C-NMR: *Table 2*. HR-ESI-MS (pos.): 211.0603 ($[M + H]^+$, $C_{10}H_{11}O_5^+$; calc. 211.0606).

Prolipyrone C (= N-*[(2*E*)-3-(3-Methyl-2-oxo-2*H*-pyran-6-yl)-1-oxobut-2-en-1-yl]glycine;* **5**): Colorless amorphous powder. UV (MeOH): 238 (4.02), 330 (4.10). ^{1}H- and ^{13}C-NMR: *Table 2*. HR-ESI-MS (pos.): 274.0694 ($[M + Na]^+$, $C_{12}H_{13}NNaO_5^+$; calc. 274.0691).

Antimicrobial Activity. The antimicrobial activity was tested against two bacteria (*Staphylococcus aureus* and *Escherichia coli*) and four plant-pathogen fungi (*Alternaria brassicae*, *Fusarium oxysporum*, *Coniella diplodiella*, and *Physalospora piricola*) and compared to the positive controls chloramphenicol and amphotericin B, resp. [21].

Brine-Shrimp Lethality Activity. The brine-shrimp lethality assay was performed with *Artemia salina* according to a standard protocol [22].

REFERENCES

[1] J. W. Blunt, B. R. Copp, W.-P. Hu, M. H. G. Munro, P. T. Northcote, M. R. Prinsep, *Nat. Prod. Rep.* **2009**, *26*, 170.
[2] J. W. Blunt, B. R. Copp, M. H. G. Munro, P. T. Northcote, M. R. Prinsep, *Nat. Prod. Rep.* **2010**, *27*, 165.
[3] J. W. Blunt, B. R. Copp, M. H. G. Munro, P. T. Northcote, M. R. Prinsep, *Nat. Prod. Rep.* **2011**, *28*, 196.
[4] M. E. Rateb, R. Ebel, *Nat. Prod. Rep.* **2011**, *28*, 290.
[5] L. Li, I. Sattler, Z. Deng, I. Groth, G. Walther, K.-D. Menzel, G. Peschel, S. Grabley, W. Lin, *Phytochemistry* **2008**, *69*, 511.
[6] A. H. Aly, A. Debbab, J. Kjer, P. Proksch, *Fungal Diversity* **2010**, *41*, 1.
[7] H.-J. Yan, X.-M. Li, C.-S. Li, B.-G. Wang, *Helv. Chim. Acta* **2012**, *95*, 163.
[8] D. Liu, X.-M. Li, L. Meng, C.-S. Li, S.-S. Gao, Z. Shang, P. Proksch, C.-G. Huang, B.-G. Wang, *J. Nat. Prod.* **2011**, *74*, 1787.
[9] C.-S. Li, C.-Y. An, X.-M. Li, S.-S. Gao, C.-M. Cui, H.-F. Sun, B.-G.Wang, *J. Nat. Prod.* **2011**, *74*, 1331.
[10] S.-S. Gao, X.-M. Li, Y. Zhang, C.-S. Li, C.-M. Cui, B.-G. Wang, *J. Nat. Prod.* **2011**, *74*, 256.
[11] C.-M. Cui, X.-M. Li, L. Meng, C.-S. Li, C.-G. Huang, B.-G. Wang, *J. Nat. Prod.* **2010**, *73*, 1780.
[12] C.-M. Cui, X.-M. Li, C.-S. Li, P. Proksch, B.-G. Wang, *J. Nat. Prod.* **2010**, *73*, 729.
[13] C.-M. Cui, X.-M. Li, C.-S. Li, H.-F. Sun, S.-S. Gao, B.-G. Wang, *Helv. Chim. Acta* **2009**, *92*, 1366.
[14] D.-L. Li, X.-M. Li, B.-G. Wang, *J. Microbiol. Biotechnol.* **2009**, *19*, 675.
[15] S. Wang, X.-M. Li, F. Teuscher, D.-L. Li, A. Diesel, R. Ebel, P. Proksch, B.-G. Wang, *J. Nat. Prod.* **2006**, *69*, 1622.
[16] M. Oka, S. Iimura, Y. Narita, T. Furumai, M. Konishi, T. Oki, Q. Gao, H. Kakisawa, *J. Org. Chem.* **1993**, *58*, 1875.
[17] A. Santini, A. Ritieni, V. Fogliano, G. Randazzo, L. Mannina, A. Logrieco, E. Benedetti, *J. Nat. Prod.* **1996**, *59*, 109.
[18] A. F. Barrero, J. E. Oltra, M. M. Herrador, E. Cabrera, J. F. Sanchez, J. F. Quílez, F. J. Rojas, J. F. Reyes, *Tetrahedron* **1993**, *49*, 141.
[19] Q.-X. Wang, S.-F. Li, F. Zhao, H.-Q. Dai, L. Bao, R. Ding, H. Gao, L.-X. Zhang, H.-A. Wen, H.-W. Liu, *Fitoterapia* **2011**, *82*, 777.
[20] M. Soledade, C. Pedras, V. M. Morales, J. L. Taylor, *Phytochemistry* **1994**, *36*, 1315.
[21] S. K. S. Al-Burtamani, M. O. Fatope, R. G. Marwah, A. K. Onifade, S. H. Al-Saidi, *J. Ethnopharmacol.* **2005**, *96*, 107.
[22] P. N. Solis, C. W. Wright, M. M. Anderson, M. P. Gupta, J. D. Phillipson, *Planta Med.* **1993**, *59*, 250.

Received April 15, 2012
